# Supplementary material for: Multicentre, national, investigator-initiated, randomised, parallel-group, register-based superiority trial to compare extended ECG monitoring versus standard ECG monitoring in elderly patients with ischaemic stroke or transient ischaemic attack and the effect on stroke, death and intracerebral bleeding: the AF SPICE protocol
Source: BMJ Open. 2023 Nov 23;13(11):e073470. doi: 10.1136/bmjopen-2023-073470 (PMC10668286; doi:10.1136/bmjopen-2023-073470)
Supplement: Supplementary data [file bmjopen-2023-073470supp001.pdf]

BMJ Publishing Group Limited (BMJ) disclaims all liability and responsibility arising from any reliance placed on this supplemental material which has been supplied by the author(s)

Supplementary Appendix

Table of contents

Trials in event rates power calculation 2
Appendix table Trials in ECG detection rate power calculation 3
Baseline data entered at inclusion 4
Baseline data collected from Riksstroke register 5
ECG Data collected by local investigator in standard arm 6
Data collected by local investigator in intervention arm 6
Sample size calculation 13
Patient consent form 16

Engdahl J, *et al. BMJ Open* 2023; 13:e073470. doi: 10.1136/bmjopen-2023-073470

Trials in event rates power calculation

	AF+/OAC+			AF+/OAC-			AF-/OAC-		
	Rate	Mean Age	Reference	Rate	Mean Age	Reference	Rate	Mean Age	Reference
**Stroke**	2,23%	70,2	Diener[1] 2010 D110	8,3%	71,7	Diener[2] 2012	5,40%	73	Netland el al 2019
	1,91%	70,8	Diener[1] 2010 D150				5,10%	66	Amarenco[3] 2016
	2,53%	70,4	Diener[1] 2010 W				3,60%	70,1	Andersen[4] 2015
	2,39%	71,7	Diener[2] 2012 A				5,40%		Wachter[5] 2017
	2,79%	71	Hankey[6] 2012 R						
	2,96%	71	Hankey[6] 2012 W						
	2,26%	70,1	Easton[7] 2012 A						
	3,17%	70,1	Easton[7] 2012 W						
	2,44%		Rost[8] 2016 E						
**All-cause mortality**	3,24%	70,2	Diener[1] 2010 D110	7,9%	71,7	Diener[2] 2012	10,70%	73	Netland el al 2019
	4,39%	70,8	Diener[1] 2010 D150				1,80%	66	Amarenco[3] 2016
	4,58%	70,4	Diener[1] 2010 W				10,50%	70,1	Andersen[4] 2015
	5,78%	71,7	Diener[2] 2012 A				4,80%		Wachter[5] 2017
	4,40%	71	Hankey[6] 2012 R				10,60%		Lip[9] 2017
	4,54%	71	Hankey[6] 2012 W						
	4,22%	70,1	Easton[7] 2012 A						
	4,77%	70,1	Easton[7] 2012 W						
	4,35%		Rost[8] 2016 E						
**Intracerebral bleeding**	0,25%	70,2	Diener[1] 2010 D110	1,6%	71,7	Diener[2] 2012	0,40%	66	Amarenco[3] 2016
	0,53%	70,8	Diener[1] 2010 D150						
	1,28%	70,4	Diener[1] 2010 W						
	1,17%	71,7	Diener[2] 2012 A						
	0,59%	71	Hankey[6] 2012 R						
	0,80%	71	Hankey[6] 2012 W						
	0,55%	70,1	Easton[7] 2012 A						
	1,49%	70,1	Easton[7] 2012 W						
	0,62%		Rost[8] 2016 E						
**Mortal bleeding**	0,26%	71	Hankey[6] 2012 R						
	0,49%	71	Hankey[6] 2012 W						

AF = Atrial Fibrillation OAC = Oral Anticoagulant

Appendix table Trials in ECG detection rate power calculation

Publication Short-term external ECG after stroke/TIA	Detection rate (%)
Jabaudon[10] 2004	5.0
Shafqat[11] 2004	2.4
Alhadramy[12] 2010	2.5
Rizos[13] 2010	2.5
Doliwa-Sobocinski[14] 2012	2.0
Lazarro[15] 2012	6.0
Gumbinger[16] 2012	1.0
Suissa[17] 2012	2.3
Thakkar[18] 2014	5.8
Bansil[19] 2004	4.7

Publication Long-term external ECG after stroke/TIA	Detection rate (%)
Barthelemy[20] 2003	14.3
Jabadoun[10] 2004	5.7
Wallman[21] 2007	14.2
Tayal[22] 2008	5.0
Elijiovich[23] 2009	20.0
Bhatt[24] 2011	24.2
Flint[25] 2012	11.0
Higgins[26] 2013	8.0
Miller[27] 2013	17.3
Gladstone[28] 2014	16.1
Sebasigari[29] 2017	11.7
Kaura[30] 2019	16.3

Engdahl J, *et al. BMJ Open* 2023; 13:e073470. doi: 10.1136/bmjopen-2023-073470

BMJ Publishing Group Limited (BMJ) disclaims all liability and responsibility arising from any reliance placed on this supplemental material which has been supplied by the author(s)

Baseline data entered at inclusion

Variable	Unit
Record ID (automatically generated)	
Sex	Male/Female
Date of birth	Date
Social Security Number	Special format
Date of Stroke/TIA	Date
Age	Years
Height	cm
Weight	kg
Hemoglobin	g/l
Estimated GFR	ml/min
Site identification	Choice from list
Consent Date	Date
Randomization date	Date

Engdahl J, *et al. BMJ Open* 2023; 13:e073470. doi: 10.1136/bmjopen-2023-073470

Baseline data collected from Riksstroke register

Reporting Hospital	Statin treatment on admission
Date of symptom onset	Aspirin treatment on admission
Time of symptom onset	Clopidogrel treatment on admission
Level of consciousness at admission	
NIHSS at admission	Dipyridamole treatment on admission
Number of days admitted	Other antiplatelet treatment on admission
Number of days in stroke unit	Hypertension treatment on admission
	Dipyridamole treatment on admission
Diagnosis	Warfarin treatment on admission
Age at admission	Apixaban treatment on admission
Sex	Dabigatran treatment on admission
Level of assistance	Rivaroxaban treatment on admission
Living alone	Edoxaban treatment on admission
Level of dependence	
Assisted dressing	Statin treatment at discharge
Assisted personal hygiene	Aspirin treatment at discharge
Independence in locomotion	Clopidogrel treatment at discharge
	Dipyridamole treatment at discharge
Previously diagnosed AF	Warfarin treatment at discharge
AF diagnosed on arrival	Hypertension treatment at discharge
Previously or newly diagnosed diabetes	Apixaban treatment at discharge
Hypertension (treated) on admission	Dabigatran treatment at discharge
Smoking status	Rivaroxaban treatment at discharge
CT brain scan performed during admission	Reason for withholding OAC treatment
MRI brain scan performed during admission	
Carotide artery ultrasound brain scan performed during admission	Edoxaban treatment at discharge
CT angiography performed during admission	
Longterm ECG (at least 24 h) performed during admission	
Thrombolytic treatment administered	
Type of ward at admission	

Engdahl J, *et al. BMJ Open* 2023; 13:e073470. doi: 10.1136/bmjopen-2023-073470

ECG Data collected by local investigator in standard arm

Variable	Unit
Inpatient ECG Telemetry used	Yes/No
Duration of ECG Telemetry	Hours
AF diagnosed during ECG Telemetry	Yes/No
Holter Recording used	Yes/No
Duration of Holter Recording	Hours
AF diagnosed during Holter	Yes/No

Data collected by local investigator in intervention arm

Variable	Unit
ePatch number 1 serial number	Integer
Date epatch number 1 recording start	Date
Additional epatch number 1 serial number	Integer
Site of application additional epatch number 1	Health care/self-application
Date for mailing out additional epatch number 1	Date
Date additional epatch number 1 recording start	Date
Reason for additional epatch	1. epatch detachment<br>2. Red light indicated<br>3. Poor signal quality<br>4. MRI examination

Engdahl J, *et al. BMJ Open* 2023; 13:e073470. doi: 10.1136/bmjopen-2023-073470

	5. Other radiology<br>6. Thoracic/neck surgery<br>7. Other reason
Additional epatch number 1 serial number	Integer
Site of application additional epatch number 1	Health care/self-application
Date for mailing out additional epatch number 1	Date
Date additional epatch number 1 recording start	Date
Reason for additional epatch number 1	1. epatch detachment<br>2. Red light indicated<br>3. Poor signal quality<br>4. MRI examination<br>5. Other radiology<br>6. Thoracic/neck surgery<br>7. Other reason
Mode of application epatch number 2	Health care/self-application
Date for mailing of epatch number 2 to participant	Date
Date of application epatch number 2	Date
Epatch number 2 serial number	Integer
First additional epatch number 2 serial number	Integer
Mode of application first additional epatch number 2	Health care/self-application
Date for mailing of first additional epatch number 2 to participant	Date
Date of application of first additional epatch number 2	
Reason for first additional epatch number 2	1. epatch detachment<br>2. Red light indicated<br>3. Poor signal quality<br>4. MRI examination<br>5. Other radiology<br>6. Thoracic/neck surgery<br>7. Other reason

Second additional epatch number 2 serial number	Integer
Mode of application second additional epatch number 2	Health care/self-application
Date for mailing of second additional epatch number 2 to participant	Date
Date of application of second additional epatch number 2	
Reason for second additional epatch number 2	1. epatch detachment<br>2. Red light indicated<br>3. Poor signal quality<br>4. MRI examination<br>5. Other radiology<br>6. Thoracic/neck surgery<br>7. Other reason
Reason for not recording epatch number 2	1. Diagnosed with AF on epatch number 1<br>2. Participant declines recording<br>3. Participant decline further participation<br>4. Other reason

Engdahl J, *et al. BMJ Open* 2023; 13:e073470. doi: 10.1136/bmjopen-2023-073470

ECG Data collected in intervention group

**Patch number 1**	
**Variable**	**Unit**
Date for ECG data upload in Cardiologs	Date
Date for ECG reading	Date
Date for first additional ECG data upload in Cardiologs	Date
Date for first additional ECG reading	Date
Date for second additional ECG data upload in Cardiologs	Date
Date for second additional ECG reading	Date
Monitoring time	Hours
Analyzable time	Hours
Heart rate max	Bpm
Heart rate min	Bpm
Heart rate mean	Bpm
Proportion supraventricular ectopics	Percent
Proportion ventricular ectopics	Percent
Number of pauses > 2 seconds	Integer
Longest pause	Seconds, one decimal
AV-block II or III	Yes/No
Highest AV-block grade	1. AV-block II type 1<br>2. AV-block II type 2<br>3. AV-block III
Atrial fibrillation	Yes/No
Atrial flutter	Yes/No
Duration to first Atrial fibrillation/flutter	Hours
Atrial fibrillation/flutter subtype	1. Paroxysmal<br>2. Persistant
Atrial fibrillation/ flutter heart rate max	Bpm

Atrial fibrillation/ flutter heart rate mean	Bpm
Atrial fibrillation/ flutter burden	Percent
Atrial fibrillation/ flutter longest episode	Seconds
Number of Ventricular Tachycardia (VT)	n
Duration longest episode VT	Seconds
Number of Suptraventricular Tachycardia (SVT)	n
Duration longest episode SVT	Seconds
Proportion of noise	Percent
Action triggered by recording	1. Standard statement<br>2. Communication with local PI within 3 working days<br>3. Communication with local PI within one working day<br>4. Further assessment following patch number 2
**Patch number 2**	
**Variable**	**Unit**
Date for ECG data upload in Cardiologs	Date
Date for ECG reading	Date
Date for first additional ECG data upload in Cardiologs	Date
Date for first additional ECG reading	Date
Date for second additional ECG data upload in Cardiologs	Date
Date for second additional ECG reading	Date
Monitoring time	Hours
Analyzable time	Hours
Heart rate max	Bpm
Heart rate min	Bpm

Heart rate mean	Bpm
Proportion supraventricular ectopics	Percent
Proportion ventricular ectopics	Percent
Number of pauses > 2 seconds	Integer
Longest pause	Seconds, one decimal
AV-block II or III	Yes/No
Highest AV-block grade	1. AV-block II type 1<br>2. AV-block II type 2<br>3. AV-block III
Atrial fibrillation	Yes/No
Atrial flutter	Yes/No
Atrial fibrillation/flutter subtype	1. Paroxysmal<br>2. Persistant
Atrial fibrillation/ flutter heart rate max	Bpm
Atrial fibrillation/ flutter heart rate mean	Bpm
Atrial fibrillation/ flutter burden	Percent
Atrial fibrillation/ flutter longest episode	Seconds
Number of Ventricular Tachycardia (VT)	n
Duration longest episode VT	Seconds
Number of Suptraventricular Tachycardia (SVT)	n
Duration longest episode SVT	Seconds
Proportion of noise	Percent
Action triggered by recording	1. Standard statement<br>2. Communication with local PI within 3 working days<br>3. Communication with local PI within one working day

Engdahl J, *et al. BMJ Open* 2023; 13:e073470. doi: 10.1136/bmjopen-2023-073470

	4. Further assessment following patch number 2

Engdahl J, et al. BMJ Open 2023; 13:e073470. doi: 10.1136/bmjopen-2023-073470

Sample size calculation

Sample size calculation AF SPICE

Ida Hed Myrberg & Letizia Orsini

2021-10-08

Background

Sample size calculation for the AF SPICE study; a multicenter randomized register-based trail in Swedish stroke units.

Method

The function *cpower* in R package *Hmisc* is used. This is a function for calculating the power for a Cox Proportional Hazards model or log-rank test comparing two groups. The function assumes an underlying exponential distribution in both groups. To calculate the sample size needed to achieve 80% power, the function *uniroot* is used in combination with *cpower*.

How parameters are defined and calculated

In this section, it is shown how the yearly rate, and the 3-year event rate, of stroke/death/intracranial bleeding in the intervention and control group, respectively, are calculated based on the yearly rates in the three groups AF+/OAK+, AF+/OAK-, and AF-/OAK-, and on the proportions of these three groups in the intervention and control group. The proportion of AF+/OAK+, AF+/OAK-, and AF-/OAK- in the intervention and control group in Table 2 are examples, and in the next two sections they will be varied between 10-20% in the intervention group, and 3-12% in the control group. The yearly rate of stroke/death/intracranial bleeding for AF-/OAK- will be set to 5% and 8.4%, respectively, in two different scenarios.

Table 1 shows the assumed yearly rates of stroke/death/intracranial bleeding for the subgroups AF+/OAK+, AF+/OAK-, AF-/OAK-, while Table 2 shows the proportion of patients in each of the aforementioned subgroups, divided by interention and control.

Table 1: Yearly rate of stroke/death/intracranial bleeding depending on AF and treatment.

AF/treatment	Yearly rate of stroke/death/intracranial bleeding
AF+/OAK+	0.073
AF+/OAK-	0.177
AF-/OAK-	0.084

Table 2: Proportion of patients with AF and treatment in the intervention group and control group, respectively.

AF/treatment	Proportion in intervention group	Proportion in control group
AF+/OAK+	0.10	0.03
AF+/OAK-	0.00	0.07
AF-/OAK-	0.90	0.90

The yearly rate of stroke/death/intracranial in the intervention group is calculated as 0.073 * 0.1 + 0.177 * 0 + 0.084 * 0.9 = 0.0829. The yearly rate of stroke/death/intracranial in the control group is calculated as 0.073 * 0.03 + 0.177 * 0.07 + 0.084 * 0.9 = 0.09018.

Table 3: Yearly rate of stroke/death/intracranial bleeding in the intervention group and control group, respectively.

Group	Yearly rate of stroke/death/intracranial bleeding
Intervention group	0.0829
Control group	0.0902

The minimum follow-up time is assumed to be 3 years, and the recruitment time is set to 2 years.

The 3-year event rate in the control group, denoted *mc*, i.e. the proportion expected to have experienced the event by three years, is needed for the power calculation. The proportion not having experienced the event, i.e. 1-*mc*, is calculated using the Kaplan-Meier method, and the 3-year event rate is then calculated as 1-(1-*mc*)=*mc*. The 3-year event rate in the intervention group, *mi*, is calculated in a similar way. Both *mc* and *mi* are presented in Table 4.

Table 4: 3-year rate of stroke/death/intracranial bleeding in the intervention group and control group, respectively.

Group	3-year rate of stroke/death/intracranial bleeding
Intervention group	0.2287
Control group	0.2469

The percent reduction in the 3-year event rate for the intervention group compared to the control group is given by 1-22.87/24.69=7.38%.

Results: 5% yearly rate of stroke/death/intracranial bleeding for AF-/OAK-

In this section, a yearly event rate of 5% is assumed for the AF-/OAK- group.

The sample size needed is calculated for all combination of AF proportions, ranging from 10-20% in the intervention group, and 3-12% in the control group. Table 5 shows the percent reduction in the 3-year event rate for the intervention group compared to the control group, for different combinations of AF proportions.

Table 5: Percent reduction in the 3-year event rate for the intervention group compared to the control group. Columns: AF proportions in the control group. Rows: AF proportions in the intervention group.

	0.03	0.04	0.05	0.06	0.07	0.08	0.09	0.1	0.11	0.12
0.1	11.57	10.09	8.56	6.97	5.33	3.62	1.85	-	-	-
0.11	12.94	11.52	10.05	8.52	6.94	5.31	3.60	1.84	-	-

	0.03	0.04	0.05	0.06	0.07	0.08	0.09	0.1	0.11	0.12
0.12	14.25	12.89	11.47	10.01	8.49	6.92	5.28	3.59	1.83	-
0.13	15.52	14.20	12.83	11.42	9.96	8.45	6.89	5.26	3.57	1.82
0.14	16.73	15.46	14.14	12.78	11.38	9.92	8.42	6.86	5.24	3.56
0.15	17.89	16.66	15.40	14.09	12.73	11.33	9.88	8.38	6.83	5.21
0.16	19.01	17.82	16.60	15.34	14.03	12.68	11.29	9.84	8.35	6.80
0.17	20.09	18.94	17.76	16.54	15.28	13.98	12.63	11.24	9.80	8.31
0.18	21.12	20.01	18.87	17.69	16.48	15.22	13.92	12.58	11.20	9.76
0.19	22.12	21.05	19.94	18.80	17.63	16.41	15.16	13.87	12.53	11.15
0.2	23.08	22.04	20.97	19.87	18.73	17.56	16.35	15.10	13.82	12.48

Table 6 shows the total sample size needed to achieve 80% power to detect a statistically significant (with alpha=0.05) difference in the 3-year event rates presented in Table 5 between the intervention group and the control group, for different combinations of AF proportions.

Table 6: Total sample size needed for different combinations of AF proportions in the intervention and control group, respectively. Columns: AF proportions in the control group. Rows: AF proportions in the intervention group.

	0.03	0.04	0.05	0.06	0.07	0.08	0.09	0.1	0.11	0.12
0.1	8536	11504	16400	25370	44652	99460	393830	-	-	-
0.11	6628	8572	11552	16470	25480	44848	99900	395590	-	-
0.12	5310	6654	8608	11600	16542	25590	45044	100340	397348	-
0.13	4360	5332	6682	8644	11650	16612	25700	45240	100780	399108
0.14	3652	4378	5352	6710	8680	11698	16682	25810	45436	101220
0.15	3110	3668	4396	5374	6738	8716	11748	16752	25922	45630
0.16	2686	3124	3682	4412	5396	6764	8752	11796	16824	26032
0.17	2346	2696	3136	3696	4430	5418	6792	8788	11846	16894
0.18	2072	2356	2708	3148	3710	4448	5440	6820	8824	11894
0.19	1844	2080	2364	2718	3160	3726	4466	5462	6848	8860
0.2	1654	1850	2086	2374	2728	3172	3740	4484	5484	6876

Results: 8.4% yearly rate of stroke/death/intracranial bleeding for AF-/OAK-

In this section, a yearly event rate of 8.4% is assumed for the AF-/OAK- group.

The sample size needed is calculated for all combination of AF proportions, ranging from 10-20% in the intervention group, and 3-12% in the control group. Table 7 shows the percent reduction in the 3-year event rate for the intervention group compared to the control group, for different combinations of AF proportions.

Table 7: Percent reduction in the 3-year event rate for the intervention group compared to the control group. Columns: AF proportions in the control group. Rows: AF proportions in the intervention group.

	0.03	0.04	0.05	0.06	0.07	0.08	0.09	0.1	0.11	0.12
0.1	7.38	6.40	5.40	4.37	3.32	2.24	1.13	-	-	-
0.11	8.35	7.39	6.41	5.41	4.38	3.32	2.24	1.13	-	-

	0.03	0.04	0.05	0.06	0.07	0.08	0.09	0.1	0.11	0.12
0.12	9.30	8.36	7.40	6.42	5.41	4.38	3.33	2.25	1.14	-
0.13	10.23	9.31	8.37	7.41	6.43	5.42	4.39	3.33	2.25	1.14
0.14	11.14	10.24	9.32	8.38	7.42	6.44	5.43	4.39	3.34	2.25
0.15	12.04	11.16	10.26	9.34	8.40	7.43	6.45	5.44	4.40	3.34
0.16	12.91	12.05	11.17	10.27	9.35	8.41	7.44	6.45	5.44	4.41
0.17	13.78	12.93	12.07	11.19	10.28	9.36	8.42	7.45	6.46	5.45
0.18	14.62	13.79	12.95	12.08	11.20	10.30	9.37	8.43	7.46	6.47
0.19	15.45	14.64	13.81	12.96	12.10	11.22	10.31	9.39	8.44	7.47
0.2	16.26	15.47	14.66	13.83	12.98	12.12	11.23	10.33	9.40	8.45

Table 8 shows the total sample size needed to achieve 80% power to detect a statistically significant (with alpha=0.05) difference in the 3-year event rates presented in Table 5 between the intervention group and the control group, for different combinations of AF proportions.

Table 8: Total sample size needed for different combinations of AF proportions in the intervention and control group, respectively. Columns: AF proportions in the control group. Rows: AF proportions in the intervention group.

	0.03	0.04	0.05	0.06	0.07	0.08	0.09	0.1	0.11	0.12
0.1	13380	18096	25892	40198	71006	158734	630838	-	-	-
0.11	10296	13362	18072	25858	40144	70910	158518	629976	-	-
0.12	8176	10282	13346	18048	25824	40090	70814	158302	629114	-
0.13	6656	8166	10270	13328	18024	25790	40036	70718	158088	628250
0.14	5530	6648	8156	10256	13310	18000	25754	39982	70622	157872
0.15	4670	5522	6640	8144	10242	13292	17976	25720	39928	70526
0.16	3998	4664	5514	6630	8134	10228	13274	17952	25686	39874
0.17	3466	3994	4658	5508	6622	8124	10216	13258	17928	25652
0.18	3034	3460	3988	4652	5500	6614	8112	10202	13240	17904
0.19	2680	3030	3456	3984	4646	5494	6604	8102	10188	13222
0.2	2386	2676	3026	3452	3978	4640	5486	6596	8092	10174

Selected sample size

To calculate the expected cases in the intervention group and the control group, the function *cpower* was run with the following parameters:

- accrual time 2 years;
- follow-up time 3 years;
- aplha 0.05;
- power 0.8;
- 5% yearly rate of stroke/death/intracranial bleeding for AF-/OAK-;
- 19% AF proportions in the intervention group;
- 6% AF proportions in the control group.

The result of *cpower* is:

```
Accrual duration: 2 y  Minimum follow-up: 3 y
```

Biostatistics
CORE FACILITY

```
Total sample size: 2718

Alpha= 0.05

3-year Mortalities
     Control Intervention
   0.1901558    0.1544025

Hazard Rates
     Control Intervention
  0.07030447   0.05590393

Probabilities of an Event During Study
     Control Intervention
   0.2445141    0.1999611

Expected Number of Events
     Control Intervention
       332.3        271.7

Hazard ratio: 0.7951689
Standard deviation of log hazard ratio: 0.08178795
    Power
0.8002227
```

In the first two years, you have to enroll uniformly over time 1359 patients in the control group and 1359 in the intervention group. After the first two years, you have to follow them up for another three years. At least five years after the start of the study, you must check the observed cases in the control group and in the intervention group. If the study did not reach 333 cases in the control group and 272 in the intervention group, the follow-up can be extended until these are reached. This minimum number of cases guarantees a power of 80%.

Patient consent form

Patientnummer:________________

Bilaga 05

Randomiserad jämförelse mellan standardiserad och utökad EKG-undersökning efter stroke eller TIA för att detektera förmaksflimmer och minska risken för stroke - AF SPICE

Forskningspersonsformation och samtyckesformulär

För närvarande pågår i Sverige en vetenskaplig studie som går ut på att studera två olika metoder att upptäcka förmaksflimmer hos personer som drabbats av slaganfall (stroke) eller TIA (övergående stroke). Det är orsaken till att du får denna information och förfrågan. Studien drivs av Karolinska Institutet.

Varför görs detta forskningsprojekt?

Förmaksflimmer är den vanligaste rubbningen i hjärtrytmen och förekomsten ökar med stigande ålder. Tillståndet ger oregelbunden hjärtrytm och symptomen varierar kraftigt, i vissa fall kan man ha förmaksflimmer utan några symptom alls.

Vid förmaksflimmer finns en ökad risk att drabbas av stroke, detta beror på en ökad risk för att det bildas blodproppar i hjärtats förmak, dessa proppar kan sedan följa med blodet till hjärnan och därmed orsaka stroke eller TIA. Risken för stroke vid förmaksflimmer kan reduceras med 60-70% med blodförtunnande behandling. Blodförtunnande behandling innebär också en viss ökning av risken att drabbas av allvarlig blödning.

För de som drabbats av stroke eller TIA är det angeläget att ta reda på om individen har förmaksflimmer. Om så är fallet finns det starka skäl att starta en särskild sorts blodförtunnande behandling, så kallad *antikoagulantia*. Utredning för att hitta förmaksflimmer efter stroke eller TIA görs med olika typer av EKG-undersökningar, ofta över en eller flera dygn. Det finns ännu inte någon forskning som visar på vilken typ av EKG-undersökning som är den bästa efter stroke, vilket också har lett till att praxis varierar. Långa EKG-undersökningar tar också stora resurser i anspråk varför det är angeläget att ta reda på den medicinska nyttan med olika metoder.

I detta forskningsprojekt kommer du att lottas till ett av två alternativ: standardutredning eller utökad utredning. Standardutredningen består av 24-48 timmars EKG-utredning och är den utredning som rekommenderas starkast av Socialstyrelsen. Utökad utredning består av långtids-EKG med så kallat plåster-EKG under 14 dagar vid två tillfällen.

Studien är godkänd av Etikprövningsmyndigheten.

Vad innebär det för mig att delta i studien?

För dig som lottas till **standardutredning** kommer EKG-undersökning genomföras inneliggande på avdelning eller som långtids-EKG med portabel utrustning i hemmet.

För dig som lottas till **utökad utredning** kommer EKG-undersökning med plåster-EKG starta på avdelningen och oftast avslutas efter utskrivning då du sänder tillbaka EKG-plåstret med

Patientnummer:________________

Bilaga 05

Randomiserad jämförelse mellan standardiserad och utökad EKG-undersökning efter stroke eller TIA för att detektera förmaksflimmer och minska risken för stroke - AF SPICE

ett medsänt svarskuvert. Du kommer också att i samband med ett återbesök påbörja ytterligare en 14-dagars EKG-registrering med plåster-EKG där du sänder tillbaka utrustningen på samma sätt.

Om EKG i någon av undersökningarna visar på förmaksflimmer kommer du utan fördröjning att kontaktas av läkare för att diskutera eventuell ändring av din blodförtunnande behandling.

Därefter görs en långtidsuppföljning under minst tre år där Socialstyrelsens register samt registret Riks-stroke kommer att studeras för att bland annat följa förekomsten av insjuknanden i stroke, förskrivning av proppförebyggande läkemedel och överlevnad. Denna uppföljning kräver ingen aktiv medverkan från dig. De register från Socialstyrelsen som kommer att användas är patientregistret, läkemedelsregistret och dödsorsaksregistret.

Ersättning för deltagande och resor utgår inte.

Vilka obehag/risker medför studien?

Både förmaksflimmer och blodförtunnande behandling gör att man är utsatt för vissa risker, men den riskminskning som åstadkommes med blodförtunnande behandling är i de allra flesta fall flerfalt större än den risk som behandlingen innebär. Denna behandling rekommenderas också i Socialstyrelsens senaste nationella riktlinjer för hjärtsjukvård.

Undersökning med långtids-EKG och plåster-EKG kan i vissa fall ge hudirritation där utrustningen ansluts på huden. Dessa besvär är ofta lindriga och alltid övergående.

Vilka fördelar kan studien innebära för dig?

Ditt deltagande ger en möjlighet att upptäcka och behandla ett tillstånd som ökar risken för ny stroke och TIA, dessutom kan studien ge oss värdefulla kunskaper som även andra kan ha nytta av i framtiden

Hur insamlas personuppgifter och hur hanteras sekretess?

Dina svar och dina resultat kommer att behandlas så att inte obehöriga kan ta del av dem. Ansvarig för dina personuppgifter är Region Stockholm. Enligt EU:s dataskyddsförordning (GDPR 2016/679) har du rätt att kostnadsfritt få ta del av de uppgifter om dig som hanteras i studien, och vid behov få eventuella fel rättade. Du kan också begära att uppgifter om dig raderas samt att behandlingen av dina personuppgifter begränsas. Om du vill ta del av uppgifterna ska du kontakta personuppgiftsombudet på dso.ds@sll.se [alt mailadress för aktuellt site] eller ansvarig forskare. Om du är missnöjd med hur dina personuppgifter behandlas har du rätt att ge in klagomål till Integritetsskyddsmyndigheten, som är tillsynsmyndighet.

Engdahl J, *et al. BMJ Open* 2023; 13:e073470. doi: 10.1136/bmjopen-2023-073470

Patientnummer:__________________

Bilaga 05

Randomiserad jämförelse mellan standardiserad och utökad EKG-undersökning efter stroke eller TIA för att detektera förmaksflimmer och minska risken för stroke - AF SPICE

Är deltagandet i studien frivilligt?
Deltagandet i denna studie är frivilligt, kostnadsfritt och du har när som helst rätt att avbryta ditt deltagande utan att ange skäl och utan att det påverkar ditt framtida omhändertagande. Kostnad för eventuell läkemedelsbehandling efter ditt deltagande omfattas av sedvanlig läkemedelsförmån.

Kontaktpersoner

Om du vill ha ytterligare information om studien, dina rättigheter som patient eller om problem uppstår under studien ska du vända dig till:

Ansvarig prövare på stroke-avdelningen: XXXX XXXX

För studien ansvarig läkare:

Johan Engdahl, Överläkare/Docent
Karolinska Institutet, Danderyds Sjukhus
Hjärtkliniken
Danderyds Sjukhus
182 57 Danderyd
Johan.engdahl@sll.se

Patientnummer:__________________

Bilaga 05

Randomiserad jämförelse mellan standardiserad och utökad EKG-undersökning efter stroke eller TIA för att detektera förmaksflimmer och minska risken för stroke - AF SPICE

Samtycke till att delta i studien

Jag har fått muntlig och skriftlig informationen om studien och har haft möjlighet att ställa frågor. Jag får behålla den skriftliga informationen.

☐ Jag samtycker till att delta i studien *Randomiserad jämförelse mellan standardiserad och utökad EKG-undersökning efter stroke eller TIA för att detektera förmaksflimmer och minska risken för stroke - AF SPICE*

☐ Jag samtycker till att uppgifter om mig behandlas på det sätt som beskrivs i forskningspersonsinformationen.

☐ Jag samtycker till att uppgifter om mig ur de olika register som beskrivs ovan utförs som långtidsuppföljning

Ort Datum ________________________

____
Forskningspersons namnteckning

____
Namnförtydligande

Jag har förklarat studiens upplägg och syfte, samt inhämtat samtycke för deltagande:

Ort Datum ________________________

____
Namnteckning prövare

____
Namnförtydligande

Engdahl J, *et al. BMJ Open* 2023; 13:e073470. doi: 10.1136/bmjopen-2023-073470

References

1. Diener HC, Connolly SJ, Ezekowitz MD, Wallentin L, Reilly PA, Yang S, Xavier D, Di Pasquale G and Yusuf S. Dabigatran compared with warfarin in patients with atrial fibrillation and previous transient ischaemic attack or stroke: a subgroup analysis of the RE-LY trial. *Lancet Neurol*. 2010;9:1157-63.

2. Diener HC, Eikelboom J, Connolly SJ, Joyner CD, Hart RG, Lip GY, O'Donnell M, Hohnloser SH, Hankey GJ, Shestakovska O, Yusuf S, Committee AS and Investigators. Apixaban versus aspirin in patients with atrial fibrillation and previous stroke or transient ischaemic attack: a predefined subgroup analysis from AVERROES, a randomised trial. *Lancet Neurol*. 2012;11:225-31.

3. Amarenco P, Lavallée PC, Labreuche J, Albers GW, Bornstein NM, Canhão P, Caplan LR, Donnan GA, Ferro JM, Hennerici MG, Molina C, Rothwell PM, Sissani L, Školoudík D, Steg PG, Touboul PJ, Uchiyama S, Vicaut É and Wong LK. One-Year Risk of Stroke after Transient Ischemic Attack or Minor Stroke. *N Engl J Med*. 2016;374:1533-42.

4. Andersen SD, Gorst-Rasmussen A, Lip GY, Bach FW and Larsen TB. Recurrent Stroke: The Value of the CHA2DS2VASc Score and the Essen Stroke Risk Score in a Nationwide Stroke Cohort. *Stroke*. 2015;46:2491-7.

5. Wachter R, Groschel K, Gelbrich G, Hamann GF, Kermer P, Liman J, Seegers J, Wasser K, Schulte A, Jurries F, Messerschmid A, Behnke N, Groschel S, Uphaus T, Grings A, Ibis T, Klimpe S, Wagner-Heck M, Arnold M, Protsenko E, Heuschmann PU, Conen D, Weber-Kruger M, Find AFI and Coordinators. Holter-electrocardiogram-monitoring in patients with acute ischaemic stroke (Find-AFRANDOMISED): an open-label randomised controlled trial. *Lancet Neurol*. 2017;16:282-290.

6. Hankey GJ, Patel MR, Stevens SR, Becker RC, Breithardt G, Carolei A, Diener HC, Donnan GA, Halperin JL, Mahaffey KW, Mas JL, Massaro A, Norrving B, Nessel CC, Paolini JF, Roine RO, Singer DE, Wong L, Califf RM, Fox KA, Hacke W and Investigators RASC. Rivaroxaban compared with warfarin in patients with atrial fibrillation and previous stroke or transient ischaemic attack: a subgroup analysis of ROCKET AF. *Lancet Neurol*. 2012;11:315-22.

7. Easton JD, Lopes RD, Bahit MC, Wojdyla DM, Granger CB, Wallentin L, Alings M, Goto S, Lewis BS, Rosenqvist M, Hanna M, Mohan P, Alexander JH, Diener HC, Committees A and Investigators. Apixaban compared with warfarin in patients with atrial fibrillation and previous stroke or transient ischaemic attack: a subgroup analysis of the ARISTOTLE trial. *Lancet Neurol*. 2012;11:503-11.

8. Rost NS, Giugliano RP, Ruff CT, Murphy SA, Crompton AE, Norden AD, Silverman S, Singhal AB, Nicolau JC, SomaRaju B, Mercuri MF, Antman EM, Braunwald E and Investigators EA-T. Outcomes With Edoxaban Versus Warfarin in Patients With Previous Cerebrovascular Events: Findings From ENGAGE AF-TIMI 48 (Effective Anticoagulation With Factor Xa Next Generation in Atrial Fibrillation-Thrombolysis in Myocardial Infarction 48). *Stroke*. 2016;47:2075-82.

9. Lip GY, Hunter TD, Quiroz ME, Ziegler PD and Turakhia MP. Atrial Fibrillation Diagnosis Timing, Ambulatory ECG Monitoring Utilization, and Risk of Recurrent Stroke. *Circ Cardiovasc Qual Outcomes*. 2017;10.

10. Jabaudon D, Sztajzel J, Sievert K, Landis T and Sztajzel R. Usefulness of ambulatory 7-day ECG monitoring for the detection of atrial fibrillation and flutter after acute stroke and transient ischemic attack. *Stroke*. 2004;35:1647-51.

11. Shafqat S, Kelly PJ and Furie KL. Holter monitoring in the diagnosis of stroke mechanism. *Intern Med J*. 2004;34:305-9.

12. Alhadramy O, Jeerakathil TJ, Majumdar SR, Najjar E, Choy J and Saqqur M. Prevalence and predictors of paroxysmal atrial fibrillation on Holter monitor in patients with stroke or transient ischemic attack. *Stroke*. 2010;41:2596-600.

13. Rizos T, Rasch C, Jenetzky E, Hametner C, Kathoefer S, Reinhardt R, Hepp T, Hacke W and Veltkamp R. Detection of paroxysmal atrial fibrillation in acute stroke patients. *Cerebrovasc Dis*. 2010;30:410-7.

14. Doliwa Sobocinski P, Anggardh Rooth E, Frykman Kull V, von Arbin M, Wallen H and Rosenqvist M. Improved screening for silent atrial fibrillation after ischaemic stroke. *Europace*. 2012;14:1112-6.

15. Lazzaro MA, Krishnan K and Prabhakaran S. Detection of atrial fibrillation with concurrent holter monitoring and continuous cardiac telemetry following ischemic stroke and transient ischemic attack. *J Stroke Cerebrovasc Dis*. 2012;21:89-93.

16. Gumbinger C, Krumsdorf U, Veltkamp R, Hacke W and Ringleb P. Continuous monitoring versus HOLTER ECG for detection of atrial fibrillation in patients with stroke. *Eur J Neurol*. 2012;19:253-7.

17. Suissa L, Lachaud S and Mahagne MH. Optimal Timing and Duration of Continuous Electrocardiographic Monitoring for Detecting Atrial Fibrillation in Stroke Patients. *J Stroke Cerebrovasc Dis*. 2012.

18. Thakkar S and Bagarhatta R. Detection of paroxysmal atrial fibrillation or flutter in patients with acute ischemic stroke or transient ischemic attack by Holter monitoring. *Indian Heart J*. 2014;66:188-92.

19. Bansil S and Karim H. Detection of atrial fibrillation in patients with acute stroke. *J Stroke Cerebrovasc Dis*. 2004;13:12-5.

20. Barthelemy JC, Feasson-Gerard S, Garnier P, Gaspoz JM, Da Costa A, Michel D and Roche F. Automatic cardiac event recorders reveal paroxysmal atrial fibrillation after unexplained strokes or transient ischemic attacks. *Ann Noninvasive Electrocardiol*. 2003;8:194-9.

21. Wallmann D, Tuller D, Wustmann K, Meier P, Isenegger J, Arnold M, Mattle HP and Delacretaz E. Frequent atrial premature beats predict paroxysmal atrial fibrillation in stroke patients: an opportunity for a new diagnostic strategy. *Stroke*. 2007;38:2292-4.

22. Tayal AH, Tian M, Kelly KM, Jones SC, Wright DG, Singh D, Jarouse J, Brillman J, Murali S and Gupta R. Atrial fibrillation detected by mobile cardiac outpatient telemetry in cryptogenic TIA or stroke. *Neurology*. 2008;71:1696-701.

23. Elijovich L, Josephson SA, Fung GL and Smith WS. Intermittent atrial fibrillation may account for a large proportion of otherwise cryptogenic stroke: a study of 30-day cardiac event monitors. *J Stroke Cerebrovasc Dis*. 2009;18:185-9.

24. Bhatt A, Majid A, Razak A, Kassab M, Hussain S and Safdar A. Predictors of occult paroxysmal atrial fibrillation in cryptogenic strokes detected by long-term noninvasive cardiac monitoring. *Stroke Res Treat*. 2011;2011:172074.

25. Flint AC, Banki NM, Ren X, Rao VA and Go AS. Detection of paroxysmal atrial fibrillation by 30-day event monitoring in cryptogenic ischemic stroke: the Stroke and Monitoring for PAF in Real Time (SMART) Registry. *Stroke*. 2012;43:2788-90.

26. Higgins P, MacFarlane PW, Dawson J, McInnes GT, Langhorne P and Lees KR. Noninvasive cardiac event monitoring to detect atrial fibrillation after ischemic stroke: a randomized, controlled trial. *Stroke*. 2013;44:2525-31.

27. Miller DJ, Khan MA, Schultz LR, Simpson JR, Katramados AM, Russman AN and Mitsias PD. Outpatient cardiac telemetry detects a high rate of atrial fibrillation in cryptogenic stroke. *J Neurol Sci*. 2013;324:57-61.

28. Gladstone DJ, Spring M, Dorian P, Panzov V, Thorpe KE, Hall J, Vaid H, O'Donnell M, Laupacis A, Cote R, Sharma M, Blakely JA, Shuaib A, Hachinski V, Coutts SB, Sahlas DJ, Teal P, Yip S, Spence JD, Buck B, Verreault S, Casaubon LK, Penn A, Selchen D, Jin A, Howse D, Mehdiratta M, Boyle K, Aviv R, Kapral MK and Mamdani M. Atrial fibrillation in patients with cryptogenic stroke. *N Engl J Med*. 2014;370:2467-77.

29. Sebasigari D, Merkler A, Guo Y, Gialdini G, Kummer B, Hemendinger M, Song C, Chu A, Cutting S, Silver B, Elkind MSV, Kamel H, Furie KL and Yaghi S. Biomarkers of Atrial Cardiopathy and Atrial Fibrillation Detection on Mobile Outpatient Continuous Telemetry After Embolic Stroke of Undetermined Source. *J Stroke Cerebrovasc Dis*. 2017;26:1249-1253.

30. Kaura A, Sztriha L, Chan FK, Aeron-Thomas J, Gall N, Piechowski-Jozwiak B, Teo JT. Early prolonged ambulatory cardiac monitoring in stroke (EPACS): an open-label randomised controlled trial. *Eur J Med Res.* 2019;24(1):25.
