# Supplementary material for: Multicentre, national, investigator-initiated, randomised, parallel-group, register-based superiority trial to compare extended ECG monitoring versus standard ECG monitoring in elderly patients with ischaemic stroke or transient ischaemic attack and the effect on stroke, death and intracerebral bleeding: the AF SPICE protocol
Source: BMJ Open. 2023 Nov 23;13(11):e073470. doi: 10.1136/bmjopen-2023-073470 (PMC10668286; doi:10.1136/bmjopen-2023-073470)
Supplement: Author's manuscript [file bmjopen-2023-073470.draft_revisions.pdf]

BMJ Open

BMJ Open is committed to open peer review. As part of this commitment we make the peer review history of every article we publish publicly available.

When an article is published we post the peer reviewers' comments and the authors' responses online. We also post the versions of the paper that were used during peer review. These are the versions that the peer review comments apply to.

The versions of the paper that follow are the versions that were submitted during the peer review process. They are not the versions of record or the final published versions. They should not be cited or distributed as the published version of this manuscript.

BMJ Open is an open access journal and the full, final, typeset and author-corrected version of record of the manuscript is available on our site with no access controls, subscription charges or pay-per-view fees (http://bmjopen.bmj.com).

If you have any questions on BMJ Open's open peer review process please email info.bmjopen@bmj.com

BMJ Open

A multicenter, national, investigator-initiated, randomised, parallel-group, register based superiority trial to compare extended ECG monitoring versus standard ECG monitoring in elderly patients with ischemic stroke or transient ischemic attack and the effect on stroke, death and intracerebral bleeding – AF SPICE.

Journal:	*BMJ Open*
Manuscript ID	bmjopen-2023-073470
Article Type:	Protocol
Date Submitted by the Author:	06-Mar-2023

Complete List of Authors:	Engdahl, Johan; Karolinska Institutet, Department of Clinical Sciences, Danderyd Hospital<br>Straat, Kajsa; Karolinska Institutet, Department of clinical sciences, Danderyd Hospital<br>Isaksson, Eva; Karolinska Institutet, Department of clinical sciences, Danderyd Hospital<br>Rooth, Elisabeth; Karolinska Institutet, Department of clinical Sciences, Danderyd Hospital<br>Svennberg, Emma; Karolinska Institutet, Department of Medicine, Huddinge, Karolinska University Hospital,<br>Norrving, Bo; Lund University, Section of Neurology, Department of Clinical Sciences<br>Euler, Mia; Orebro universitet, School of Medicine, Department of Neurology<br>Hellqvist, Kjersti; Alingsas lasarett, Department of Medicine<br>Gu, Weigang; Karolinska Institutet, Department of Clinical Sciences, South Hospital<br>Ström, Jakob; Orebro universitet, School of Medicine, Department of Neurology<br>Själander, Sara; Umeå University, Department of Public Health and Clinical Medicine<br>Eriksson, Marie; Umeå University, Department of Statistics, USBE<br>Åsberg, Signild; Uppsala University, Department of Medical Sciences<br>Wester, Per; Umeå University, Department of Public Health and Clinical Medicine; Karolinska Institutet, Dept of Clinical Sciences, Danderyd Hospital

Keywords:	Stroke < NEUROLOGY, Cardiology < INTERNAL MEDICINE, Thromboembolism < CARDIOLOGY

SCHOLARONE™
Manuscripts

A multicenter, national, investigator-initiated, randomised, parallel-group, register based superiority trial to compare extended ECG monitoring versus standard ECG monitoring in elderly patients with ischemic stroke or transient ischemic attack and the effect on stroke, death and intracerebral bleeding – AF SPICE.

Johan Engdahl[1], Kajsa Strååt[1], Eva Isaksson[1], Elisabeth Rooth[1], Emma Svennberg[2], Bo Norrving[3], Mia von Euler[4], Kjersti Hellqvist[5], Weigang Gu[6], Jakob O Ström[4], Sara Själander[7], Marie Eriksson[8], Signild Åsberg[9]*, Per Wester[1,7]*

*Shared senior authorship

1. Karolinska Institutet, Dept of Clinical Sciences, Danderyd Hospital, Stockholm, Sweden.

2. Karolinska Institutet, Dept of Medicine, Huddinge, Karolinska University Hospital, Stockholm, Sweden.

3. Section of Neurology, Department of Clinical Sciences, Lund University, Lund, Sweden.

4. School of Medicine, Department of Neurology, Örebro University, Örebro, Sweden

5. Department of Medicine, Alingsås Hospital, Alingsås, Sweden.

6. Karolinska Institutet, Dept of Clinical Sciences, South Hospital, Stockholm, Sweden.

7. Department of Public Health and Clinical Medicine, Umeå University, Umeå, Sweden.

8. Dept of Statistics, USBE, Umeå University, Umeå, Sweden

9. Dept of Medical Sciences, Uppsala University, Uppsala, Sweden

Corresponding author:

Johan Engdahl

Department of Cardiology

Danderyd University Hospital

SE-182 88 Stockholm

Sweden

For peer review only

29 Email: johan.engdahl@regionstockholm.se

30 Phone: +46 737 593374

31 Fax: +46 8 7550868

32 Word count: main 3996; abstract 259

For peer review only

For peer review only - http://bmjopen.bmj.com/site/about/guidelines.xhtml

ABSTRACT

Introduction

Atrial fibrillation (AF) is a major risk factor for ischemic stroke and transient ischemic attack (TIA), and AF detection can be challenged by asymptomatic and paroxysmal presentation. Long-term ECG after ischemic stroke or TIA is recommended by all major societies in cardiology and cerebrovascular medicine. However, data on stroke reduction are lacking, and the recommendations show significant diversity.

Methods and analysis

AF SPICE is a multicenter, national, investigator-initiated, randomised, parallel-group, register-based trial comparing extended ECG monitoring versus standard ECG monitoring in patients submitted for ischemic stroke or TIA, with a composite endpoint of stroke, all-cause-mortality and intracerebral bleeding. Patients aged ≥70 years, without previous AF will be randomised 1:1 to control (standard ECG monitoring) or intervention (extended ECG monitoring). In the control arm, patients will undergo 48h±24h of continuous ECG monitoring according to national recommendations. In the intervention arm, patients will undergo 14+14 days of continuous ECG monitoring three months apart using an ECG patch device. All ECG patch recordings will be read in a core facility. In case of AF detection, oral anticoagulation will be recommended if not contraindicated. A pilot phase has been concluded in 2022 which will transcend into the main trial during 2023-2026, including approximately 30 stroke units. The sample size is calculated to 3,262 patients. The primary outcome will be collected from register data during a 36-month follow-up.

For peer review only - http://bmjopen.bmj.com/site/about/guidelines.xhtml

Ethics and dissemination

Ethical approval has been provided by the Swedish Ethical Review Authority, reference 2021-02770. The trial will be conducted according to the ethical principles of the declaration of Helsinki and national regulatory standards.

Trial Registration

ClinicalTrials.gov NCT05134454

Trial protocol version 2.0

Sponsor contact information:

Karolinska Institutet

Department of clinical sciences

Danderyd Hospital

SE-18288 Danderyd

Sweden

For peer review only

For peer review only - http://bmjopen.bmj.com/site/about/guidelines.xhtml

STRENGTH AND LIMITATIONS OF THE TRIAL

- First register-based randomised clinical trial on ECG screening post stroke/TIA with stroke and all-cause mortality endpoints
- Participants included from a national network of stroke units and central adjudication of ECG recordings in the intervention arm
- Robust inclusion criteria mirroring clinical real-world population, age at least 70 years for inclusion
- The Swedish stroke register (Riksstroke) used for provision of majority of baseline data
- Endpoints from health care registers will not be further adjudicated

For peer review only

For peer review only - http://bmjopen.bmj.com/site/about/guidelines.xhtml

INTRODUCTION

Ischemic stroke is one of the leading causes of mortality worldwide, and a major cause behind permanent disability in adults[1]. Despite implementation of novel treatment strategies during the latest decade, morbidity and mortality remain significant.

Modifiable risk factors for stroke include hypertension, atrial fibrillation (AF), hyperlipidaemia, smoking and diabetes[2, 3]. Several of these risk factors for stroke have the potential of being undetected for a long time since they may remain asymptomatic.

During 2018, the stroke incidence in Sweden was 223 per 100 000 in men and 191 per 100 000 in women[4]. At three-month follow up post-stroke, 26% were deceased or dependent in activities of daily living[5]. Stroke is predominantly a disease of the elderly with 83% being 65 years or older[4].

In addition to human suffering, the societal burden of the disease is significant. Disability with need for long-term care and in younger patients impaired work ability entails enormous societal costs[6]. Strokes due to AF are more severe and have a significantly higher mortality than other stroke cases[7].

AF is the most prevalent clinically arrhythmia with a steeply increasing incidence with advancing age[8]. AF is also one of the strongest risk factors for stroke[3]. However, the increased stroke risk associated with AF can be markedly reduced by oral anticoagulation (OAC) treatment[9]. Unfortunately, AF is paroxysmal and asymptomatic in a significant proportion of stroke patients, leading to lower detection rates, and frequently leave stroke survivors with an untreated risk factor and higher risk of stroke recurrence[10]. Despite this, no

For peer review only

For peer review only - http://bmjopen.bmj.com/site/about/guidelines.xhtml

study so far has reported evidence to the benefit of electrocardiographic (ECG) AF screening in terms of reduced stroke recurrence and mortality after a stroke event.

A German multicenter study comparing 72-hour Holter with standard 24-hour workup showed an increase in AF detection from 2.9% to 4.3%[11].

Randomised controlled trials (CRYSTAL-AF[12] and EMBRACE[10]) have shown that prolonged ECG-monitoring (insertable cardiac monitor and 30-day event-triggered loop recorder respectively) compared to standard of care (at the time 24 hr-ECG) increased detection of atrial fibrillation in cryptogenic stroke patients.

The FIND-$AF_{randomised}$ trial[13] also showed increased detection of atrial fibrillation and included a broader group of participants suggesting that prolonged ECG-monitoring should be used in all ischemic stroke patients if detection of atrial fibrillation would lead to anticoagulation therapy.

The MonDAfis study[14] was powered to evaluate the proportion of anticoagulation therapy 12 months after index stroke. Standard of care was compared to prolonged in hospital ECG-monitoring (maximum 7 days). Atrial fibrillation detection was increased in the intervention group, but this had no significant effect on the rate of anticoagulation therapy at 12 months.

AF screening post an ischemic stroke event is currently recommended by all major international societies (Table 1)[15-21]. The recommendations vary markedly with respect to method of screening and duration of screening, which probably reflects the lack of trials with stroke and mortality as endpoints. There are however a body of data on the proportion of AF detection using different monitoring strategies[22].

For peer review only - http://bmjopen.bmj.com/site/about/guidelines.xhtml

The development of ECG modalities and clinical practice for AF screening post ischemic stroke and TIA have evolved towards longer and more resource-demanding investigations as noted in table 1, despite the lack of data on clinical endpoints like stroke. Diversity within recommendations have promoted unequal care even within countries and regions. With this background, there is an urgent need for a randomised trial to investigate the efficacy of AF screening following ischemic stroke.

Choice of comparators

In some of the recommendations on AF-screening post-ischemic stroke from international societies, a basic monitoring duration of at least 24 hours of continuous ECG is recommended[16, 18]. In the recommendations from the Swedish Board of Health and Welfare, continuous ECG monitoring of 24-48 hours is recommended as the baseline investigation for most ischemic stroke patients, and a large share of stroke units in Sweden adhere to this recommendation. Its selection as comparator is therefore justified.

METHODS AND ANALYSIS

Primary Objective

To determine if extended continuous ECG screening (14+14 days) is superior to standard ECG screening (48 ±24 hours) in reducing the risk of the combined endpoint of ischemic stroke, intracerebral bleeding and all-cause mortality in elderly patients admitted to hospital for ischemic stroke or TIA.

For peer review only

For peer review only - http://bmjopen.bmj.com/site/about/guidelines.xhtml

Key Secondary Objectives

The key secondary objectives are to estimate the treatment effect in primary outcome, and determine if there are any differences between extended and standard ECG screening for:

- Ischemic stroke
- All-cause mortality
- Intracranial bleeding
- Major bleeding
- Myocardial infarction
- Pacemaker implantation
- Cost effectiveness

Other Secondary Objectives

- To study association between age and AF detection
- To study the proportion of other relevant arrhythmias detected at ECG screening
- To study predictors for AF detection
- To study signal time and signal quality achieved at ECG screening
- To study long-term adherence to OAC treatment
- To study feasibility of patients or their next-of-kin application of the second ECG patch
- To study Patient Reported Experience (PREM), collected 3 months after inclusion in the main trial
- Per protocol analysis of the primary outcome
- Win ratio analysis

For peer review only

For peer review only - http://bmjopen.bmj.com/site/about/guidelines.xhtml

Trial design (Figure 1)

AF SPICE is designed as a multicenter, national, investigator-initiated, randomised, parallel-group, register based superiority trial to compare extended ECG monitoring versus standard ECG Monitoring in Elderly Patients submitted for ischemic stroke or TIA with the combined endpoint of recurrent ischemic stroke, all-cause mortality and intracerebral bleeding.

Study setting

AF SPICE will recruit participants among admissions for ischemic stroke or TIA in stroke units in Swedish hospitals. Inclusion is also possible from non-stroke units given that the patient is admitted for ischemic stroke or TIA and identified by the site's local investigator or delegate.

A pilot trial commenced in January 2022 and is now concluded, converting into the main study in 2023. The pilot trial evaluated inclusion rates, feasibility and adherence to ECG recorders and the database with randomisation system.

Eligibility criteria

Inclusion criteria

1. Patients aged ≥ 70 years admitted with a diagnosis of ischemic stroke or TIA within 14 days from inclusion.
2. Signed informed consent.

Exclusion criteria

1. Previously diagnosed AF

For peer review only - http://bmjopen.bmj.com/site/about/guidelines.xhtml

2. Contraindication to oral anticoagulant treatment according to summary of product characteristics
3. Indication for anticoagulant treatment other than AF, e.g. venous thromboembolism or mechanical heart valve prosthesis
4. Patients with pacemaker, implantable cardioverter defibrillator or implantable loop recorder.
5. Patients who, according to the investigator, will not be able to comply with the trial protocol.
6. Previous participation in the AF SPICE trial

Withdrawal of patients

A patient can withdraw from the trial at any time, at the discretion of the patient, or by the investigator if deemed medically indicated. If a participant refrain from completing extended ECG recording protocol, he/she will remain included in the trial and in the intention-to-screen data analysis. A withdrawal of consent means that no further trial data will be collected, however already collected data will not be erased.

Patient and public involvement

There was no patient or public involvement in this trial.

For peer review only

Interventions

Extended ECG Investigation

Participants randomised to extended ECG investigation will undergo a continuous one-lead ECG-recording using the BioTel ePatch (https://www.biotel.se). The BioTel ePatch is a class IIa CE-marked Holter ECG recorder using a patch for skin adhesion.

The ECG patch will be applied by health care professionals at the stroke unit (visit 1) according to the manufacturer's recommendation at randomisation.

After completion of the 14-day ECG recording, the device should be returned using a pre-paid envelope. Following arrival at BioTel, the ECG data will be uploaded to a web-accessed interpretation service (Cardiologs, https://cardiologs.com/) within 1 working day from arrival.

During visit 2, participants not diagnosed with AF will have the second ePatch attached by health care professionals in connection to a scheduled follow-up visit or attached by the participant/next of kin.

The ECG recordings will be scrutinised by a core ECG reading team led by JE.

Procedures for handling of arrhythmias detected on extended ECG recording

When participants are diagnosed with a new AF or atrial flutter according to the current definition of the ESC AF guidelines[17], the local investigator will be notified within one working day by phone and email by the ECG reading team.

For peer review only - http://bmjopen.bmj.com/site/about/guidelines.xhtml

If a participant is diagnosed with other arrhythmias with a prognostic implication as defined in table 2, the local investigator will be notified by email within three working days by the ECG reading team. If a more rapid action is deemed necessary by the ECG reading team, appropriate measures such as contacting the local investigator or delegate by phone will be undertaken.

Procedures for poor signal quality and short recordings in extended ECG

If the signal quality of the extended ECG recording is deemed to be of insufficient quality, the participant should be offered another attempt of patch ECG recording if possible. If this second recording have insufficient signal quality or if it is not possible to arrange for another patch ECG recording, a traditional Holter recording, or ECG event recording could be used as a substitute. However, it is of importance for the power of the trial to achieve as long ECG recordings as possible in the intervention group. Hence, in a situation where patch ECG is not possible to perform for whatever reason, a continuous event loop recording should be the first option, preferably with a duration of 14 days.

At least 7 days of ECG recording should be yielded from ECG patch in connection with visit one. If less than 7 days are recorded, another recording should be offered the participant. The same minimum ECG duration of 7 days applies for ECG recording at visit two.

If a participant randomised to intervention arm with a negative ECG recording (i.e., no atrial fibrillation detected) suffers a recurrent ischemic stroke within 2 months from visit

For peer review only

For peer review only - http://bmjopen.bmj.com/site/about/guidelines.xhtml

one, the second ECG patch recording (visit 2) may be started earlier than 2 months to the discretion of the local investigator.

Oral anticoagulation initiation following AF detection in extended ECG recording

Patients newly diagnosed with AF during the long-term ECG recordings initiated in connection to randomisation should be informed by the local investigator or delegate and offered treatment with oral anticoagulation if there are no contraindications, based on the stroke risk of at least CHA_2DS_2-VASc 3 points in randomised patients.

Treatment with OAC and its long-term follow-up will be handled within standard care and according to national and international guidelines.

When indicated, OAC treatment should be initiated within five working days from the reception of long-term ECG results. Long-term follow-up of OAC treatment will be using the national drug prescription registry.

All other treatments and investigations will be at the discretion of the local investigator.

Standard care

Participants randomised to standard care should undergo 48 ±24 hours of continuous ECG monitoring, using inpatient ECG telemetry monitoring or Holter recording. The duration of the ECG recording/monitoring should be recorded by the site and noted in the electronic case report form (REDCap, Vanderbilt University, TN, United States).

For peer review only

The trial will not collect any data from the standard recordings apart from its duration, and containment of AF. ECG recordings of newly diagnosed AF in the standard care arm will be collected and stored in the trial.

Outcomes

Primary endpoint

Difference between the two arms in a combined endpoint of recurrent ischemic stroke, intracerebral bleeding, and all-cause mortality during at least 36 months of follow-up.

Secondary endpoints

- Individual components of the primary endpoint
- Major bleeding including Intracranial bleeding
- Myocardial infarction
- Pacemaker implantation

Participant timeline intervention arm

Participants will be screened for consent during admission for ischemic stroke or TIA, within 14 days from admission. ECG investigation of 0-48 hours prior to randomisation is allowed. Extended ECG recording is typically initiated during admission for ischemic stroke/TIA and the participant will return the device via mail using prepaid envelope. The second extended ECG recording should be initiated at follow-up visit or by mailing the device to the

For peer review only - http://bmjopen.bmj.com/site/about/guidelines.xhtml

participants for self-application. Following completion of second 14-day ECG recording, no further personal visits are made in the trial.

Participant timeline standard arm

Starting or completing the ECG recording in the standard arm following discharge is allowed. Following completion of the ECG investigation initiated in the standard arm, no further personal visits are made in the trial.

Sample size

The sample size was calculated based on the primary outcome and performed by Karolinska Institutet Biostatistics core facility.

The event rate of recurrent ischemic stroke, intracerebral bleeding and all-cause mortality was estimated in patients admitted for ischemic stroke or TIA. Event rate was estimated in patients with AF with and without OAC treatment and in patients without AF. A complete list of studies used for estimations in the sample size calculation is reported in the appendix. Most studies used in this estimation reported data from patient cohorts with a lower age than in the AF SPICE trial. This could lead to an underestimation of event rates. Recent publications on AF status and outcomes in stroke patients in the projected age group is not available at the time of trial start. For instance, the MonDAFIS study[13] reported a mean (±SD) age of 66 (12.5) years overall, i.e., four years lower than the lowest age allowed in AF SPICE.

For the estimations of AF detection proportions, available studies on short-term (48 ±24 hours) ECG recording following stroke and corresponding studies on long-term (14 days)

For peer review only - http://bmjopen.bmj.com/site/about/guidelines.xhtml

recordings were used. A list of the AF detection studies available for this estimation is reported in the appendix.

The assumptions for the sample size calculation are reported in table 3. The complete sample size calculation is provided in the appendix. For the yearly risk of the composite endpoint, we estimate 17,7% in patients with AF and no OAC treatment, 7,3% in patients with AF and OAC treatment and 5% risk in patients without AF. We further estimate an AF detection proportion of 6% in the control group and 19% after two ECG recordings in the intervention group, and 13% with untreated AF in the control group, resulting in a sample size of 2718 for three years of fixed follow-up time after inclusion. Adding 20% for participant dropouts and treatment discontinuation, a total sample size of 3262 is needed, corresponding to 1631 in each arm based on three years follow-up, 80% power and double-sided significance level of 5%. With current sample size calculation, the study is powered to detect a 9% reduction of the composite endpoint in the group with extended ECG screening.

Recruitment

Patients will be screened and included during ischemic stroke or TIA admissions; most patients will be included in stroke units. Participating centres report at least 200 stroke/TIA admissions yearly. With an anticipated participation of 30 sites, they need to randomise 0.6 patients weekly during three years of inclusion to fulfil the recruitment target of 3262 participants.

For peer review only

Allocation

Participants will be randomly assigned to either control or intervention group with a 1:1 allocation as per a computer-generated randomisation schedule stratified by site in the online REDCap database. Randomisation will be performed immediately following the collection of necessary baseline data according to table 5.

Blinding

Due to the nature of the intervention, neither participants nor staff will be blinded to allocation.

Data collection methods primary Outcome (Table 4a)

Primary endpoint variables are collected from Swedish health care registers as specified in Table 4a.

Data collection methods Secondary and exploratory outcomes (Table 4b)

Variables collected for secondary and exploratory outcomes are reported in table 4b.

Baseline data entered at inclusion all participants (Table 5)

Data entered at baseline in all participants are listed in table 5. To lowering the threshold for inclusion, the remaining baseline variables will be collected from the Riksstroke register[5].

Baseline data entered in control and intervention arms (Appendix)

Data entered at baseline in standard and interventions arms are listed in the Appendix.

For peer review only

For peer review only - http://bmjopen.bmj.com/site/about/guidelines.xhtml

Baseline register data from Riksstroke register (Appendix)

Baseline data from Riksstroke are listed in the appendix.

ECG data (Appendix)

There are different ECG variables for the control and intervention arms.

1. In the control arm, the local investigator or delegate will report if ECG was recorded using ECG telemetry and/or Holter recording, the duration of each recording, and whether AF was diagnosed. ECG variables for the standard arm are listed in the appendix.

2. In the intervention arm, ECG data from patch recordings will be stored in the patch hardware and returned by the participant using a pre-paid envelope. Data from ECG patch hardware will be uploaded by the patch provider into the Cardiologs database. Using the Cardiologs system, ECG patch data will be scrutinized by the core reading team which also enter ECG data manually into the REDCap database. In the case of insufficient amount of analysable ECG signal time (<7 days), additional ECG patches will be offered to the participant and the reason entered into the REDCap database.

For the second ECG patch recording initiated 2-4 months following randomisation, data will be collected on whether the patch is applied within health care or by the participant. Reasons for not performing the second ECG patch recording will also collected.

ECG variables collected in the intervention group are listed in the appendix.

For peer review only

For peer review only - http://bmjopen.bmj.com/site/about/guidelines.xhtml

Retention

At the majority of including sites, the second visit for the intervention group coincides with an outpatient follow-up visit after stroke, which will give the participant motivation for attending the visit. Patients receive no reimbursement for participation.

Statistical Methods

The main analysis of outcome will be based on time to event (endpoint) and performed according to the intention to treat principle. The cumulative incidence of the combined endpoint and its individual components will be presented for each treatment group (standard and extended ECG).

For the primary analysis, of the combined endpoint, the Log-rank test will be used to test for treatment group differences. Cox proportional hazard regression will be used to estimate the hazard ratios with 95% confidence intervals, and to analyse associations with atrial fibrillation incidence and/or effect modification by age, comorbidity, biometrics, and supraventricular activity.

The secondary analysis, of each individual components of the primary endpoint, will account for competing risks. We will use cause-specific models (where the rate of each individual endpoint is based on patients who have not yet experienced any of the endpoints) and subdistributional hazard models (where the rate is based on patients who have not yet

For peer review only

For peer review only - http://bmjopen.bmj.com/site/about/guidelines.xhtml

experience the endpoint under study). Other analysis, including baseline characteristics, will be presented by proportions (binary variables) and means (continuous variables) with 95% confidence intervals in each ECG group. Corresponding group comparisons will be carried out by χ^2-tests and t-tests. An outcome with a p-value < 0.05 will be considered statistically significant.

Data Monitoring Committee

A Data Safety and Monitoring Board (DSMB) will be appointed at the start of the main trial, i.e., after the pilot phase. The Board will have a multidisciplinary composition and will be independent from the trial organisers. The DSMB will have a consulting role to the steering committee. The DSMB will monitor:

1. Cumulative inclusion rate, balance in baseline characteristics, duration and yield of ECG recordings during the inclusion phase and provide statements with conclusions to the steering committee annually.

2. Event rate in the standard and intervention group annually and provide statements with conclusion to the steering committee.

Harms

All diagnostic tools and treatments used in the trial are approved by regulatory bodies. Any adverse events noted in connection to investigations or treatment should be reported to authorities according to practice.

For peer review only

Auditing

A monitoring plan for the trial has been established, with the following areas:

1. Investigator and delegates qualifications, delegation log
2. Written consent forms, accuracy, and completeness.
3. Subject eligibility
4. Source data entered into CRF at inclusion, accuracy and completeness
5. CRF entries in REDCap, unverified and incomplete entries by site
6. Independent reader will scrutinize 5-10% of ECG reports in intervention arm.

ETHICS AND DISSEMINATION

Independent ethics committee

Ethical approval has been provided by the Swedish Ethical Review Authority (Reference 2021-02770). Before entering the trial, verbal and written information is given to the patient and informed consent form is signed. The trial will be conducted according to the ethical principles of the declaration of Helsinki[23] and national regulatory standards.

Ethical considerations

The optimal duration of the ECG investigation following ischemic stroke/TIA is not known, since no study so far has been powered to detect differences in hard endpoints such as

For peer review only

recurrent stroke or mortality between different ECG recording modalities, and this knowledge gap is acknowledged by the 2020 ESC AF guidelines[18].

The ethical issues in this trial are partly connected to the knowledge gap of ECG monitoring duration and the clinical practice that has evolved. The minimum recommendation for ECG monitoring duration is 24-48 hours of continuous ECG recording. Several stroke units are applying ECG monitoring protocols with longer duration. Randomising patients to a monitoring protocol with shorter ECG screening duration than applied in clinical practice could be regarded as lowering investigation ambitions. Since the long-term efficacy of different ECG monitoring strategies is unknown, the randomisation to one of the arms in this trial is considered ethically balanced against the option of continuing using monitoring strategies with unknown efficacy and unknown cost effectiveness.

Protocol amendments

Important modifications to the protocol will be summarized consecutively in the protocol and disseminated by mail and email to investigators. Major protocol changes, i.e. changes in eligibility criteria or patient information texts are subjected to ethical review.

Consent

Trained research nurses or investigators will introduce the trial orally. Patients will receive written information about the trial. Research nurses or investigators will discuss the trial in light of the written information given, and patients will have an opportunity to discuss the information and ask questions about participation.

For peer review only - http://bmjopen.bmj.com/site/about/guidelines.xhtml

In patients with aphasia or inability to write following stroke, it seems reasonable to collect informed consent from next of kin, i.e. a spouse or other close relative if some other mode of communication is established between the patient and the next of kin.

Confidentiality

At the trial sites, all log lists containing personal identification data will be stored securely. All trial data will be stored in a REDCap database, accessible only by a password and SUNET eduID access. All actions in the REDCap database are logged. Data in the ECG reading system Cardiologs are pseudonymized and accessible with password.

For peer review only

For peer review only - http://bmjopen.bmj.com/site/about/guidelines.xhtml

AUTHORS´ CONTRIBUTIONS

JE conceived of the trial and drafted the protocol. PW, KS, SÅ, ME and EI made major contributions to trial design. ER, ES, BN, MvE, KH, WG, JBS and SS made contributions to trial design. Power calculation was performed by Karolinska Institutet Biostatics Core Facility. AF SPICE is conducted in collaboration with the Swedish Stroke Register, Riksstroke.

FUNDING STATEMENT

AF SPICE is funded by grants from the Swedish Research Council, the Swedish Heart & Lung Foundation, the Swedish Stroke Foundation, and the Stockholm Region (ALF). The funding sources had no role in the design of this trial and will not have any role during its execution, analyses, interpretation of the data, or decision to submit results.

ACKNOWLEDGEMENT

We are grateful to Ida Hed Myrberg and Letizia Orsini at Karolinska Institutet Biostatistics Core Facility for assistance with the power calculation.

COMPETEING INTEREST STATEMENT

JE has received consultant or lecture fees from Roche Diagnostics, Pfizer, Bristol Myers Squibb, Boehringer Ingelheim, Piotrode and Philips. Research grants from the Swedish Research Council, The Swedish Heart&Lung Foundation, The Swedish Innovation Agency and

For peer review only

The Stockholm Region. KS, EI report no disclosures. ES reports consultant or lecture fees from Bayer, Bristol-Myers Squibb, Pfizer, Boehringer-Ingelheim, Johnson & Johnson, Merck Sharp & Dome. Research grants from the Swedish Research Council, The Swedish Heart&Lung Foundation, Åke Wibergs Foundation and CIMED. BN reports Data Safety Monitoring Board fees from Astra Zeneca. MvE is the chairman of Riksstroke, the Swedish Stroke Register. JOS reports no disclosures. SÅ reports lecture fees paid to the institution from Bristol-Myers Squibb, Pfizer, Boehringer-Ingelheim, and is a member of the steering committee of Riksstroke, the Swedish Stroke Register. PW reports consulting fees, fee as a Clinical Events Committee member and unrestricted grants from Abbott. PW is a member of the steering committee of Riksstroke, the Swedish Stroke Register.

Access to data

The investigators will have access to the final dataset without any restricions.

For peer review only

REFERENCES

1. Global, regional, and national burden of stroke and its risk factors, 1990-2019: a systematic analysis for the Global Burden of Disease Study 2019. *Lancet Neurol*. 2021;20:795-820.

2. Hankey GJ. Stroke. *Lancet*. 2017;389:641-654.

3. O'Donnell MJ, Chin SL, Rangarajan S, Xavier D, Liu L, Zhang H, Rao-Melacini P, Zhang X, Pais P, Agapay S, Lopez-Jaramillo P, Damasceno A, Langhorne P, McQueen MJ, Rosengren A, Dehghan M, Hankey GJ, Dans AL, Elsayed A, Avezum A, Mondo C, Diener HC, Ryglewicz D, Czlonkowska A, Pogosova N, Weimar C, Iqbal R, Diaz R, Yusoff K, Yusufali A, Oguz A, Wang X, Penaherrera E, Lanas F, Ogah OS, Ogunniyi A, Iversen HK, Malaga G, Rumboldt Z, Oveisgharan S, Al Hussain F, Magazi D, Nilanont Y, Ferguson J, Pare G and Yusuf S. Global and regional effects of potentially modifiable risk factors associated with acute stroke in 32 countries (INTERSTROKE): a case-control study. *Lancet*. 2016;388:761-75.

4. Eriksson M, Åsberg S, Sunnerhagen KS and von Euler M. Sex Differences in Stroke Care and Outcome 2005-2018: Observations From the Swedish Stroke Register. *Stroke*. 2021:Strokeaha120033893.

5. Riksstroke english summary 2021. https://www.riksstroke.org/annual-reports-and-graphs/. Accessed date 2023-01-15.

For peer review only - http://bmjopen.bmj.com/site/about/guidelines.xhtml

6. Mukundan G and Seidenwurm DJ. Economic and Societal Aspects of Stroke Management. *Neuroimaging Clin N Am*. 2018;28:683-689.

7. Henninger N, Goddeau RP, Jr., Karmarkar A, Helenius J and McManus DD. Atrial Fibrillation Is Associated With a Worse 90-Day Outcome Than Other Cardioembolic Stroke Subtypes. *Stroke*. 2016;47:1486-92.

8. Bjorck S, Palaszewski B, Friberg L and Bergfeldt L. Atrial fibrillation, stroke risk, and warfarin therapy revisited: a population-based study. *Stroke*. 2013;44:3103-8.

9. Hart RG, Pearce LA and Aguilar MI. Meta-analysis: antithrombotic therapy to prevent stroke in patients who have nonvalvular atrial fibrillation. *Ann Intern Med*. 2007;146:857-67.

10. Gladstone DJ, Spring M, Dorian P, Panzov V, Thorpe KE, Hall J, Vaid H, O'Donnell M, Laupacis A, Cote R, Sharma M, Blakely JA, Shuaib A, Hachinski V, Coutts SB, Sahlas DJ, Teal P, Yip S, Spence JD, Buck B, Verreault S, Casaubon LK, Penn A, Selchen D, Jin A, Howse D, Mehdiratta M, Boyle K, Aviv R, Kapral MK and Mamdani M. Atrial fibrillation in patients with cryptogenic stroke. *N Engl J Med*. 2014;370:2467-77.

11. Grond M, Jauss M, Hamann G, Stark E, Veltkamp R, Nabavi D, Horn M, Weimar C, Kohrmann M, Wachter R, Rosin L and Kirchhof P. Improved Detection of Silent Atrial

For peer review only

For peer review only - http://bmjopen.bmj.com/site/about/guidelines.xhtml

Fibrillation Using 72-Hour Holter ECG in Patients With Ischemic Stroke: A Prospective Multicenter Cohort Study. *Stroke*. 2013;44:3357-64.

12. Sanna T, Diener HC, Passman RS, Di Lazzaro V, Bernstein RA, Morillo CA, Rymer MM, Thijs V, Rogers T, Beckers F, Lindborg K and Brachmann J. Cryptogenic stroke and underlying atrial fibrillation. *N Engl J Med*. 2014;370:2478-86.

13. Wachter R, Groschel K, Gelbrich G, Hamann GF, Kermer P, Liman J, Seegers J, Wasser K, Schulte A, Jurries F, Messerschmid A, Behnke N, Groschel S, Uphaus T, Grings A, Ibis T, Klimpe S, Wagner-Heck M, Arnold M, Protsenko E, Heuschmann PU, Conen D, Weber-Kruger M, Find AFI and Coordinators. Holter-electrocardiogram-monitoring in patients with acute ischaemic stroke (Find-AFRANDOMISED): an open-label randomised controlled trial. *Lancet Neurol*. 2017;16:282-290.

14. Haeusler KG, Kirchhof P, Kunze C, Tütüncü S, Fiessler C, Malsch C, Olma MC, Jawad-Ul-Qamar M, Krämer M, Wachter R, Michalski D, Kraft A, Rizos T, Gröschel K, Thomalla G, Nabavi DG, Röther J, Laufs U, Veltkamp R, Heuschmann PU and Endres M. Systematic monitoring for detection of atrial fibrillation in patients with acute ischaemic stroke (MonDAFIS): a randomised, open-label, multicentre study. *Lancet Neurol*. 2021;20:426-436.

15. NICE AF post stroke Guidelines. https://www.nice.org.uk/guidance/dg41/. Accessed date 2023-01-15.

For peer review only

For peer review only - http://bmjopen.bmj.com/site/about/guidelines.xhtml

16. Andrade JG, Aguilar M, Atzema C, Bell A, Cairns JA, Cheung CC, Cox JL, Dorian P, Gladstone DJ, Healey JS, Khairy P, Leblanc K, McMurtry MS, Mitchell LB, Nair GM, Nattel S, Parkash R, Pilote L, Sandhu RK, Sarrazin JF, Sharma M, Skanes AC, Talajic M, Tsang TSM, Verma A, Verma S, Whitlock R, Wyse DG and Macle L. The 2020 Canadian Cardiovascular Society/Canadian Heart Rhythm Society Comprehensive Guidelines for the Management of Atrial Fibrillation. *Can J Cardiol*. 2020;36:1847-1948.

17. Brieger D, Amerena J, Attia J, Bajorek B, Chan KH, Connell C, Freedman B, Ferguson C, Hall T, Haqqani H, Hendriks J, Hespe C, Hung J, Kalman JM, Sanders P, Worthington J, Yan TD and Zwar N. National Heart Foundation of Australia and the Cardiac Society of Australia and New Zealand: Australian Clinical Guidelines for the Diagnosis and Management of Atrial Fibrillation 2018. *Heart, lung & circulation*. 2018;27:1209-1266.

18. Hindricks G, Potpara T, Dagres N, Arbelo E, Bax JJ, Blomström-Lundqvist C, Boriani G, Castella M, Dan GA, Dilaveris PE, Fauchier L, Filippatos G, Kalman JM, La Meir M, Lane DA, Lebeau JP, Lettino M, Lip GYH, Pinto FJ, Thomas GN, Valgimigli M, Van Gelder IC, Van Putte BP and Watkins CL. 2020 ESC Guidelines for the diagnosis and management of atrial fibrillation developed in collaboration with the European Association for Cardio-Thoracic Surgery (EACTS): The Task Force for the diagnosis and management of atrial fibrillation of the European Society of Cardiology (ESC) Developed with the special contribution of the European Heart Rhythm Association (EHRA) of the ESC. *Eur Heart J*. 2021;42:373-498.

For peer review only

19. Kleindorfer DO, Towfighi A, Chaturvedi S, Cockroft KM, Gutierrez J, Lombardi-Hill D, Kamel H, Kernan WN, Kittner SJ, Leira EC, Lennon O, Meschia JF, Nguyen TN, Pollak PM, Santangeli P, Sharrief AZ, Smith SC, Jr., Turan TN and Williams LS. 2021 Guideline for the Prevention of Stroke in Patients With Stroke and Transient Ischemic Attack: A Guideline From the American Heart Association/American Stroke Association. *Stroke*. 2021;52:e364-e467.

20. National guideline for stroke. https://www.strokeaudit.org/Guideline/Full-Guideline.aspx. Accessed date 2023-01-15

21. Rubiera M, Aires A, Antonenko K, Lémeret S, Nolte CH, Putaala J, Schnabel RB, Tuladhar AM, Werring DJ, Zeraatkar D and Paciaroni M. European Stroke Organisation (ESO) guideline on screening for subclinical atrial fibrillation after stroke or transient ischaemic attack of undetermined origin. *Eur Stroke J*. 2022;7:Vi.

22. Schnabel RB, Haeusler KG, Healey JS, Freedman B, Boriani G, Brachmann J, Brandes A, Bustamante A, Casadei B, Crijns H, Doehner W, Engstrom G, Fauchier L, Friberg L, Gladstone DJ, Glotzer TV, Goto S, Hankey GJ, Harbison JA, Hobbs FDR, Johnson LSB, Kamel H, Kirchhof P, Korompoki E, Krieger DW, Lip GYH, Lochen ML, Mairesse GH, Montaner J, Neubeck L, Ntaios G, Piccini JP, Potpara TS, Quinn TJ, Reiffel JA, Ribeiro ALP, Rienstra M, Rosenqvist M, Sakis T, Sinner MF, Svendsen JH, Van Gelder IC, Wachter R, Wijeratne T and Yan B. Searching for Atrial Fibrillation Poststroke: A White Paper of the AF-SCREEN International Collaboration. *Circulation*. 2019;140:1834-1850.

For peer review only

For peer review only - http://bmjopen.bmj.com/site/about/guidelines.xhtml

23. World Medical Association Declaration of Helsinki: ethical principles for medical research involving human subjects. *Jama*. 2013;310:2191-4.

For peer review only

For peer review only - http://bmjopen.bmj.com/site/about/guidelines.xhtml

Table 1 ECG screening post stroke society recommendations

Society	Recommendation Summary	Year	Ref
European Stroke Organisation	In adult patients with ischaemic stroke or TIA of undetermined origin, we recommend longer duration of cardiac rhythm monitoring of more than 48 h and if feasible with ILR to increase the detection of subclinical AF.	2022	21
American Heart Association/American Stroke Association	Long-term rhythm monitoring with mobile cardiac outpatient telemetry, implantable loop recorder, or other approach is reasonable to detect intermittent AF	2021	19
European Society of Cardiology	Short-term ECG recording for at least the first 24 h, followed by continuous ECG monitoring for at least 72 h whenever possible.<br><br>Long-term non-invasive ECG monitors or insertable cardiac monitors should be considered, to detect AF.	2020	18
Swedish board of health and welfare	Health care should provide long-term ECG for 24-48 h.<br><br>long-term ECG for more than 48 h duration using Holter recording or inpatient telemetry ECG in selected cases.	2020	
NICE	Reveal LINQ is recommended as an option to help to detect atrial fibrillation after cryptogenic stroke only if:<br><br>non-invasive electrocardiogram (ECG) monitoring has been done and a cardiac arrhythmic cause of stroke is still suspected.	2020	15
Canadian Cardiovascular Society/Canadian Heart Rhythm Society	At least 24 hours of ambulatory ECG monitoring to identify AF in patients with nonlacunar ESUS.<br>Additional monitoring for AF detection (eg, prolonged external loop recorder or implantable cardiac monitoring, where available) be performed for<br>selected older patients with nonlacunar ESUS in whom AF is suspected but unproven	2020	16
National Heart Foundation of Australia and the Cardiac Society of Australia and New Zealand	For patients with Embolic Stroke of Uncertain Source, longer term ECG monitoring (external or implantable) should be used.	2018	17
Royal College of Physicians	A. People with ischaemic stroke or TIA should undergo a period of prolonged (at least 12 hours) cardiac monitoring.<br>B. Prolonged ECG monitoring (24 hours or longer), particularly if they have a pattern of cerebral ischaemia on brain imaging suggestive of cardioembolism.	2016	20

For peer review only

For peer review only - http://bmjopen.bmj.com/site/about/guidelines.xhtml

Table 2. Prognostic ECG findings on extended ECG recording which should be communicated to the local investigator.

Ventricular tachycardia
Excessive ventricular extrasystoles (> 5%)
Pause > 3 seconds
AV-block type II and III
Bradycardia < 40/minute

For peer review only

For peer review only - http://bmjopen.bmj.com/site/about/guidelines.xhtml

**Endpoint* risk in patients with AF and OAC treatment**	**7.3%**
**Endpoint risk in patients with AF and no OAC treatment**	**17.7%**
**Endpoint risk in patients without AF**	**5%**
**AF detection proportion in standard arm****	**6%**
**AF detection proportion in intervention arm****	**19%**

*Endpoint including recurrent stroke, all-cause mortality, and intracerebral bleeding.**Including AF detection during follow-up.

Table 3. Event rates and atrial fibrillation detection rates used in sample size calculation.

For peer review only

For peer review only - http://bmjopen.bmj.com/site/about/guidelines.xhtml

Table 4a Primary outcome

Variable	Coding ICD-10	Source	Validity
Ischemic stroke	I63	Riksstroke	High
Stroke not specified	I64	Riksstroke	High
All-cause mortality	N/A	National register of death	Very high
Intracerebral bleeding (spontaneous)	I61	Riksstroke	High

Table 4b Secondary outcome

<table>
<tr><th>Variable</th><th colspan="2">Coding ICD-10</th><th>Source</th></tr>
<tr><td>Myocardial infarction</td><td colspan="2">I21, I22, I25.2</td><td rowspan="9">National inpatient register, Riksstroke</td></tr>
<tr><td rowspan="5">Major bleeding</td><td>Gastrointestinal bleeding</td><td>K226, K250, K252, K254, K256, K260, K262, K264, K266, K270, K272, K274, K276, K280, K282, K284, K286, K290, K625, K661, K920, K921, K922, I850, I983</td></tr>
<tr><td>Urogenital bleeding</td><td>N02, R319, N939, N950, N501A</td></tr>
<tr><td>Other bleeding</td><td>H113, H313, H356, H431, H450, H922, I312, J942, M250, R04, R58, T810, D500, D629, T792</td></tr>
<tr><td>Sub-, epidural and subarachnoid haemorrhages (incl traumatic)</td><td>I60, I61, I62, S064, S065, S066</td></tr>
<tr><td>Procedure codes for transfusion</td><td>DR029, DR033, Z513</td></tr>
<tr><td>Atrial fibrillation/flutter</td><td colspan="2">I48</td></tr>
<tr><td>Pacemaker implantation</td><td colspan="2">FPE00, FPE10, FPE20, FPE26</td></tr>
<tr><td>OAC and antiplatelet treatment, NSAID</td><td colspan="2">B01A, N02BA, M01A</td><td>National drug dispense register</td></tr>
</table>

For peer review only

Table 5 Variables entered at incluision

Variable	Unit
Record ID (automatically generated)	
Sex	Male/Female
Date of birth	Date
Social Security Number	Special format
Date of Stroke/TIA	Date
Age	Years
Height	cm
Weight	kg
Hemoglobin	g/l
Estimated GFR	ml/min
Site identification	Choice from list
Consent Date	Date
Randomization date	Date

For peer review only

Figure 1.

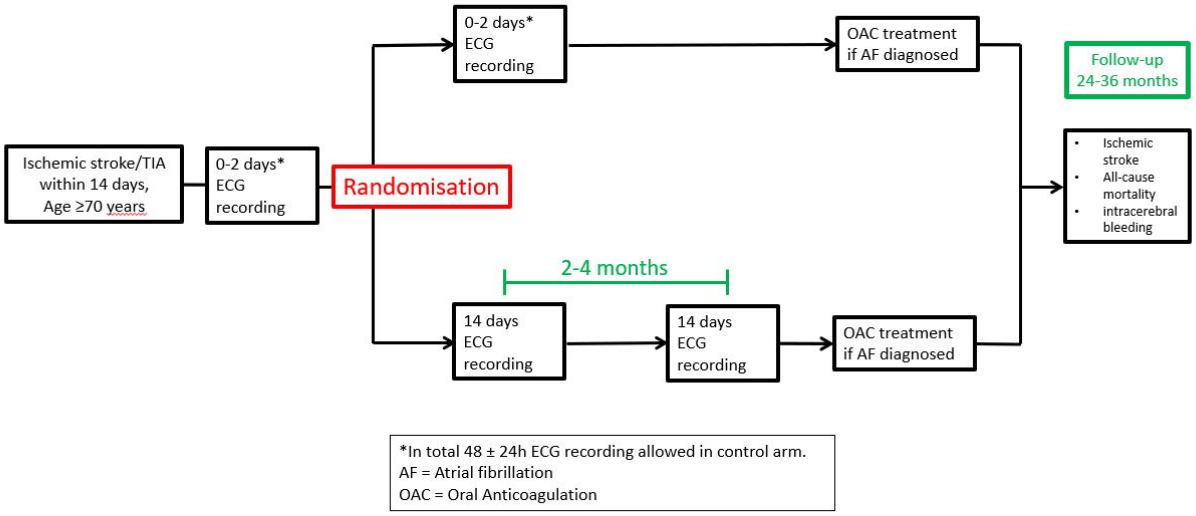

For peer review only

Supplementary Appendix

Table of contents

Trials in eventrates power calculation 2
Appendix table Trials in ECG detection rate power calculation..3
Baseline data collected from Riksstroke register 4
ECG Data collected by local investigator in standard arm 5
Data collected by local investigator in intervention arm 5
Sample size calculation 12

For peer review only

For peer review only - http://bmjopen.bmj.com/site/about/guidelines.xhtml

Trials in eventrates power calculation

	AF+/OAC+			AF+/OAC-			AF-/OAC-		
	**Rate**	**Mean Age**	**Reference**	**Rate**	**Mean Age**	**Reference**	**Rate**	**Mean Age**	**Reference**
**Stroke**	2,23%	70,2	Diener[1] 2010 D110	5,30%	71,7	Diener[2] 2012	5,40%	73	Netland el al 2019
	1,91%	70,8	Diener[1] 2010 D150				5,10%	66	Amarenco[3] 2016
	2,53%	70,4	Diener[1] 2010 W				3,60%	70,1	Andersen[4] 2015
	2,39%	71,7	Diener[2] 2012 A				5,40%		Wachter[5] 2017
	2,79%	71	Hankey[6] 2012 R						
	2,96%	71	Hankey[6] 2012 W						
	2,26%	70,1	Easton[7] 2012 A						
	3,17%	70,1	Easton[7] 2012 W						
	2,44%		Rost[8] 2016 E						
**All-cause mortality**	3,24%	70,2	Diener[1] 2010 D110	6,86%	71,7	Diener[2] 2012	10,70%	73	Netland el al 2019
	4,39%	70,8	Diener[1] 2010 D150				1,80%	66	Amarenco[3] 2016
	4,58%	70,4	Diener[1] 2010 W				10,50%	70,1	Andersen[4] 2015
	5,78%	71,7	Diener[2] 2012 A				4,80%		Wachter[5] 2017
	4,40%	71	Hankey[6] 2012 R				10,60%		Lip[9] 2017
	4,54%	71	Hankey[6] 2012 W						
	4,22%	70,1	Easton[7] 2012 A						
	4,77%	70,1	Easton[7] 2012 W						
	4,35%		Rost[8] 2016 E						
**Intracerebral bleeding**	0,25%	70,2	Diener[1] 2010 D110	1,56%	71,7	Diener[2] 2012	0,40%	66	Amarenco[3] 2016
	0,53%	70,8	Diener[1] 2010 D150						
	1,28%	70,4	Diener[1] 2010 W						
	1,17%	71,7	Diener[2] 2012 A						
	0,59%	71	Hankey[6] 2012 R						
	0,80%	71	Hankey[6] 2012 W						
	0,55%	70,1	Easton[7] 2012 A						
	1,49%	70,1	Easton[7] 2012 W						
	0,62%		Rost[8] 2016 E						
**Mortal bleeding**	0,26%	71	Hankey[6] 2012 R						
	0,49%	71	Hankey[6] 2012 W						

AF = Atrial Fibrillation OAC = Oral Anticoagulant

For peer review only - http://bmjopen.bmj.com/site/about/guidelines.xhtml

Appendix table Trials in ECG detection rate power calculation

Publication Short-term external ECG after stroke/TIA	Detection rate (%)
Jabaudon[10] 2004	5.0
Shafqat[11] 2004	2.4
Alhadramy[12] 2010	2.5
Rizos[13] 2010	2.5
Doliwa-Sobocinski[14] 2012	2.0
Lazarro[15] 2012	6.0
Gumbinger[16] 2012	1.0
Suissa[17] 2012	2.3
Thakkar[18] 2014	5.8
Bansil[19] 2004	4.7

Publication Long-term external ECG after stroke/TIA	Detection rate (%)
Barthelemy[20] 2003	14.3
Jabadoun[10] 2004	5.7
Wallman[21] 2007	14.2
Tayal[22] 2008	5.0
Elijiovich[23] 2009	20.0
Bhatt[24] 2011	24.2
Flint[25] 2012	11.0
Higgins[26] 2013	8.0
Miller[27] 2013	17.3
Gladstone[28] 2014	16.1
Sebasigari[29] 2017	11.7

For peer review only - http://bmjopen.bmj.com/site/about/guidelines.xhtml

Baseline data collected from Riksstroke register

Reporting Hospital	Statin treatment on admission
Date of symptom onset	Aspirin treatment on admission
Time of symptom onset	Clopidogrel treatment on admission
Level of consciousness at admission	
NIHSS at admission	Dipyridamole treatment on admission
Number of days admitted	Other antiplatelet treatment on admission
Number of days in stroke unit	Hypertension treatment on admission
	Dipyridamole treatment on admission
Diagnosis	Warfarin treatment on admission
Age at admission	Apixaban treatment on admission
Sex	Dabigatran treatment on admission
Level of assistance	Rivaroxaban treatment on admission
Living alone	Edoxaban treatment on admission
Level of dependence	
Assisted dressing	Statin treatment at discharge
Assisted personal hygiene	Aspirin treatment at discharge
Independence in locomotion	Clopidogrel treatment at discharge
	Dipyridamole treatment at discharge
Previously diagnosed AF	Warfarin treatment at discharge
AF diagnosed on arrival	Hypertension treatment at discharge
Previously or newly diagnosed diabetes	Apixaban treatment at discharge
Hypertension (treated) on admission	Dabigatran treatment at discharge
Smoking status	Rivaroxaban treatment at discharge
CT brain scan performed during admission	Reason for withholding OAC treatment
MRI brain scan performed during admission	
Carotide artery ultrasound brain scan performed during admission	Edoxaban treatment at discharge
CT angiography performed during admission	
Longterm ECG (at least 24 h) performed during admission	
Thrombolytic treatment administered	
Type of ward at admission	

For peer review only - http://bmjopen.bmj.com/site/about/guidelines.xhtml

ECG Data collected by local investigator in standard arm

Variable	Unit
Inpatient ECG Telemetry used	Yes/No
Duration of ECG Telemetry	Hours
AF diagnosed during ECG Telemetry	Yes/No
Holter Recording used	Yes/No
Duration of Holter Recording	Hours
AF diagnosed during Holter	Yes/No

For peer review only

Data collected by local investigator in intervention arm

Variable	Unit
ePatch number 1 serial number	Integer
Date epatch number 1 recording start	Date
Additional epatch number 1 serial number	Integer
Site of application additional epatch number 1	Health care/self-application
Date for mailing out additional epatch number 1	Date
Date additional epatch number 1 recording start	Date
Reason for additional epatch	1. epatch detachment<br>2. Red light indicated

For peer review only - http://bmjopen.bmj.com/site/about/guidelines.xhtml

	3. Poor signal quality<br>4. MRI examination<br>5. Other radiology<br>6. Thoracic/neck surgery<br>7. Other reason
Additional epatch number 1 serial number	Integer
Site of application additional epatch number 1	Health care/self-application
Date for mailing out additional epatch number 1	Date
Date additional epatch number 1 recording start	Date
Reason for additional epatch number 1	1. epatch detachment<br>2. Red light indicated<br>3. Poor signal quality<br>4. MRI examination<br>5. Other radiology<br>6. Thoracic/neck surgery<br>7. Other reason
Mode of application epatch number 2	Health care/self-application
Date for mailing of epatch number 2 to participant	Date
Date of application epatch number 2	Date
Epatch number 2 serial number	Integer
First additional epatch number 2 serial number	Integer
Mode of application first additional epatch number 2	Health care/self-application
Date for mailing of first additional epatch number 2 to participant	Date
Date of application of first additional epatch number 2	
Reason for first additional epatch number 2	1. epatch detachment<br>2. Red light indicated<br>3. Poor signal quality<br>4. MRI examination<br>5. Other radiology

For peer review only - http://bmjopen.bmj.com/site/about/guidelines.xhtml

	6. Thoracic/neck surgery<br>7. Other reason
Second additional epatch number 2 serial number	Integer
Mode of application second additional epatch number 2	Health care/self-application
Date for mailing of second additional epatch number 2 to participant	Date
Date of application of second additional epatch number 2	
Reason for second additional epatch number 2	1. epatch detachment<br>2. Red light indicated<br>3. Poor signal quality<br>4. MRI examination<br>5. Other radiology<br>6. Thoracic/neck surgery<br>7. Other reason
Reason for not recording epatch number 2	1. Diagnosed with AF on epatch number 1<br>2. Participant declines recording<br>3. Participant decline further participation<br>4. Other reason

For peer review only - http://bmjopen.bmj.com/site/about/guidelines.xhtml

ECG Data collected in intervention group

Patch number 1	
**Variable**	**Unit**
Date for ECG data upload in Cardiologs	Date
Date for ECG reading	Date
Date for first additional ECG data upload in Cardiologs	Date
Date for first additional ECG reading	Date
Date for second additional ECG data upload in Cardiologs	Date
Date for second additional ECG reading	Date
Monitoring time	Hours
Analyzable time	Hours
Heart rate max	Bpm
Heart rate min	Bpm
Heart rate mean	Bpm
Proportion supraventricular ectopics	Percent
Proportion ventricular ectopics	Percent
Number of pauses > 2 seconds	Integer
Longest pause	Seconds, one decimal
AV-block II or III	Yes/No
Highest AV-block grade	1. AV-block II type 1<br>2. AV-block II type 2<br>3. AV-block III
Atrial fibrillation	Yes/No
Atrial flutter	Yes/No
Duration to first Atrial fibrillation/flutter	Hours
Atrial fibrillation/flutter subtype	1. Paroxysmal<br>2. Persistant

For peer review only - http://bmjopen.bmj.com/site/about/guidelines.xhtml

Atrial fibrillation/ flutter heart rate max	Bpm
Atrial fibrillation/ flutter heart rate mean	Bpm
Atrial fibrillation/ flutter burden	Percent
Atrial fibrillation/ flutter longest episode	Seconds
Number of Ventricular Tachycardia (VT)	n
Duration longest episode VT	Seconds
Number of Suptraventricular Tachycardia (SVT)	n
Duration longest episode SVT	Seconds
Proportion of noise	Percent
Action triggered by recording	1. Standard statement<br>2. Communication with local PI within 3 working days<br>3. Communication with local PI within one working day<br>4. Further assessment following patch number 2
**Patch number 2**	
**Variable**	**Unit**
Date for ECG data upload in Cardiologs	Date
Date for ECG reading	Date
Date for first additional ECG data upload in Cardiologs	Date
Date for first additional ECG reading	Date
Date for second additional ECG data upload in Cardiologs	Date
Date for second additional ECG reading	Date
Monitoring time	Hours
Analyzable time	Hours
Heart rate max	Bpm

For peer review only - http://bmjopen.bmj.com/site/about/guidelines.xhtml

Heart rate min	Bpm
Heart rate mean	Bpm
Proportion supraventricular ectopics	Percent
Proportion ventricular ectopics	Percent
Number of pauses > 2 seconds	Integer
Longest pause	Seconds, one decimal
AV-block II or III	Yes/No
Highest AV-block grade	1. AV-block II type 1<br>2. AV-block II type 2<br>3. AV-block III
Atrial fibrillation	Yes/No
Atrial flutter	Yes/No
Atrial fibrillation/flutter subtype	1. Paroxysmal<br>2. Persistant
Atrial fibrillation/ flutter heart rate max	Bpm
Atrial fibrillation/ flutter heart rate mean	Bpm
Atrial fibrillation/ flutter burden	Percent
Atrial fibrillation/ flutter longest episode	Seconds
Number of Ventricular Tachycardia (VT)	n
Duration longest episode VT	Seconds
Number of Suptraventricular Tachycardia (SVT)	n
Duration longest episode SVT	Seconds
Proportion of noise	Percent
Action triggered by recording	1. Standard statement<br>2. Communication with local PI within 3 working days<br>3. Communication with local PI within one working day

For peer review only - http://bmjopen.bmj.com/site/about/guidelines.xhtml

	4. Further assessment following patch number 2

For peer review only

Sample size calculation

Biostatistics
CORE FACILITY

Sample size calculation AF SPICE

Ida Hed Myrberg & Letizia Orsini

2021-10-08

Background

Sample size calculation for the AF SPICE study; a multicenter randomized register-based trail in Swedish stroke units.

Method

The function *cpower* in R package *Hmisc* is used. This is a function for calculating the power for a Cox Proportional Hazards model or log-rank test comparing two groups. The function assumes an underlying exponential distribution in both groups. To calculate the sample size needed to achieve 80% power, the function *uniroot* is used in combination with *cpower*.

How parameters are defined and calculated

In this section, it is shown how the yearly rate, and the 3-year event rate, of stroke/death/intracranial bleeding in the intervention and control group, respectively, are calculated based on the yearly rates in the three groups AF+/OAK+, AF+/OAK-, and AF-/OAK-, and on the proportions of these three groups in the intervention and control group. The proportion of AF+/OAK+, AF+/OAK-, and AF-/OAK- in the intervention and control group in Table 2 are examples, and in the next two sections they will be varied between 10-20% in the intervention group, and 3-12% in the control group. The yearly rate of stroke/death/intracranial bleeding for AF-/OAK- will be set to 5% and 8.4%, respectively, in two different scenarios.

Table 1 shows the assumed yearly rates of stroke/death/intracranial bleeding for the subgroups AF+/OAK+, AF+/OAK-, AF-/OAK-, while Table 2 shows the proportion of patients in each of the aforementioned subgroups, divided by interention and control.

Table 1: Yearly rate of stroke/death/intracranial bleeding depending on AF and treatment.

AF/treatment	Yearly rate of stroke/death/intracranial bleeding
AF+/OAK+	0.073
AF+/OAK-	0.177
AF-/OAK-	0.084

Table 2: Proportion of patients with AF and treatment in the intervention group and control group, respectively.

AF/treatment	Proportion in intervention group	Proportion in control group
AF+/OAK+	0.10	0.03
AF+/OAK-	0.00	0.07
AF-/OAK-	0.90	0.90

Biostatistics
CORE FACILITY

The yearly rate of stroke/death/intracranial in the intervention group is calculated as 0.073 * 0.1 + 0.177 * 0 + 0.084 * 0.9 = 0.0829. The yearly rate of stroke/death/intracranial in the control group is calculated as 0.073 * 0.03 + 0.177 * 0.07 + 0.084 * 0.9 = 0.09018.

Table 3: Yearly rate of stroke/death/intracranial bleeding in the intervention group and control group, respectively.

Group	Yearly rate of stroke/death/intracranial bleeding
Intervention group	0.0829
Control group	0.0902

The minimum follow-up time is assumed to be 3 years, and the recruitment time is set to 2 years.

The 3-year event rate in the control group, denoted *mc*, i.e. the proportion expected to have experienced the event by three years, is needed for the power calculation. The proportion not having experienced the event, i.e. 1-*mc*, is calculated using the Kaplan-Meier method, and the 3-year event rate is then calculated as 1-(1-*mc*)=*mc*. The 3-year event rate in the intervention group, *mi*, is calculated in a similar way. Both *mc* and *mi* are presented in Table 4.

Table 4: 3-year rate of stroke/death/intracranial bleeding in the intervention group and control group, respectively.

Group	3-year rate of stroke/death/intracranial bleeding
Intervention group	0.2287
Control group	0.2469

The percent reduction in the 3-year event rate for the intervention group compared to the control group is given by 1-22.87/24.69=7.38%.

Results: 5% yearly rate of stroke/death/intracranial bleeding for AF-/OAK-

In this section, a yearly event rate of 5% is assumed for the AF-/OAK- group.

The sample size needed is calculated for all combination of AF proportions, ranging from 10-20% in the intervention group, and 3-12% in the control group. Table 5 shows the percent reduction in the 3-year event rate for the intervention group compared to the control group, for different combinations of AF proportions.

Table 5: Percent reduction in the 3-year event rate for the intervention group compared to the control group. Columns: AF proportions in the control group. Rows: AF proportions in the intervention group.

	0.03	0.04	0.05	0.06	0.07	0.08	0.09	0.1	0.11	0.12
0.1	11.57	10.09	8.56	6.97	5.33	3.62	1.85	-	-	-
0.11	12.94	11.52	10.05	8.52	6.94	5.31	3.60	1.84	-	-

For peer review only - http://bmjopen.bmj.com/site/about/guidelines.xhtml

Biostatistics
CORE FACILITY

	0.03	0.04	0.05	0.06	0.07	0.08	0.09	0.1	0.11	0.12
0.12	14.25	12.89	11.47	10.01	8.49	6.92	5.28	3.59	1.83	-
0.13	15.52	14.20	12.83	11.42	9.96	8.45	6.89	5.26	3.57	1.82
0.14	16.73	15.46	14.14	12.78	11.38	9.92	8.42	6.86	5.24	3.56
0.15	17.89	16.66	15.40	14.09	12.73	11.33	9.88	8.38	6.83	5.21
0.16	19.01	17.82	16.60	15.34	14.03	12.68	11.29	9.84	8.35	6.80
0.17	20.09	18.94	17.76	16.54	15.28	13.98	12.63	11.24	9.80	8.31
0.18	21.12	20.01	18.87	17.69	16.48	15.22	13.92	12.58	11.20	9.76
0.19	22.12	21.05	19.94	18.80	17.63	16.41	15.16	13.87	12.53	11.15
0.2	23.08	22.04	20.97	19.87	18.73	17.56	16.35	15.10	13.82	12.48

Table 6 shows the total sample size needed to achieve 80% power to detect a statistically significant (with alpha=0.05) difference in the 3-year event rates presented in Table 5 between the intervention group and the control group, for different combinations of AF proportions.

Table 6: Total sample size needed for different combinations of AF proportions in the intervention and control group, respectively. Columns: AF proportions in the control group. Rows: AF proportions in the intervention group.

	0.03	0.04	0.05	0.06	0.07	0.08	0.09	0.1	0.11	0.12
0.1	8536	11504	16400	25370	44652	99460	393830	-	-	-
0.11	6628	8572	11552	16470	25480	44848	99900	395590	-	-
0.12	5310	6654	8608	11600	16542	25590	45044	100340	397348	-
0.13	4360	5332	6682	8644	11650	16612	25700	45240	100780	399108
0.14	3652	4378	5352	6710	8680	11698	16682	25810	45436	101220
0.15	3110	3668	4396	5374	6738	8716	11748	16752	25922	45630
0.16	2686	3124	3682	4412	5396	6764	8752	11796	16824	26032
0.17	2346	2696	3136	3696	4430	5418	6792	8788	11846	16894
0.18	2072	2356	2708	3148	3710	4448	5440	6820	8824	11894
0.19	1844	2080	2364	2718	3160	3726	4466	5462	6848	8860
0.2	1654	1850	2086	2374	2728	3172	3740	4484	5484	6876

Results: 8.4% yearly rate of stroke/death/intracranial bleeding for AF-/OAK-

In this section, a yearly event rate of 8.4% is assumed for the AF-/OAK- group.

The sample size needed is calculated for all combination of AF proportions, ranging from 10-20% in the intervention group, and 3-12% in the control group. Table 7 shows the percent reduction in the 3-year event rate for the intervention group compared to the control group, for different combinations of AF proportions.

Table 7: Percent reduction in the 3-year event rate for the intervention group compared to the control group. Columns: AF proportions in the control group. Rows: AF proportions in the intervention group.

	0.03	0.04	0.05	0.06	0.07	0.08	0.09	0.1	0.11	0.12
0.1	7.38	6.40	5.40	4.37	3.32	2.24	1.13	-	-	-
0.11	8.35	7.39	6.41	5.41	4.38	3.32	2.24	1.13	-	-

Biostatistics
CORE FACILITY

	0.03	0.04	0.05	0.06	0.07	0.08	0.09	0.1	0.11	0.12
0.12	9.30	8.36	7.40	6.42	5.41	4.38	3.33	2.25	1.14	-
0.13	10.23	9.31	8.37	7.41	6.43	5.42	4.39	3.33	2.25	1.14
0.14	11.14	10.24	9.32	8.38	7.42	6.44	5.43	4.39	3.34	2.25
0.15	12.04	11.16	10.26	9.34	8.40	7.43	6.45	5.44	4.40	3.34
0.16	12.91	12.05	11.17	10.27	9.35	8.41	7.44	6.45	5.44	4.41
0.17	13.78	12.93	12.07	11.19	10.28	9.36	8.42	7.45	6.46	5.45
0.18	14.62	13.79	12.95	12.08	11.20	10.30	9.37	8.43	7.46	6.47
0.19	15.45	14.64	13.81	12.96	12.10	11.22	10.31	9.39	8.44	7.47
0.2	16.26	15.47	14.66	13.83	12.98	12.12	11.23	10.33	9.40	8.45

Table 8 shows the total sample size needed to achieve 80% power to detect a statistically significant (with alpha=0.05) difference in the 3-year event rates presented in Table 5 between the intervention group and the control group, for different combinations of AF proportions.

Table 8: Total sample size needed for different combinations of AF proportions in the intervention and control group, respectively. Columns: AF proportions in the control group. Rows: AF proportions in the intervention group.

	0.03	0.04	0.05	0.06	0.07	0.08	0.09	0.1	0.11	0.12
0.1	13380	18096	25892	40198	71006	158734	630838	-	-	-
0.11	10296	13362	18072	25858	40144	70910	158518	629976	-	-
0.12	8176	10282	13346	18048	25824	40090	70814	158302	629114	-
0.13	6656	8166	10270	13328	18024	25790	40036	70718	158088	628250
0.14	5530	6648	8156	10256	13310	18000	25754	39982	70622	157872
0.15	4670	5522	6640	8144	10242	13292	17976	25720	39928	70526
0.16	3998	4664	5514	6630	8134	10228	13274	17952	25686	39874
0.17	3466	3994	4658	5508	6622	8124	10216	13258	17928	25652
0.18	3034	3460	3988	4652	5500	6614	8112	10202	13240	17904
0.19	2680	3030	3456	3984	4646	5494	6604	8102	10188	13222
0.2	2386	2676	3026	3452	3978	4640	5486	6596	8092	10174

Selected sample size

To calculate the expected cases in the intervention group and the control group, the function *cpower* was run with the following parameters:

- accrual time 2 years;
- follow-up time 3 years;
- aplha 0.05;
- power 0.8;
- 5% yearly rate of stroke/death/intracranial bleeding for AF-/OAK-;
- 19% AF proportions in the intervention group;
- 6% AF proportions in the control group.

The result of *cpower* is:

```
Accrual duration: 2 y  Minimum follow-up: 3 y
```

Biostatistics
CORE FACILITY

```
Total sample size: 2718

Alpha- 0.05

3-year Mortalities
     Control Intervention
   0.1901558    0.1544025

Hazard Rates
     Control Intervention
  0.07030447   0.05590393

Probabilities of an Event During Study
     Control Intervention
   0.2445141    0.1999611

Expected Number of Events
     Control Intervention
       332.3        271.7

Hazard ratio: 0.7951689
Standard deviation of log hazard ratio: 0.08178795
    Power
0.8002227
```

In the first two years, you have to enroll uniformly over time 1359 patients in the control group and 1359 in the intervention group. After the first two years, you have to follow them up for another three years. At least five years after the start of the study, you must check the observed cases in the control group and in the intervention group. If the study did not reach 333 cases in the control group and 272 in the intervention group, the follow-up can be extended until these are reached. This minimum number of cases guarantees a power of 80%.

For peer review only - http://bmjopen.bmj.com/site/about/guidelines.xhtml

References

1. Diener HC, Connolly SJ, Ezekowitz MD, Wallentin L, Reilly PA, Yang S, Xavier D, Di Pasquale G and Yusuf S. Dabigatran compared with warfarin in patients with atrial fibrillation and previous transient ischaemic attack or stroke: a subgroup analysis of the RE-LY trial. *Lancet Neurol*. 2010;9:1157-63.

2. Diener HC, Eikelboom J, Connolly SJ, Joyner CD, Hart RG, Lip GY, O'Donnell M, Hohnloser SH, Hankey GJ, Shestakovska O, Yusuf S, Committee AS and Investigators. Apixaban versus aspirin in patients with atrial fibrillation and previous stroke or transient ischaemic attack: a predefined subgroup analysis from AVERROES, a randomised trial. *Lancet Neurol*. 2012;11:225-31.

3. Amarenco P, Lavallée PC, Labreuche J, Albers GW, Bornstein NM, Canhão P, Caplan LR, Donnan GA, Ferro JM, Hennerici MG, Molina C, Rothwell PM, Sissani L, Školoudík D, Steg PG, Touboul PJ, Uchiyama S, Vicaut É and Wong LK. One-Year Risk of Stroke after Transient Ischemic Attack or Minor Stroke. *N Engl J Med*. 2016;374:1533-42.

4. Andersen SD, Gorst-Rasmussen A, Lip GY, Bach FW and Larsen TB. Recurrent Stroke: The Value of the CHA2DS2VASc Score and the Essen Stroke Risk Score in a Nationwide Stroke Cohort. *Stroke*. 2015;46:2491-7.

5. Wachter R, Groschel K, Gelbrich G, Hamann GF, Kermer P, Liman J, Seegers J, Wasser K, Schulte A, Jurries F, Messerschmid A, Behnke N, Groschel S, Uphaus T, Grings A, Ibis T, Klimpe S, Wagner-Heck M, Arnold M, Protsenko E, Heuschmann PU, Conen D, Weber-Kruger M, Find AFI and Coordinators. Holter-electrocardiogram-monitoring in patients with acute ischaemic stroke (Find-AFRANDOMISED): an open-label randomised controlled trial. *Lancet Neurol*. 2017;16:282-290.

6. Hankey GJ, Patel MR, Stevens SR, Becker RC, Breithardt G, Carolei A, Diener HC, Donnan GA, Halperin JL, Mahaffey KW, Mas JL, Massaro A, Norrving B, Nessel CC, Paolini JF, Roine RO, Singer DE, Wong L, Califf RM, Fox KA, Hacke W and Investigators RASC. Rivaroxaban compared with warfarin in patients with atrial fibrillation and previous stroke or transient ischaemic attack: a subgroup analysis of ROCKET AF. *Lancet Neurol*. 2012;11:315-22.

For peer review only - http://bmjopen.bmj.com/site/about/guidelines.xhtml

7. Easton JD, Lopes RD, Bahit MC, Wojdyla DM, Granger CB, Wallentin L, Alings M, Goto S, Lewis BS, Rosenqvist M, Hanna M, Mohan P, Alexander JH, Diener HC, Committees A and Investigators. Apixaban compared with warfarin in patients with atrial fibrillation and previous stroke or transient ischaemic attack: a subgroup analysis of the ARISTOTLE trial. *Lancet Neurol*. 2012;11:503-11.

8. Rost NS, Giugliano RP, Ruff CT, Murphy SA, Crompton AE, Norden AD, Silverman S, Singhal AB, Nicolau JC, SomaRaju B, Mercuri MF, Antman EM, Braunwald E and Investigators EA-T. Outcomes With Edoxaban Versus Warfarin in Patients With Previous Cerebrovascular Events: Findings From ENGAGE AF-TIMI 48 (Effective Anticoagulation With Factor Xa Next Generation in Atrial Fibrillation-Thrombolysis in Myocardial Infarction 48). *Stroke*. 2016;47:2075-82.

9. Lip GY, Hunter TD, Quiroz ME, Ziegler PD and Turakhia MP. Atrial Fibrillation Diagnosis Timing, Ambulatory ECG Monitoring Utilization, and Risk of Recurrent Stroke. *Circ Cardiovasc Qual Outcomes*. 2017;10.

10. Jabaudon D, Sztajzel J, Sievert K, Landis T and Sztajzel R. Usefulness of ambulatory 7-day ECG monitoring for the detection of atrial fibrillation and flutter after acute stroke and transient ischemic attack. *Stroke*. 2004;35:1647-51.

11. Shafqat S, Kelly PJ and Furie KL. Holter monitoring in the diagnosis of stroke mechanism. *Intern Med J*. 2004;34:305-9.

12. Alhadramy O, Jeerakathil TJ, Majumdar SR, Najjar E, Choy J and Saqqur M. Prevalence and predictors of paroxysmal atrial fibrillation on Holter monitor in patients with stroke or transient ischemic attack. *Stroke*. 2010;41:2596-600.

13. Rizos T, Rasch C, Jenetzky E, Hametner C, Kathoefer S, Reinhardt R, Hepp T, Hacke W and Veltkamp R. Detection of paroxysmal atrial fibrillation in acute stroke patients. *Cerebrovasc Dis*. 2010;30:410-7.

14. Doliwa Sobocinski P, Anggardh Rooth E, Frykman Kull V, von Arbin M, Wallen H and Rosenqvist M. Improved screening for silent atrial fibrillation after ischaemic stroke. *Europace*. 2012;14:1112-6.

For peer review only - http://bmjopen.bmj.com/site/about/guidelines.xhtml

15. Lazzaro MA, Krishnan K and Prabhakaran S. Detection of atrial fibrillation with concurrent holter monitoring and continuous cardiac telemetry following ischemic stroke and transient ischemic attack. *J Stroke Cerebrovasc Dis*. 2012;21:89-93.

16. Gumbinger C, Krumsdorf U, Veltkamp R, Hacke W and Ringleb P. Continuous monitoring versus HOLTER ECG for detection of atrial fibrillation in patients with stroke. *Eur J Neurol*. 2012;19:253-7.

17. Suissa L, Lachaud S and Mahagne MH. Optimal Timing and Duration of Continuous Electrocardiographic Monitoring for Detecting Atrial Fibrillation in Stroke Patients. *J Stroke Cerebrovasc Dis*. 2012.

18. Thakkar S and Bagarhatta R. Detection of paroxysmal atrial fibrillation or flutter in patients with acute ischemic stroke or transient ischemic attack by Holter monitoring. *Indian Heart J*. 2014;66:188-92.

19. Bansil S and Karim H. Detection of atrial fibrillation in patients with acute stroke. *J Stroke Cerebrovasc Dis*. 2004;13:12-5.

20. Barthelemy JC, Feasson-Gerard S, Garnier P, Gaspoz JM, Da Costa A, Michel D and Roche F. Automatic cardiac event recorders reveal paroxysmal atrial fibrillation after unexplained strokes or transient ischemic attacks. *Ann Noninvasive Electrocardiol*. 2003;8:194-9.

21. Wallmann D, Tuller D, Wustmann K, Meier P, Isenegger J, Arnold M, Mattle HP and Delacretaz E. Frequent atrial premature beats predict paroxysmal atrial fibrillation in stroke patients: an opportunity for a new diagnostic strategy. *Stroke*. 2007;38:2292-4.

22. Tayal AH, Tian M, Kelly KM, Jones SC, Wright DG, Singh D, Jarouse J, Brillman J, Murali S and Gupta R. Atrial fibrillation detected by mobile cardiac outpatient telemetry in cryptogenic TIA or stroke. *Neurology*. 2008;71:1696-701.

23. Elijovich L, Josephson SA, Fung GL and Smith WS. Intermittent atrial fibrillation may account for a large proportion of otherwise cryptogenic stroke: a study of 30-day cardiac event monitors. *J Stroke Cerebrovasc Dis*. 2009;18:185-9.

For peer review only - http://bmjopen.bmj.com/site/about/guidelines.xhtml

24. Bhatt A, Majid A, Razak A, Kassab M, Hussain S and Safdar A. Predictors of occult paroxysmal atrial fibrillation in cryptogenic strokes detected by long-term noninvasive cardiac monitoring. *Stroke Res Treat*. 2011;2011:172074.

25. Flint AC, Banki NM, Ren X, Rao VA and Go AS. Detection of paroxysmal atrial fibrillation by 30-day event monitoring in cryptogenic ischemic stroke: the Stroke and Monitoring for PAF in Real Time (SMART) Registry. *Stroke*. 2012;43:2788-90.

26. Higgins P, MacFarlane PW, Dawson J, McInnes GT, Langhorne P and Lees KR. Noninvasive cardiac event monitoring to detect atrial fibrillation after ischemic stroke: a randomized, controlled trial. *Stroke*. 2013;44:2525-31.

27. Miller DJ, Khan MA, Schultz LR, Simpson JR, Katramados AM, Russman AN and Mitsias PD. Outpatient cardiac telemetry detects a high rate of atrial fibrillation in cryptogenic stroke. *J Neurol Sci*. 2013;324:57-61.

28. Gladstone DJ, Spring M, Dorian P, Panzov V, Thorpe KE, Hall J, Vaid H, O'Donnell M, Laupacis A, Cote R, Sharma M, Blakely JA, Shuaib A, Hachinski V, Coutts SB, Sahlas DJ, Teal P, Yip S, Spence JD, Buck B, Verreault S, Casaubon LK, Penn A, Selchen D, Jin A, Howse D, Mehdiratta M, Boyle K, Aviv R, Kapral MK and Mamdani M. Atrial fibrillation in patients with cryptogenic stroke. *N Engl J Med*. 2014;370:2467-77.

29. Sebasigari D, Merkler A, Guo Y, Gialdini G, Kummer B, Hemendinger M, Song C, Chu A, Cutting S, Silver B, Elkind MSV, Kamel H, Furie KL and Yaghi S. Biomarkers of Atrial Cardiopathy and Atrial Fibrillation Detection on Mobile Outpatient Continuous Telemetry After Embolic Stroke of Undetermined Source. *J Stroke Cerebrovasc Dis*. 2017;26:1249-1253.

For peer review only

SPIRIT 2013 Checklist: Recommended items to address in a clinical trial protocol and related documents*

Section/item	ItemNo	Description	Comment/finished
**Administrative information**			
Title	1	Descriptive title identifying the study design, population, interventions, and, if applicable, trial acronym	Page 1
Trial registration	2a	Trial identifier and registry name. If not yet registered, name of intended registry	Page 4
	2b	All items from the World Health Organization Trial Registration Data Set	
Protocol version	3	Date and version identifier	Page 4
Funding	4	Sources and types of financial, material, and other support	Page 24
Roles and responsibilities	5a	Names, affiliations, and roles of protocol contributors	Page 24
	5b	Name and contact information for the trial sponsor	Page 4

For peer review only - http://bmjopen.bmj.com/site/about/guidelines.xhtml

	5c	Role of study sponsor and funders, if any, in study design; collection, management, analysis, and interpretation of data; writing of the report; and the decision to submit the report for publication, including whether they will have ultimate authority over any of these activities	
	5d	Composition, roles, and responsibilities of the coordinating centre, steering committee, endpoint adjudication committee, data management team, and other individuals or groups overseeing the trial, if applicable (see Item 21a for data monitoring committee)	
**Introduction**			
Background and rationale	6a	Description of research question and justification for undertaking the trial, including summary of relevant studies (published and unpublished) examining benefits and harms for each intervention	Page 6-8
	6b	Explanation for choice of comparators	Page 8
Objectives	7	Specific objectives or hypotheses	
Trial design	8	Description of trial design including type of trial (eg, parallel group, crossover, factorial, single group), allocation ratio, and framework (eg, superiority, equivalence, noninferiority, exploratory)	Page 10

For peer review only - http://bmjopen.bmj.com/site/about/guidelines.xhtml

Methods: Participants, interventions, and outcomes

Study setting	9	Description of study settings (eg, community clinic, academic hospital) and list of countries where data will be collected. Reference to where list of study sites can be obtained	Page 10
Eligibility criteria	10	Inclusion and exclusion criteria for participants. If applicable, eligibility criteria for study centres and individuals who will perform the interventions (eg, surgeons, psychotherapists)	Page 10-11
Interventions	11a	Interventions for each group with sufficient detail to allow replication, including how and when they will be administered	Page 11-14
	11b	Criteria for discontinuing or modifying allocated interventions for a given trial participant (eg, drug dose change in response to harms, participant request, or improving/worsening disease)	Page 11
	11c	Strategies to improve adherence to intervention protocols, and any procedures for monitoring adherence (eg, drug tablet return, laboratory tests)	
	11d	Relevant concomitant care and interventions that are permitted or prohibited during the trial	

For peer review only - http://bmjopen.bmj.com/site/about/guidelines.xhtml

Outcomes	12	Primary, secondary, and other outcomes, including the specific measurement variable (eg, systolic blood pressure), analysis metric (eg, change from baseline, final value, time to event), method of aggregation (eg, median, proportion), and time point for each outcome. Explanation of the clinical relevance of chosen efficacy and harm outcomes is strongly recommended	Page 15
Participant timeline	13	Time schedule of enrolment, interventions (including any run-ins and washouts), assessments, and visits for participants. A schematic diagram is highly recommended (see Figure)	Page 15
Sample size	14	Estimated number of participants needed to achieve study objectives and how it was determined, including clinical and statistical assumptions supporting any sample size calculations	Page 16-17
Recruitment	15	Strategies for achieving adequate participant enrolment to reach target sample size	Page 17

Methods: Assignment of interventions (for controlled trials)

Allocation:

For peer review only - http://bmjopen.bmj.com/site/about/guidelines.xhtml

Sequence generation	16a	Method of generating the allocation sequence (eg, computer-generated random numbers), and list of any factors for stratification. To reduce predictability of a random sequence, details of any planned restriction (eg, blocking) should be provided in a separate document that is unavailable to those who enrol participants or assign interventions	Page 17
Allocation concealment mechanism	16b	Mechanism of implementing the allocation sequence (eg, central telephone; sequentially numbered, opaque, sealed envelopes), describing any steps to conceal the sequence until interventions are assigned	
Implementation	16c	Who will generate the allocation sequence, who will enrol participants, and who will assign participants to interventions	
Blinding (masking)	17a	Who will be blinded after assignment to interventions (eg, trial participants, care providers, outcome assessors, data analysts), and how	Page 17
	17b	If blinded, circumstances under which unblinding is permissible, and procedure for revealing a participant's allocated intervention during the trial	

Methods: Data collection, management, and analysis

Data collection methods	18a	Plans for assessment and collection of outcome, baseline, and other trial data, including any related processes to promote data quality (eg, duplicate measurements, training of assessors) and a description of study instruments (eg, questionnaires, laboratory tests) along with their reliability and validity, if known. Reference to where data collection forms can be found, if not in the protocol	Page 17-19
	18b	Plans to promote participant retention and complete follow-up, including list of any outcome data to be collected for participants who discontinue or deviate from intervention protocols	Page 19
Data management	19	Plans for data entry, coding, security, and storage, including any related processes to promote data quality (eg, double data entry; range checks for data values). Reference to where details of data management procedures can be found, if not in the protocol	
Statistical methods	20a	Statistical methods for analysing primary and secondary outcomes. Reference to where other details of the statistical analysis plan can be found, if not in the protocol	Page 19-20
	20b	Methods for any additional analyses (eg, subgroup and adjusted analyses)	NA

	20c	Definition of analysis population relating to protocol non-adherence (eg, as randomised analysis), and any statistical methods to handle missing data (eg, multiple imputation)	
**Methods: Monitoring**			
Data monitoring	21a	Composition of data monitoring committee (DMC); summary of its role and reporting structure; statement of whether it is independent from the sponsor and competing interests; and reference to where further details about its charter can be found, if not in the protocol. Alternatively, an explanation of why a DMC is not needed	Page 20
	21b	Description of any interim analyses and stopping guidelines, including who will have access to these interim results and make the final decision to terminate the trial	N/A
Harms	22	Plans for collecting, assessing, reporting, and managing solicited and spontaneously reported adverse events and other unintended effects of trial interventions or trial conduct	Page 21
Auditing	23	Frequency and procedures for auditing trial conduct, if any, and whether the process will be independent from investigators and the sponsor	Page 21
**Ethics and dissemination**			

For peer review only - http://bmjopen.bmj.com/site/about/guidelines.xhtml

Research ethics approval	24	Plans for seeking research ethics committee/institutional review board (REC/IRB) approval	Page 21
Protocol amendments	25	Plans for communicating important protocol modifications (eg, changes to eligibility criteria, outcomes, analyses) to relevant parties (eg, investigators, REC/IRBs, trial participants, trial registries, journals, regulators)	Page 23
Consent or assent	26a	Who will obtain informed consent or assent from potential trial participants or authorised surrogates, and how (see Item 32)	Page 23
	26b	Additional consent provisions for collection and use of participant data and biological specimens in ancillary studies, if applicable	
Confidentiality	27	How personal information about potential and enrolled participants will be collected, shared, and maintained in order to protect confidentiality before, during, and after the trial	Page 23
Declaration of interests	28	Financial and other competing interests for principal investigators for the overall trial and each study site	Page 24
Access to data	29	Statement of who will have access to the final trial dataset, and disclosure of contractual agreements that limit such access for investigators	Page 25

For peer review only - http://bmjopen.bmj.com/site/about/guidelines.xhtml

For peer review only

Ancillary and post-trial care	30	Provisions, if any, for ancillary and post-trial care, and for compensation to those who suffer harm from trial participation	N/A
Dissemination policy	31a	Plans for investigators and sponsor to communicate trial results to participants, healthcare professionals, the public, and other relevant groups (eg, via publication, reporting in results databases, or other data sharing arrangements), including any publication restrictions	
	31b	Authorship eligibility guidelines and any intended use of professional writers	
	31c	Plans, if any, for granting public access to the full protocol, participant-level dataset, and statistical code	
**Appendices**			
Informed consent materials	32	Model consent form and other related documentation given to participants and authorised surrogates	
Biological specimens	33	Plans for collection, laboratory evaluation, and storage of biological specimens for genetic or molecular analysis in the current trial and for future use in ancillary studies, if applicable	

For peer review only - http://bmjopen.bmj.com/site/about/guidelines.xhtml

*It is strongly recommended that this checklist be read in conjunction with the SPIRIT 2013 Explanation & Elaboration for important clarification on the items. Amendments to the protocol should be tracked and dated. The SPIRIT checklist is copyrighted by the SPIRIT Group under the Creative Commons "Attribution-NonCommercial-NoDerivs 3.0 Unported" license.

For peer review only

For peer review only - http://bmjopen.bmj.com/site/about/guidelines.xhtml

BMJ Open

A multicenter, national, investigator-initiated, randomised, parallel-group, register based superiority trial to compare extended ECG monitoring versus standard ECG monitoring in elderly patients with ischemic stroke or transient ischemic attack and the effect on stroke, death and intracerebral bleeding – AF SPICE.

Journal:	*BMJ Open*
Manuscript ID	bmjopen-2023-073470.R1
Article Type:	Protocol
Date Submitted by the Author:	19-Sep-2023
Complete List of Authors:	Engdahl, Johan; Karolinska Institutet, Department of Clinical Sciences, Danderyd Hospital<br>Straat, Kajsa; Karolinska Institutet, Department of clinical sciences, Danderyd Hospital<br>Isaksson, Eva; Karolinska Institutet, Department of clinical sciences, Danderyd Hospital<br>Rooth, Elisabeth; Karolinska Institutet, Department of clinical Sciences, Danderyd Hospital<br>Svennberg, Emma; Karolinska Institutet, Department of Medicine, Huddinge, Karolinska University Hospital,<br>Norrving, Bo; Lund University, Section of Neurology, Department of Clinical Sciences<br>Euler, Mia; Orebro universitet, School of Medicine, Department of Neurology<br>Hellqvist, Kjersti; Alingsas lasarett, Department of Medicine<br>Gu, Weigang; Karolinska Institutet, Department of Clinical Sciences, South Hospital<br>Ström, Jakob; Orebro universitet, School of Medicine, Department of Neurology<br>Själander, Sara; Umeå University, Department of Public Health and Clinical Medicine<br>Eriksson, Marie; Umeå University, Department of Statistics, USBE<br>Åsberg, Signild; Uppsala University, Department of Medical Sciences<br>Wester, Per; Umeå University, Department of Public Health and Clinical Medicine; Karolinska Institutet, Dept of Clinical Sciences, Danderyd Hospital
**Primary Subject Heading**:	Cardiovascular medicine
Secondary Subject Heading:	Neurology
Keywords:	Stroke < NEUROLOGY, Cardiology < INTERNAL MEDICINE, Thromboembolism < CARDIOLOGY

A multicenter, national, investigator-initiated, randomised, parallel-group, register based superiority trial to compare extended ECG monitoring versus standard ECG monitoring in elderly patients with ischemic stroke or transient ischemic attack and the effect on stroke, death and intracerebral bleeding – AF SPICE.

Johan Engdahl[1], Kajsa Strååt[1], Eva Isaksson[1], Elisabeth Rooth[1], Emma Svennberg[2], Bo Norrving[3], Mia von Euler[4], Kjersti Hellqvist[5], Weigang Gu[6], Jakob O Ström[4], Sara Själander[7], Marie Eriksson[8], Signild Åsberg[9]*, Per Wester[1,7]*

*Shared senior authorship

1. Karolinska Institutet, Dept of Clinical Sciences, Danderyd Hospital, Stockholm, Sweden.

2. Karolinska Institutet, Dept of Medicine, Huddinge, Karolinska University Hospital, Stockholm, Sweden.

3. Section of Neurology, Department of Clinical Sciences, Lund University, Lund, Sweden.

4. School of Medicine, Department of Neurology and Rehabilitation, Örebro University, Örebro, Sweden

5. Department of Medicine, Alingsås Hospital, Alingsås, Sweden.

6. Karolinska Institutet, Dept of Clinical Sciences, South Hospital, Stockholm, Sweden.

7. Department of Public Health and Clinical Medicine, Umeå University, Umeå, Sweden.

8. Dept of Statistics, USBE, Umeå University, Umeå, Sweden

9. Dept of Medical Sciences, Uppsala University, Uppsala, Sweden

Corresponding author:

Johan Engdahl

Department of Cardiology

Danderyd University Hospital

SE-182 88 Stockholm

For peer review only

For peer review only - http://bmjopen.bmj.com/site/about/guidelines.xhtml

29 Sweden

30 Email: johan.engdahl@regionstockholm.se

31 Phone: +46 737 593374

32 Fax: +46 8 7550868

33 Word count: main 3873 (excluding CIS); abstract 279

For peer review only

For peer review only - http://bmjopen.bmj.com/site/about/guidelines.xhtml

ABSTRACT

Introduction

Atrial fibrillation (AF) is a major risk factor for ischemic stroke and transient ischemic attack (TIA), and AF detection can be challenged by asymptomatic and paroxysmal presentation. Long-term ECG monitoring after ischemic stroke or TIA is recommended by all major societies in cardiology and cerebrovascular medicine as a secondary prophylactic measure. However, data on stroke reduction are lacking, and the recommendations show significant diversity.

Methods and analysis

AF SPICE is a multicenter, national, investigator-initiated, randomised, parallel-group, register-based trial comparing extended ECG monitoring versus standard ECG monitoring in patients admitted with ischemic stroke or TIA, with a composite endpoint of stroke, all-cause-mortality and intracerebral bleeding. Patients aged ≥70 years, without previous AF will be randomised 1:1 to control (standard ECG monitoring) or intervention (extended ECG monitoring). In the control arm, patients will undergo 48h±24h (i.e. range 24-72 hours) of continuous ECG monitoring according to national recommendations. In the intervention arm, patients will undergo 14+14 days of continuous ECG monitoring three months apart using an ECG patch device, which will provide an easy-accessed, well-tolerated 14-days continuous ECG recording. All ECG patch recordings will be read in a core facility. In case of AF detection, oral anticoagulation will be recommended if not contraindicated. A pilot phase has been concluded in 2022 which will transcend into the main trial during 2023-2026,

For peer review only - http://bmjopen.bmj.com/site/about/guidelines.xhtml

including approximately 30 stroke units. The sample size is calculated to 3,262 patients. The primary outcome will be collected from register data during a 36-month follow-up.

Ethics and dissemination

Ethical approval has been provided by the Swedish Ethical Review Authority, reference 2021-02770. The trial will be conducted according to the ethical principles of the declaration of Helsinki and national regulatory standards.

Trial Registration

ClinicalTrials.gov NCT05134454

Trial protocol version 2.0

Sponsor contact information:

Karolinska Institutet

Department of clinical sciences

Danderyd Hospital

SE-18288 Danderyd

Sweden

For peer review only

STRENGTHS AND LIMITATIONS OF THE TRIAL

- First register-based randomised clinical trial on secondary preventive ECG screening post stroke/TIA with stroke and all-cause mortality endpoints
- Participants included from a national network of stroke units and central adjudication of ECG recordings in the intervention arm
- Robust inclusion criteria mirroring clinical real-world population, age at least 70 years for inclusion
- The Swedish stroke register (Riksstroke) used for provision of majority of baseline data
- Endpoints from health care registers will not be further adjudicated

For peer review only

INTRODUCTION

Ischemic stroke is one of the leading causes of mortality worldwide, and a major cause behind permanent disability in adults(1). Despite implementation of novel treatment strategies, morbidity and mortality remain significant.

Modifiable risk factors for stroke include hypertension, atrial fibrillation (AF), hyperlipidaemia, smoking and diabetes(2, 3). Several of these risk factors are being undetected for a long time since they remain asymptomatic.

During 2018, the stroke incidence in Sweden was 223 per 100 000 in men and 191 per 100 000 in women(4). At three-month follow up post-stroke, 26% were deceased or dependent in activities of daily living(5). (4)The risk for stroke recurrence is highest during the first 3-6 months following admission, and the one-year recurrence risk has been reported to be 3-5%(6, 7).

The societal burden of the disease is significant. Disability with need for long-term care, and in younger patients impaired work ability entails enormous societal costs(8). Strokes due to AF are more severe and have a significantly higher mortality than other stroke cases(9).

AF is the most prevalent clinically arrhythmia with a steeply increasing incidence with advancing age(10). AF is also one of the strongest risk factors for stroke(3). However, the increased stroke risk associated with AF can be markedly reduced by oral anticoagulation (OAC) treatment(11). Unfortunately, AF is paroxysmal and asymptomatic in a significant proportion of stroke patients, leading to lower detection rates, and frequently leaves stroke survivors with an undiagnosed and untreated risk factor and higher risk of stroke recurrence(12). Despite this, no study so far has reported evidence to the benefit of

For peer review only

For peer review only - http://bmjopen.bmj.com/site/about/guidelines.xhtml

electrocardiographic (ECG) AF screening in terms of reduced stroke recurrence and mortality after a stroke event.

(13)Randomised controlled trials (CRYSTAL-AF(14) and EMBRACE(12)) have shown that prolonged ECG-monitoring compared to standard of care (at the time 24 hr-ECG) increased detection of atrial fibrillation in cryptogenic stroke patients.

The FIND-$AF_{randomised}$ trial(15) also showed increased detection of atrial fibrillation and included a broader group of participants suggesting that prolonged ECG-monitoring should be used in all ischemic stroke patients.

The MonDAFIS study(16) was powered to evaluate the proportion of anticoagulation therapy 12 months after index stroke. Standard of care was compared to prolonged in hospital ECG-monitoring (maximum 7 days). Atrial fibrillation detection was increased in the intervention group, but this had no significant effect on the rate of anticoagulation therapy at 12 months.

AF screening post an ischemic stroke event is currently recommended by major international societies (Table 1)(17-23). The recommendations vary markedly with respect to method of screening and duration of screening. There are however a body of data on the proportion of AF detection using different monitoring strategies(24).

The development of ECG modalities and clinical practice for AF screening post ischemic stroke and TIA have evolved towards longer and more resource-demanding investigations as noted in table 1, despite the lack of data on clinical endpoints like stroke. Diversity within recommendations have promoted unequal care even within countries and regions. With this background, there is an urgent need for a randomised trial to investigate the efficacy of secondary prophylactic AF screening following ischemic stroke.

For peer review only - http://bmjopen.bmj.com/site/about/guidelines.xhtml

Choice of comparators

In some of the recommendations on AF-screening post-ischemic stroke from international societies, a basic monitoring duration of 24 hours of continuous ECG is recommended(18, 20). In the recommendations from the Swedish Board of Health and Welfare, continuous ECG monitoring of 24-48 hours is recommended as the baseline investigation for ischemic stroke patients, and a large share of stroke units in Sweden adhere to this recommendation.

METHODS AND ANALYSIS

Primary Objective

To determine if extended continuous ECG screening (14+14 days) is superior to standard ECG screening (48 ±24 hours i.e. range 24-72 hours) in reducing the risk of the combined endpoint of ischemic stroke, intracerebral bleeding and all-cause mortality in elderly patients admitted to hospital for ischemic stroke or TIA.

Key Secondary Objectives

The key secondary objectives are to estimate the treatment effect in primary outcome, and determine if there are any differences between extended and standard ECG screening for:

- Ischemic stroke
- All-cause mortality
- Intracranial bleeding
- Major bleeding

For peer review only - http://bmjopen.bmj.com/site/about/guidelines.xhtml

- Myocardial infarction
- Pacemaker implantation
- Cost effectiveness

Other Secondary Objectives

- To study association between age and AF detection
- To study the proportion of other relevant arrhythmias detected at ECG screening
- To study predictors for AF detection
- To study signal time and signal quality achieved at ECG screening
- To study long-term adherence to OAC treatment
- To study feasibility of patients application of the second ECG patch
- To study Patient Reported Experience (PREM), collected 3 months after inclusion in the main trial
- Per protocol analysis of the primary outcome
- Win ratio analysis

Trial design

AF SPICE is designed as a multicenter, national, investigator-initiated, randomised, parallel-group, register based superiority trial to compare extended ECG monitoring versus standard ECG Monitoring in Elderly Patients admitted with ischemic stroke or TIA with the combined endpoint of recurrent ischemic stroke, all-cause mortality and intracerebral bleeding (Figure 1).

For peer review only

For peer review only - http://bmjopen.bmj.com/site/about/guidelines.xhtml

Study setting

AF SPICE will recruit participants among admissions for ischemic stroke or TIA in stroke units in Swedish hospitals. Inclusion is also possible from non-stroke units given that the patient is admitted for ischemic stroke or TIA and identified by the site's local investigator or delegate.

A pilot trial commenced in January 2022 and is now concluded, converting into the main study in 2023. The pilot trial evaluated inclusion rates, feasibility and adherence to ECG recorders and the database with randomisation system.

Eligibility criteria

Inclusion criteria

1. Patients aged ≥ 70 years admitted to a stroke unit with a diagnosis of ischemic stroke or TIA within 14 days from inclusion. Stroke is clinically defined as an acute onset of focal neurological deficit of presumed vascular origin lasting for ≥ 24 hours or resulting in death. Stroke is further categorized as ischemic or as intracerebral or subarachnoid haemorrhage based on computed tomography (CT) and/or magnetic resonance imaging (MRI) of the brain. Transient ischemic attack (TIA) is defined as a sudden, focal neurological deficit of presumed vascular origin with transient symptoms (< 24 hours)(25). Only patients with ischemic stroke and TIA are eligible for inclusion.
2. Signed informed consent.

For peer review only - http://bmjopen.bmj.com/site/about/guidelines.xhtml

Exclusion criteria

1. Previously diagnosed AF
2. Contraindication to oral anticoagulant treatment
3. Indication for anticoagulant treatment other than AF.
4. Patients with pacemaker, implantable cardioverter defibrillator or implantable loop recorder.
5. Patients who, according to the investigator, will not be able to comply with the trial protocol.
6. Previous participation in the AF SPICE trial

The rationale for the age cut-off of 70 years in AF SPICE is partly due to the higher AF yield in elderly patients, as well as the increased benefit for oral anticoagulation treatment in elderly patient in the case of AF detection. Notably, 74% of stroke patients in Sweden are aged 70 years of above, making the study population quite representative of the future implementation. The recruitment window of 14 days is intended to give a margin for the occurrence of patient transfer between centres for thrombectomy, holidays and uncertainty about eligibility. Patients with a cardiac implantable device are excluded since their very long-lasting continuous heart rhythm monitoring will make them biased with a lower probability of AF detection on a surface ECG recording. Further, ventricular pacing will make reading of the patch ECG difficult due to the lack of pacing annotations.

Withdrawal of patients

A patient can withdraw from the trial at any time, at the discretion of the patient, or by the investigator if deemed medically indicated. If a participant refrain from completing extended

For peer review only

206 ECG recording protocol, he/she will remain included in the trial and in the intention-to-
207 screen data analysis. A withdrawal of consent means that no further trial data will be
208 collected, however already collected data will not be erased.

209 Patient and public involvement

210 Patient or public involvement is planned but not yet in place in this trial.

211 Interventions

212 Extended ECG Investigation

213 Participants randomised to extended ECG investigation will first undergo the standard 0-
214 2 days of ECG monitoring, before receiving a continuous one-lead ECG-recording using
215 the BioTel ePatch (https://www.biotel.se). The BioTel ePatch is a class IIa CE-marked
216 Holter ECG recorder using a patch for skin adhesion.

217 The ECG patch will be applied by health care professionals at the stroke unit (visit 1) at
218 randomisation.

219 After completion of the 14-day ECG recording, the device should be returned using a
220 pre-paid envelope. Following arrival at BioTel, the ECG data will be uploaded to a web-
221 accessed interpretation service (Cardiologs, https://cardiologs.com/) within 1 working
222 day from arrival.

223 During visit 2, participants not diagnosed with AF will have the second ePatch attached
224 by health care professionals in connection to a scheduled follow-up visit or attached by

For peer review only - http://bmjopen.bmj.com/site/about/guidelines.xhtml

the participant/next of kin. The rationale for the second ePatch recording is to increase AF yield, even though the second recording will reasonably identify fewer cases of AF.

The ECG recordings will be scrutinised by a core ECG reading team led by JE. All events in the ECG readings will be manually overread.

Procedures for handling of arrhythmias detected on extended ECG recording

Atrial fibrillation is defined according to the current definition of the ESC AF guidelines(20), i.e. at least 30 seconds with irregularly irregular R-R intervals, absence of distinct repeating p-waves and irregular atrial activations. When this is met, the local investigator will be notified within one working day by phone and email by the ECG reading team.

If a participant is diagnosed with other arrhythmias with a prognostic implication as defined in table 2, the local investigator will be notified by email within three working days. However, due to the high prevalence of ventricular arrhythmias during 14 days of continuous ECG in this population, a selection based on team discussion is made for VTs. If a more rapid action is deemed necessary by the ECG reading team, appropriate measures will be undertaken. When no significant arrhythmias are noted, the local investigator will be notified within one week from ECG upload.

Procedures for poor signal quality and short recordings in extended ECG

If the signal quality of the extended ECG recording is deemed to be insufficient, the participant should be offered another patch ECG recording. If this second recording has

For peer review only

insufficient signal quality or if it is not possible to arrange for another patch ECG recording, a traditional Holter recording, or ECG event recording could be used. However, it is of importance for the power of the trial to achieve as long ECG recordings as possible in the intervention group.

At least 7 days of ECG recording should be yielded from ECG patch in visit one. If less than 7 days are recorded, another recording should be offered. The same minimum ECG duration of 7 days applies for ECG recording at visit two.

If a participant randomised to intervention arm with a negative ECG recording (i.e., no atrial fibrillation detected) suffers a recurrent ischemic stroke within 2 months from visit one, the second ECG patch recording (visit 2) may be started earlier than 2 months to the discretion of the local investigator.

Oral anticoagulation initiation following AF detection in extended ECG recording

Patients diagnosed with AF during the long-term ECG recordings initiated in connection to randomisation should be informed by the local investigator and offered treatment with oral anticoagulation if there are no contraindications, based on the stroke risk of at least CHA_2DS_2-VASc 3 points in randomised patients.

Treatment with OAC and its long-term follow-up will be handled within standard care and according to national guidelines.

When indicated, OAC treatment should be initiated within five working days from the reception of long-term ECG results. Long-term follow-up of OAC treatment will be using the national drug prescription registry.

For peer review only

For peer review only - http://bmjopen.bmj.com/site/about/guidelines.xhtml

All other treatments and investigations will be at the discretion of the local investigator.

Standard care

Participants randomised to standard care should undergo 48 ±24 hours of continuous ECG monitoring, using inpatient ECG telemetry monitoring or Holter recording. The duration of the monitoring should be recorded by the site and noted in the electronic case report form (REDCap, Vanderbilt University, TN, United States).

The trial will not collect any data from the standard recordings apart from its duration, and containment of AF. ECG recordings of newly diagnosed AF in the standard care arm will be collected and stored in the trial.

Outcomes

Primary endpoint

Difference between the two arms in a combined endpoint of recurrent ischemic stroke, intracerebral bleeding, and all-cause mortality during at least 36 months of follow-up.

Secondary endpoints

- Individual components of the primary endpoint
- Major bleeding including Intracranial bleeding
- Myocardial infarction
- Pacemaker implantation
- Anticoagulation treatment

For peer review only - http://bmjopen.bmj.com/site/about/guidelines.xhtml

- Atrial fibrillation prevalence
- Adherence to long-term ECG recording
- Incidence of other prognostic arrhythmia during long-term ECG recording

Participant timeline intervention arm

Participants will be screened for consent during admission for ischemic stroke or TIA, within 14 days from admission. ECG investigation of 0-48 hours prior to randomisation is allowed. Extended ECG recording is initiated during admission for ischemic stroke/TIA and the participant will return the device via mail. The second extended ECG recording should be initiated at follow-up visit or by mailing the device to the participants for self-application. Following completion of second 14-day ECG recording, no further personal visits are made in the trial.

Participant timeline standard arm

Starting or completing the ECG recording in the standard arm following discharge is allowed. Following completion of the ECG investigation initiated in the standard arm, no further personal visits are made in the trial.

Sample size

The sample size was calculated based on the primary outcome.

The event rate of recurrent ischemic stroke, intracerebral bleeding and all-cause mortality was estimated in patients admitted for ischemic stroke or TIA. Event rate was estimated in

For peer review only

patients with AF with and without OAC treatment and in patients without AF. A complete list of studies used for estimations in the sample size calculation is reported in the appendix. Most studies used in this estimation reported data from patient cohorts with a lower age than in the AF SPICE trial. This could lead to an underestimation of event rates. Recent publications on AF status and outcomes in stroke patients in the projected age group is not available at the time of trial start.

For the estimations of AF detection proportions, available studies on short-term (48 ±24 hours) ECG recording following stroke and corresponding studies on long-term (14 days) recordings were used. A list of the AF detection studies available for this estimation is reported in the appendix.

The assumptions for the sample size calculation are reported in table 3. The complete sample size calculation is provided in the appendix. For the yearly risk of the composite endpoint, we estimate 17% in patients with AF and no OAC treatment, 7,3% in patients with AF and OAC treatment and 5% risk in patients without AF. We further estimate an AF detection proportion of 6% in the control group and 19% after two ECG recordings in the intervention group, and 13% with untreated AF in the control group, resulting in a sample size of 2718 for three years of fixed follow-up time after inclusion. The estimated AF detection in the control and intervention groups include cases detected during follow-up. Adding 20% for participant dropouts and treatment discontinuation, a total sample size of 3262 is needed, corresponding to 1631 in each arm based on three years follow-up, 80% power and double-sided significance level of 5%. The study is powered to detect a 9% reduction of the composite endpoint in the group with extended ECG screening.

For peer review only

For peer review only - http://bmjopen.bmj.com/site/about/guidelines.xhtml

Recruitment

Patients will be screened and included during ischemic stroke or TIA admissions in stroke units. Participating centres report at least 200 stroke/TIA admissions yearly. With an anticipated participation of 30 sites, they need to randomise 0.6 patients weekly during three years of inclusion to fulfil the recruitment target of 3262 participants.

Allocation

Participants will be randomly assigned to either control or intervention group with a 1:1 allocation as per a computer-generated randomisation schedule stratified by site in the online REDCap database. Randomisation will be performed immediately following the collection of necessary baseline data.

Blinding

Due to the nature of the intervention, neither participants nor staff will be blinded to allocation.

Data collection methods primary Outcome (Table 4a)

Primary endpoint variables are collected from Swedish health care registers as specified in Table 4a.

Data collection methods Secondary and exploratory outcomes (Table 4b)

Variables collected for secondary and exploratory outcomes are reported in table 4b.

For peer review only - http://bmjopen.bmj.com/site/about/guidelines.xhtml

Baseline data entered at inclusion all participants (supplementary table)

Data entered at baseline in all participants are listed in supplementary table. The remaining baseline variables will be collected from the Riksstroke register[5].

Baseline data entered in control and intervention arms (Appendix)

Data entered at baseline in standard and interventions arms are listed in the Appendix.

Baseline register data from Riksstroke register (Appendix)

Baseline data from Riksstroke are listed in the appendix.

ECG data (Appendix)

There are different ECG variables for the control and intervention arms.

1. In the control arm, the local investigator or delegate will report if ECG was recorded using ECG telemetry and/or Holter recording, the duration of each recording, and whether AF was diagnosed.

2. In the intervention arm, ECG data from patch recordings will be stored in the patch hardware and returned by the participant by mail. Data from ECG patch hardware will be uploaded by the patch provider into the Cardiologs database. ECG patch data will be scrutinized by the core reading team which also enter ECG data manually into the REDCap database.

For the second ECG patch recording initiated 2-4 months following randomisation, data will be collected on whether the patch is applied within health care or by the participant.

For peer review only

For peer review only - http://bmjopen.bmj.com/site/about/guidelines.xhtml

371 Cost Effectiveness

372 The cost effectiveness analysis will be based on a Markov cohort model where prevalence of
373 AF, use of oral anticoagulants, clinical events and all-cause mortality will be collected from
374 the AF SPICE study. The cost for clinical events, age-specific utilities and stroke death will be
375 collected from the literature. Number of gained life-years, quality-adjusted life years and
376 cost for the screening process will be calculated.

377

378 Retention

379 At the majority of including sites, the second visit for the intervention group coincides with
380 an outpatient follow-up visit after stroke, which will give the participant motivation for
381 attending the visit. Patients receive no reimbursement for participation.

382

383 Statistical Methods

384 The main analysis of outcome will be based on time to event (endpoint) and performed
385 according to the intention to treat principle. The cumulative incidence of the combined
386 endpoint and its individual components will be presented for each treatment group
387 (standard and extended ECG).

388

389 For the primary analysis, of the combined endpoint, the Log-rank test will be used to test for
390 treatment group differences. Cox proportional hazard regression will be used to estimate

For peer review only

the hazard ratios with 95% confidence intervals, and to analyse associations with atrial fibrillation incidence and/or effect modification by age, comorbidity, biometrics, and supraventricular activity.

The secondary analysis, of each individual components of the primary endpoint, will account for competing risks. We will use cause-specific models (where the rate of each individual endpoint is based on patients who have not yet experienced any of the endpoints) and subdistributional hazard models (where the rate is based on patients who have not yet experience the endpoint under study). Other analysis, including baseline characteristics, will be presented by proportions (binary variables) and means (continuous variables) with 95% confidence intervals in each ECG group. Corresponding group comparisons will be carried out by $\chi 2$-tests and t-tests. An outcome with a p-value < 0.05 will be considered statistically significant.

Data Monitoring Committee

An independent Data Safety and Monitoring Board (DSMB) will be appointed at the start of the main trial, i.e., after the pilot phase. The DSMB will have a consulting role to the steering committee. The DSMB will monitor and annually provide statements on:

1. Cumulative inclusion rate, balance in baseline characteristics, duration and yield of ECG recordings during the inclusion phase.

2. Event rate in the standard and intervention group annually.

For peer review only

For peer review only - http://bmjopen.bmj.com/site/about/guidelines.xhtml

Harms

All diagnostic tools used in the trial are approved by regulatory bodies. Any adverse events noted in connection to investigations or treatment should be reported to authorities according to practice.

Auditing

A monitoring plan for the trial has been established, with the following areas:

1. Investigator qualifications, delegation log
2. Written consent forms, accuracy, and completeness.
3. Subject eligibility
4. Source data, accuracy and completeness
5. CRF entries in REDCap, unverified and incomplete entries by site
6. Independent reader will scrutinize 5-10% of ECG reports in intervention arm.

ETHICS AND DISSEMINATION

Independent ethics committee

Ethical approval has been provided by the Swedish Ethical Review Authority (Reference 2021-02770). Before entering the trial, verbal and written information is given to the patient and informed consent form is signed. The trial will be conducted according to the ethical principles of the declaration of Helsinki(26) and national regulatory standards.

For peer review only

For peer review only - http://bmjopen.bmj.com/site/about/guidelines.xhtml

Ethical considerations

The optimal duration of the ECG investigation following ischemic stroke/TIA is not known.(20)

The ethical issues in this trial are partly connected to the knowledge gap of ECG monitoring duration and the clinical practice that has evolved. Several stroke units are applying ECG monitoring protocols with longer duration than the recommended standard. Randomising patients to a monitoring protocol with shorter ECG screening duration be regarded as lowering investigation ambitions. Since the long-term efficacy of different ECG monitoring strategies is unknown, the randomisation to one of the arms in this trial is considered ethically balanced against the option of continuing using monitoring strategies with unknown efficacy and unknown cost effectiveness.

Protocol amendments

Important modifications to the protocol will be summarized consecutively in the protocol and disseminated to investigators. Major protocol changes, i.e. changes in eligibility criteria or patient information texts are subjected to ethical review.

Consent

Trained research nurses or investigators will introduce the trial. Patients will receive written information. Research nurses or investigators will discuss the trial in light of the written information given, and patients will have an opportunity to discuss the information and ask questions.

For peer review only

For peer review only - http://bmjopen.bmj.com/site/about/guidelines.xhtml

In patients with aphasia or inability to write following stroke, it seems reasonable to collect informed consent from next of kin, i.e. a spouse or other close relative if some other mode of communication is established between the patient and the next of kin.

Confidentiality

At the trial sites, all log lists containing personal identification data will be stored securely. All trial data will be stored in a REDCap database, accessible by multifactor authentication. All actions in the REDCap database are logged. Data in the ECG reading system Cardiologs are pseudonymized and accessible with password.

For peer review only

For peer review only - http://bmjopen.bmj.com/site/about/guidelines.xhtml

AUTHORS´ CONTRIBUTIONS

JE conceived of the trial and drafted the protocol. PW, KS, SÅ, ME and EI made major contributions to trial design. ER, ES, BN, MvE, KH, WG, JBS and SS made contributions to trial design. All authors approved of the final manuscript. AF SPICE is conducted in collaboration with the Swedish Stroke Register, Riksstroke.

FUNDING STATEMENT

AF SPICE is funded by grants from the Swedish Research Council, the Swedish Heart & Lung Foundation, the Swedish Stroke Foundation, and the Stockholm Region (ALF). The funding sources had no role in the design of this trial and will not have any role during its execution, analyses, interpretation of the data, or decision to submit results.

ACKNOWLEDGEMENT

We are grateful to Ida Hed Myrberg and Letizia Orsini at Karolinska Institutet Biostatistics Core Facility for assistance with the power calculation.

COMPETEING INTEREST STATEMENT

JE has received consultant or lecture fees from Roche Diagnostics, Pfizer, Bristol Myers Squibb, Boehringer Ingelheim, Piotrode and Philips. Research grants from the Swedish Research Council, The Swedish Heart&Lung Foundation, The Swedish Innovation Agency and

For peer review only

The Stockholm Region. KS, EI report no disclosures. ES reports consultant or lecture fees from Bayer, Bristol-Myers Squibb, Pfizer, Boehringer-Ingelheim, Johnson & Johnson, Merck Sharp & Dome. Research grants from the Swedish Research Council, The Swedish Heart&Lung Foundation, Åke Wibergs Foundation and CIMED. BN reports Data Safety Monitoring Board fees from Astra Zeneca. MvE is the chairman of Riksstroke, the Swedish Stroke Register. JOS reports no disclosures. SÅ reports lecture fees paid to the institution from Bristol-Myers Squibb, Pfizer, Boehringer-Ingelheim, and is a member of the steering committee of Riksstroke, the Swedish Stroke Register. PW reports consulting fees, fee as a Clinical Events Committee member and unrestricted grants from Abbott. PW is a member of the steering committee of Riksstroke, the Swedish Stroke Register.

Access to data

The investigators will have access to the final dataset without any restricions.

For peer review only

REFERENCES

1. Global, regional, and national burden of stroke and its risk factors, 1990-2019: a systematic analysis for the Global Burden of Disease Study 2019. Lancet Neurol. 2021;20(10):795-820.

2. Hankey GJ. Stroke. Lancet. 2017;389(10069):641-54.

3. O'Donnell MJ, Chin SL, Rangarajan S, Xavier D, Liu L, Zhang H, Rao-Melacini P, Zhang X, Pais P, Agapay S, Lopez-Jaramillo P, Damasceno A, Langhorne P, McQueen MJ, Rosengren A, Dehghan M, Hankey GJ, Dans AL, Elsayed A, Avezum A, Mondo C, Diener HC, Ryglewicz D, Czlonkowska A, Pogosova N, Weimar C, Iqbal R, Diaz R, Yusoff K, Yusufali A, Oguz A, Wang X, Penaherrera E, Lanas F, Ogah OS, Ogunniyi A, Iversen HK, Malaga G, Rumboldt Z, Oveisgharan S, Al Hussain F, Magazi D, Nilanont Y, Ferguson J, Pare G, Yusuf S. Global and regional effects of potentially modifiable risk factors associated with acute stroke in 32 countries (INTERSTROKE): a case-control study. Lancet. 2016;388(10046):761-75.

4. Eriksson M, Åsberg S, Sunnerhagen KS, von Euler M. Sex Differences in Stroke Care and Outcome 2005-2018: Observations From the Swedish Stroke Register. Stroke. 2021:Strokeaha120033893.

For peer review only

5. Riksstroke. Riksstroke english summary 2021 2023 [Available from: https://www.riksstroke.org/annual-reports-and-graphs/.

6. Andersen SD, Gorst-Rasmussen A, Lip GY, Bach FW, Larsen TB. Recurrent Stroke: The Value of the CHA2DS2VASc Score and the Essen Stroke Risk Score in a Nationwide Stroke Cohort. Stroke. 2015;46(9):2491-7.

7. Khanevski AN, Bjerkreim AT, Novotny V, Naess H, Thomassen L, Logallo N, Kvistad CE. Recurrent ischemic stroke: Incidence, predictors, and impact on mortality. Acta Neurol Scand. 2019;140(1):3-8.

8. Mukundan G, Seidenwurm DJ. Economic and Societal Aspects of Stroke Management. Neuroimaging Clin N Am. 2018;28(4):683-9.

9. Henninger N, Goddeau RP, Jr., Karmarkar A, Helenius J, McManus DD. Atrial Fibrillation Is Associated With a Worse 90-Day Outcome Than Other Cardioembolic Stroke Subtypes. Stroke. 2016;47(6):1486-92.

10. Bjorck S, Palaszewski B, Friberg L, Bergfeldt L. Atrial fibrillation, stroke risk, and warfarin therapy revisited: a population-based study. Stroke. 2013;44(11):3103-8.

For peer review only

For peer review only - http://bmjopen.bmj.com/site/about/guidelines.xhtml

11. Hart RG, Pearce LA, Aguilar MI. Meta-analysis: antithrombotic therapy to prevent stroke in patients who have nonvalvular atrial fibrillation. Ann Intern Med. 2007;146(12):857-67.

12. Gladstone DJ, Spring M, Dorian P, Panzov V, Thorpe KE, Hall J, Vaid H, O'Donnell M, Laupacis A, Cote R, Sharma M, Blakely JA, Shuaib A, Hachinski V, Coutts SB, Sahlas DJ, Teal P, Yip S, Spence JD, Buck B, Verreault S, Casaubon LK, Penn A, Selchen D, Jin A, Howse D, Mehdiratta M, Boyle K, Aviv R, Kapral MK, Mamdani M. Atrial fibrillation in patients with cryptogenic stroke. N Engl J Med. 2014;370(26):2467-77.

13. Grond M, Jauss M, Hamann G, Stark E, Veltkamp R, Nabavi D, Horn M, Weimar C, Kohrmann M, Wachter R, Rosin L, Kirchhof P. Improved Detection of Silent Atrial Fibrillation Using 72-Hour Holter ECG in Patients With Ischemic Stroke: A Prospective Multicenter Cohort Study. Stroke. 2013;44(12):3357-64.

14. Sanna T, Diener HC, Passman RS, Di Lazzaro V, Bernstein RA, Morillo CA, Rymer MM, Thijs V, Rogers T, Beckers F, Lindborg K, Brachmann J. Cryptogenic stroke and underlying atrial fibrillation. N Engl J Med. 2014;370(26):2478-86.

15. Wachter R, Groschel K, Gelbrich G, Hamann GF, Kermer P, Liman J, Seegers J, Wasser K, Schulte A, Jurries F, Messerschmid A, Behnke N, Groschel S, Uphaus T, Grings A, Ibis T, Klimpe S, Wagner-Heck M, Arnold M, Protsenko E, Heuschmann PU, Conen D, Weber-Kruger M, Find AFI, Coordinators. Holter-electrocardiogram-monitoring in patients with acute

For peer review only

ischaemic stroke (Find-AFRANDOMISED): an open-label randomised controlled trial. Lancet Neurol. 2017;16(4):282-90.

16. Haeusler KG, Kirchhof P, Kunze C, Tütüncü S, Fiessler C, Malsch C, Olma MC, Jawad-Ul-Qamar M, Krämer M, Wachter R, Michalski D, Kraft A, Rizos T, Gröschel K, Thomalla G, Nabavi DG, Röther J, Laufs U, Veltkamp R, Heuschmann PU, Endres M. Systematic monitoring for detection of atrial fibrillation in patients with acute ischaemic stroke (MonDAFIS): a randomised, open-label, multicentre study. Lancet Neurol. 2021;20(6):426-36.

17. NICE AF post stroke Guidelines 2023 [Available from: https://www.nice.org.uk/guidance/dg41/.

18. Andrade JG, Aguilar M, Atzema C, Bell A, Cairns JA, Cheung CC, Cox JL, Dorian P, Gladstone DJ, Healey JS, Khairy P, Leblanc K, McMurtry MS, Mitchell LB, Nair GM, Nattel S, Parkash R, Pilote L, Sandhu RK, Sarrazin JF, Sharma M, Skanes AC, Talajic M, Tsang TSM, Verma A, Verma S, Whitlock R, Wyse DG, Macle L. The 2020 Canadian Cardiovascular Society/Canadian Heart Rhythm Society Comprehensive Guidelines for the Management of Atrial Fibrillation. Can J Cardiol. 2020;36(12):1847-948.

19. Brieger D, Amerena J, Attia J, Bajorek B, Chan KH, Connell C, Freedman B, Ferguson C, Hall T, Haqqani H, Hendriks J, Hespe C, Hung J, Kalman JM, Sanders P, Worthington J, Yan TD, Zwar N. National Heart Foundation of Australia and the Cardiac Society of Australia and New

For peer review only

For peer review only - http://bmjopen.bmj.com/site/about/guidelines.xhtml

Zealand: Australian Clinical Guidelines for the Diagnosis and Management of Atrial Fibrillation 2018. Heart, lung & circulation. 2018;27(10):1209-66.

20. Hindricks G, Potpara T, Dagres N, Arbelo E, Bax JJ, Blomström-Lundqvist C, Boriani G, Castella M, Dan GA, Dilaveris PE, Fauchier L, Filippatos G, Kalman JM, La Meir M, Lane DA, Lebeau JP, Lettino M, Lip GYH, Pinto FJ, Thomas GN, Valgimigli M, Van Gelder IC, Van Putte BP, Watkins CL. 2020 ESC Guidelines for the diagnosis and management of atrial fibrillation developed in collaboration with the European Association for Cardio-Thoracic Surgery (EACTS): The Task Force for the diagnosis and management of atrial fibrillation of the European Society of Cardiology (ESC) Developed with the special contribution of the European Heart Rhythm Association (EHRA) of the ESC. Eur Heart J. 2021;42(5):373-498.

21. Kleindorfer DO, Towfighi A, Chaturvedi S, Cockroft KM, Gutierrez J, Lombardi-Hill D, Kamel H, Kernan WN, Kittner SJ, Leira EC, Lennon O, Meschia JF, Nguyen TN, Pollak PM, Santangeli P, Sharrief AZ, Smith SC, Jr., Turan TN, Williams LS. 2021 Guideline for the Prevention of Stroke in Patients With Stroke and Transient Ischemic Attack: A Guideline From the American Heart Association/American Stroke Association. Stroke. 2021;52(7):e364-e467.

22. Physicians Rco. National guideline for stroke London2016 [Fifth edition:[Available from: https://www.strokeaudit.org/Guideline/Full-Guideline.aspx.

For peer review only - http://bmjopen.bmj.com/site/about/guidelines.xhtml

23. Rubiera M, Aires A, Antonenko K, Lémeret S, Nolte CH, Putaala J, Schnabel RB, Tuladhar AM, Werring DJ, Zeraatkar D, Paciaroni M. European Stroke Organisation (ESO) guideline on screening for subclinical atrial fibrillation after stroke or transient ischaemic attack of undetermined origin. Eur Stroke J. 2022;7(3):Vi.

24. Schnabel RB, Haeusler KG, Healey JS, Freedman B, Boriani G, Brachmann J, Brandes A, Bustamante A, Casadei B, Crijns H, Doehner W, Engstrom G, Fauchier L, Friberg L, Gladstone DJ, Glotzer TV, Goto S, Hankey GJ, Harbison JA, Hobbs FDR, Johnson LSB, Kamel H, Kirchhof P, Korompoki E, Krieger DW, Lip GYH, Lochen ML, Mairesse GH, Montaner J, Neubeck L, Ntaios G, Piccini JP, Potpara TS, Quinn TJ, Reiffel JA, Ribeiro ALP, Rienstra M, Rosenqvist M, Sakis T, Sinner MF, Svendsen JH, Van Gelder IC, Wachter R, Wijeratne T, Yan B. Searching for Atrial Fibrillation Poststroke: A White Paper of the AF-SCREEN International Collaboration. Circulation. 2019;140(22):1834-50.

25. Aho K, Harmsen P, Hatano S, Marquardsen J, Smirnov VE, Strasser T. Cerebrovascular disease in the community: results of a WHO collaborative study. Bull World Health Organ. 1980;58(1):113-30.

26. World Medical Association Declaration of Helsinki: ethical principles for medical research involving human subjects. Jama. 2013;310(20):2191-4.

For peer review only

For peer review only - http://bmjopen.bmj.com/site/about/guidelines.xhtml

Table 1 ECG screening post stroke society recommendations

Society	Recommendation Summary	Year	Ref
European Stroke Organisation	In adult patients with ischaemic stroke or TIA of undetermined origin, we recommend a prolonged cardiac monitoring instead of standard 24 h monitoring to increase the detection of subclinical AF.<br><br>In adult patients with ischaemic stroke or TIA of **undetermined origin**, we recommend longer duration of cardiac rhythm monitoring of more than 48 h and if feasible with ILR to increase the detection of subclinical AF.	2022	21
American Heart Association/American Stroke Association	In patients suspected of having a stroke or TIA, an ECG is recommended to screen for atrial fibrillation (AF) and atrial flutter and to assess for other concomitant cardiac conditions.<br><br>In patients with **cryptogenic stroke** who do not have a contraindication to anticoagulation, long-term rhythm monitoring with mobile cardiac outpatient telemetry, implantable loop recorder, or other approach is reasonable to detect intermittent AF.	2021	19
European Society of Cardiology	In patients with acute ischaemic stroke or TIA and without previously known AF, monitoring for AF is recommended using a short-term ECG recording for at least the first 24 h, followed by continuous ECG monitoring for at least 72 h whenever possible<br>In selected stroke patients without previous known AF, additional monitoring using long-term non-invasive ECG monitors or insertable cardiac monitors should be considered, to detect AF.	2020	18
Swedish board of health and welfare	Health care should provide long-term ECG for 24-48 h.<br><br>long-term ECG for more than 48 h duration using Holter recording or inpatient telemetry ECG in selected cases.	2020	
NICE	Reveal LINQ is recommended as an option to help to detect atrial fibrillation after **cryptogenic stroke** only if:<br><br>non-invasive electrocardiogram (ECG) monitoring has been done **and** a cardiac arrhythmic cause of stroke is still suspected.	2020	15
Canadian Cardiovascular Society/Canadian Heart Rhythm Society	We recommend at least 24 hours of ambulatory ECG monitoring to identify AF in patients with **nonlacunar ESUS**We suggest additional monitoring for AF detection (eg, prolonged external loop recorder or implantable cardiac monitoring, where available) be performed for selected older patients with **nonlacunar ESUS** in whom AF is suspected but unproven	2020	16
National Heart Foundation of Australia and the Cardiac Society of Australia and New Zealand	Screening for Atrial Fibrillation in Patients with **Embolic Stroke of Uncertain Source** Recommendation: For patients with **ESUS**, longer term ECG monitoring (external or implantable) should be used.	2018	17
Royal College of Physicians	People with ischaemic stroke or TIA who would be eligible for secondary prevention treatment for atrial fibrillation (anticoagulation or left atrial appendage device closure) should undergo a period of prolonged (at least 12 hours) cardiac monitoring. People with ischaemic stroke or TIA who would be eligible for secondary prevention treatment for atrial fibrillation and in whom no other cause of stroke has been found should be considered for more prolonged ECG monitoring (24 hours or longer), particularly if they have a pattern of cerebral ischaemia on brain imaging suggestive of cardioembolism.	2016	20

For peer review only

Table 2. Prognostic ECG findings on extended ECG recording which should be communicated to the local investigator.

Ventricular tachycardia
Excessive ventricular extrasystoles (> 5%)
Pause > 3 seconds
AV-block type II and III
Bradycardia < 40/minute

For peer review only

For peer review only - http://bmjopen.bmj.com/site/about/guidelines.xhtml

**Endpoint* risk in patients with AF and OAC treatment**	**7.3%**
**Endpoint risk in patients with AF and no OAC treatment**	**17%**
**Endpoint risk in patients without AF**	**5%**
**AF detection proportion in standard arm****	**6%**
**AF detection proportion in intervention arm****	**19%**

*Endpoint including recurrent stroke, all-cause mortality, and intracerebral bleeding.**Including AF detection during follow-up.

Table 3. Event rates and atrial fibrillation detection rates used in sample size calculation.

For peer review only

For peer review only - http://bmjopen.bmj.com/site/about/guidelines.xhtml

Table 4a Primary outcome

Variable	Coding ICD-10	Source	Validity
Ischemic stroke	I63	Riksstroke	High
Stroke not specified	I64	Riksstroke	High
All-cause mortality	N/A	National register of death	Very high
Intracerebral bleeding (spontaneous)	I61	Riksstroke	High

Table 4b Secondary outcome

<table>
<tr><th>Variable</th><th colspan="2">Coding ICD-10</th><th>Source</th></tr>
<tr><td>Myocardial infarction</td><td colspan="2">I21, I22, I25.2</td><td rowspan="9">National inpatient register, Riksstroke</td></tr>
<tr><td rowspan="5">Major bleeding</td><td>Gastrointestinal bleeding</td><td>K226, K250, K252, K254, K256, K260, K262, K264, K266, K270, K272, K274, K276, K280, K282, K284, K286, K290, K625, K661, K920, K921, K922, I850, I983</td></tr>
<tr><td>Urogenital bleeding</td><td>N02, R319, N939, N950, N501A</td></tr>
<tr><td>Other bleeding</td><td>H113, H313, H356, H431, H450, H922, I312, J942, M250, R04, R58, T810, D500, D629, T792</td></tr>
<tr><td>Sub-, epidural and subarachnoid haemorrhages (incl traumatic)</td><td>I60, I61, I62, S064, S065, S066</td></tr>
<tr><td>Procedure codes for transfusion</td><td>DR029, DR033, Z513</td></tr>
<tr><td>Atrial fibrillation/flutter</td><td colspan="2">I48</td></tr>
<tr><td>Pacemaker implantation</td><td colspan="2">FPE00, FPE10, FPE20, FPE26</td></tr>
<tr><td>OAC and antiplatelet treatment, NSAID</td><td colspan="2">B01A, N02BA, M01A</td><td>National drug dispense register</td></tr>
</table>

For peer review only

For peer review only

For peer review only - http://bmjopen.bmj.com/site/about/guidelines.xhtml

Figure legend

Figure 1. Overview of study design AF SPICE.

For peer review only

For peer review only - http://bmjopen.bmj.com/site/about/guidelines.xhtml

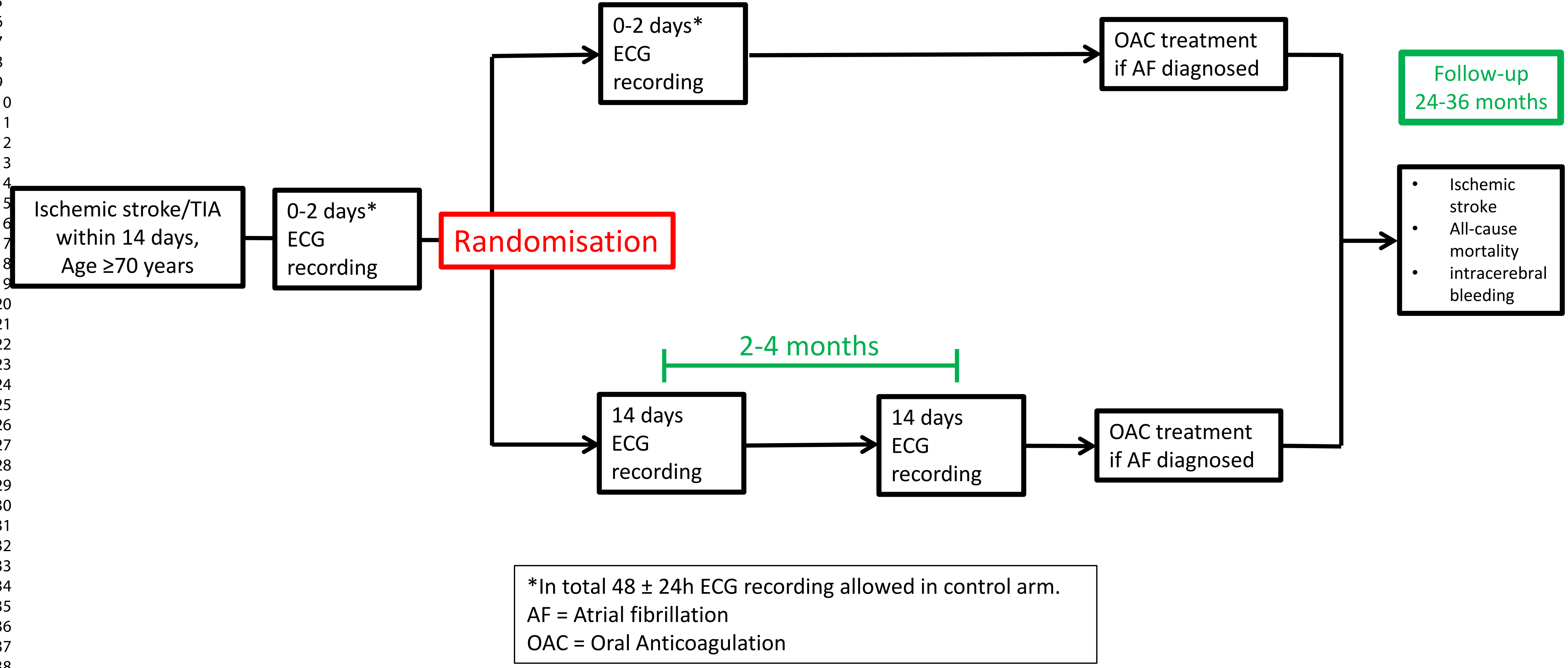
Ischemic stroke/TIA within 14 days, Age ≥70 years
0-2 days* ECG recording
Randomisation
0-2 days* ECG recording
OAC treatment if AF diagnosed
2-4 months
14 days ECG recording
14 days ECG recording
OAC treatment if AF diagnosed
Follow-up 24-36 months
Ischemic stroke
All-cause mortality
intracerebral bleeding
*In total 48 ± 24h ECG recording allowed in control arm.
AF = Atrial fibrillation
OAC = Oral Anticoagulation

Supplementary Appendix

Table of contents

Trials in event rates power calculation 2
Appendix table Trials in ECG detection rate power calculation 3
Baseline data entered at inclusion 4
Baseline data collected from Riksstroke register 5
ECG Data collected by local investigator in standard arm 6
Data collected by local investigator in intervention arm 6
Sample size calculation 13
Patient consent form 16

For peer review only

Trials in event rates power calculation

	AF+/OAC+			AF+/OAC-			AF-/OAC-		
	Rate	Mean Age	Reference	Rate	Mean Age	Reference	Rate	Mean Age	Reference
**Stroke**	2,23%	70,2	Diener[1] 2010 D110	8,3%	71,7	Diener[2] 2012	5,40%	73	Netland el al 2019
	1,91%	70,8	Diener[1] 2010 D150				5,10%	66	Amarenco[3] 2016
	2,53%	70,4	Diener[1] 2010 W				3,60%	70,1	Andersen[4] 2015
	2,39%	71,7	Diener[2] 2012 A				5,40%		Wachter[5] 2017
	2,79%	71	Hankey[6] 2012 R						
	2,96%	71	Hankey[6] 2012 W						
	2,26%	70,1	Easton[7] 2012 A						
	3,17%	70,1	Easton[7] 2012 W						
	2,44%		Rost[8] 2016 E						
**All-cause mortality**	3,24%	70,2	Diener[1] 2010 D110	7,9%	71,7	Diener[2] 2012	10,70%	73	Netland el al 2019
	4,39%	70,8	Diener[1] 2010 D150				1,80%	66	Amarenco[3] 2016
	4,58%	70,4	Diener[1] 2010 W				10,50%	70,1	Andersen[4] 2015
	5,78%	71,7	Diener[2] 2012 A				4,80%		Wachter[5] 2017
	4,40%	71	Hankey[6] 2012 R				10,60%		Lip[9] 2017
	4,54%	71	Hankey[6] 2012 W						
	4,22%	70,1	Easton[7] 2012 A						
	4,77%	70,1	Easton[7] 2012 W						
	4,35%		Rost[8] 2016 E						
**Intracerebral bleeding**	0,25%	70,2	Diener[1] 2010 D110	1,6%	71,7	Diener[2] 2012	0,40%	66	Amarenco[3] 2016
	0,53%	70,8	Diener[1] 2010 D150						
	1,28%	70,4	Diener[1] 2010 W						
	1,17%	71,7	Diener[2] 2012 A						
	0,59%	71	Hankey[6] 2012 R						
	0,80%	71	Hankey[6] 2012 W						
	0,55%	70,1	Easton[7] 2012 A						
	1,49%	70,1	Easton[7] 2012 W						
	0,62%		Rost[8] 2016 E						
**Mortal bleeding**	0,26%	71	Hankey[6] 2012 R						
	0,49%	71	Hankey[6] 2012 W						

AF = Atrial Fibrillation OAC = Oral Anticoagulant

For peer review only - http://bmjopen.bmj.com/site/about/guidelines.xhtml

Appendix table Trials in ECG detection rate power calculation

Publication Short-term external ECG after stroke/TIA	Detection rate (%)
Jabaudon[10] 2004	5.0
Shafqat[11] 2004	2.4
Alhadramy[12] 2010	2.5
Rizos[13] 2010	2.5
Doliwa-Sobocinski[14] 2012	2.0
Lazarro[15] 2012	6.0
Gumbinger[16] 2012	1.0
Suissa[17] 2012	2.3
Thakkar[18] 2014	5.8
Bansil[19] 2004	4.7

Publication Long-term external ECG after stroke/TIA	Detection rate (%)
Barthelemy[20] 2003	14.3
Jabadoun[10] 2004	5.7
Wallman[21] 2007	14.2
Tayal[22] 2008	5.0
Elijiovich[23] 2009	20.0
Bhatt[24] 2011	24.2
Flint[25] 2012	11.0
Higgins[26] 2013	8.0
Miller[27] 2013	17.3
Gladstone[28] 2014	16.1
Sebasigari[29] 2017	11.7
Kaura[30] 2019	16.3

For peer review only

Baseline data entered at inclusion

Variable	Unit
Record ID (automatically generated)	
Sex	Male/Female
Date of birth	Date
Social Security Number	Special format
Date of Stroke/TIA	Date
Age	Years
Height	cm
Weight	kg
Hemoglobin	g/l
Estimated GFR	ml/min
Site identification	Choice from list
Consent Date	Date
Randomization date	Date

For peer review only

For peer review only - http://bmjopen.bmj.com/site/about/guidelines.xhtml

Baseline data collected from Riksstroke register

Reporting Hospital	Statin treatment on admission
Date of symptom onset	Aspirin treatment on admission
Time of symptom onset	Clopidogrel treatment on admission
Level of consciousness at admission	
NIHSS at admission	Dipyridamole treatment on admission
Number of days admitted	Other antiplatelet treatment on admission
Number of days in stroke unit	Hypertension treatment on admission
	Dipyridamole treatment on admission
Diagnosis	Warfarin treatment on admission
Age at admission	Apixaban treatment on admission
Sex	Dabigatran treatment on admission
Level of assistance	Rivaroxaban treatment on admission
Living alone	Edoxaban treatment on admission
Level of dependence	
Assisted dressing	Statin treatment at discharge
Assisted personal hygiene	Aspirin treatment at discharge
Independence in locomotion	Clopidogrel treatment at discharge
	Dipyridamole treatment at discharge
Previously diagnosed AF	Warfarin treatment at discharge
AF diagnosed on arrival	Hypertension treatment at discharge
Previously or newly diagnosed diabetes	Apixaban treatment at discharge
Hypertension (treated) on admission	Dabigatran treatment at discharge
Smoking status	Rivaroxaban treatment at discharge
CT brain scan performed during admission	Reason for withholding OAC treatment
MRI brain scan performed during admission	
Carotide artery ultrasound brain scan performed during admission	Edoxaban treatment at discharge
CT angiography performed during admission	
Longterm ECG (at least 24 h) performed during admission	
Thrombolytic treatment administered	
Type of ward at admission	

For peer review only

For peer review only - http://bmjopen.bmj.com/site/about/guidelines.xhtml

ECG Data collected by local investigator in standard arm

Variable	Unit
Inpatient ECG Telemetry used	Yes/No
Duration of ECG Telemetry	Hours
AF diagnosed during ECG Telemetry	Yes/No
Holter Recording used	Yes/No
Duration of Holter Recording	Hours
AF diagnosed during Holter	Yes/No

For peer review only

Data collected by local investigator in intervention arm

Variable	Unit
ePatch number 1 serial number	Integer
Date epatch number 1 recording start	Date
Additional epatch number 1 serial number	Integer
Site of application additional epatch number 1	Health care/self-application
Date for mailing out additional epatch number 1	Date
Date additional epatch number 1 recording start	Date
Reason for additional epatch	1. epatch detachment<br>2. Red light indicated<br>3. Poor signal quality<br>4. MRI examination

For peer review only - http://bmjopen.bmj.com/site/about/guidelines.xhtml

	5. Other radiology<br>6. Thoracic/neck surgery<br>7. Other reason
Additional epatch number 1 serial number	Integer
Site of application additional epatch number 1	Health care/self-application
Date for mailing out additional epatch number 1	Date
Date additional epatch number 1 recording start	Date
Reason for additional epatch number 1	1. epatch detachment<br>2. Red light indicated<br>3. Poor signal quality<br>4. MRI examination<br>5. Other radiology<br>6. Thoracic/neck surgery<br>7. Other reason
Mode of application epatch number 2	Health care/self-application
Date for mailing of epatch number 2 to participant	Date
Date of application epatch number 2	Date
Epatch number 2 serial number	Integer
First additional epatch number 2 serial number	Integer
Mode of application first additional epatch number 2	Health care/self-application
Date for mailing of first additional epatch number 2 to participant	Date
Date of application of first additional epatch number 2	
Reason for first additional epatch number 2	1. epatch detachment<br>2. Red light indicated<br>3. Poor signal quality<br>4. MRI examination<br>5. Other radiology<br>6. Thoracic/neck surgery<br>7. Other reason

For peer review only - http://bmjopen.bmj.com/site/about/guidelines.xhtml

Second additional epatch number 2 serial number	Integer
Mode of application second additional epatch number 2	Health care/self-application
Date for mailing of second additional epatch number 2 to participant	Date
Date of application of second additional epatch number 2	
Reason for second additional epatch number 2	1. epatch detachment<br>2. Red light indicated<br>3. Poor signal quality<br>4. MRI examination<br>5. Other radiology<br>6. Thoracic/neck surgery<br>7. Other reason
Reason for not recording epatch number 2	1. Diagnosed with AF on epatch number 1<br>2. Participant declines recording<br>3. Participant decline further participation<br>4. Other reason

For peer review only

For peer review only - http://bmjopen.bmj.com/site/about/guidelines.xhtml

ECG Data collected in intervention group

Patch number 1	
**Variable**	**Unit**
Date for ECG data upload in Cardiologs	Date
Date for ECG reading	Date
Date for first additional ECG data upload in Cardiologs	Date
Date for first additional ECG reading	Date
Date for second additional ECG data upload in Cardiologs	Date
Date for second additional ECG reading	Date
Monitoring time	Hours
Analyzable time	Hours
Heart rate max	Bpm
Heart rate min	Bpm
Heart rate mean	Bpm
Proportion supraventricular ectopics	Percent
Proportion ventricular ectopics	Percent
Number of pauses > 2 seconds	Integer
Longest pause	Seconds, one decimal
AV-block II or III	Yes/No
Highest AV-block grade	1. AV-block II type 1<br>2. AV-block II type 2<br>3. AV-block III
Atrial fibrillation	Yes/No
Atrial flutter	Yes/No
Duration to first Atrial fibrillation/flutter	Hours
Atrial fibrillation/flutter subtype	1. Paroxysmal<br>2. Persistant
Atrial fibrillation/ flutter heart rate max	Bpm

For peer review only - http://bmjopen.bmj.com/site/about/guidelines.xhtml

Atrial fibrillation/ flutter heart rate mean	Bpm
Atrial fibrillation/ flutter burden	Percent
Atrial fibrillation/ flutter longest episode	Seconds
Number of Ventricular Tachycardia (VT)	n
Duration longest episode VT	Seconds
Number of Suptraventricular Tachycardia (SVT)	n
Duration longest episode SVT	Seconds
Proportion of noise	Percent
Action triggered by recording	1. Standard statement<br>2. Communication with local PI within 3 working days<br>3. Communication with local PI within one working day<br>4. Further assessment following patch number 2
**Patch number 2**	
**Variable**	**Unit**
Date for ECG data upload in Cardiologs	Date
Date for ECG reading	Date
Date for first additional ECG data upload in Cardiologs	Date
Date for first additional ECG reading	Date
Date for second additional ECG data upload in Cardiologs	Date
Date for second additional ECG reading	Date
Monitoring time	Hours
Analyzable time	Hours
Heart rate max	Bpm
Heart rate min	Bpm

For peer review only - http://bmjopen.bmj.com/site/about/guidelines.xhtml

Heart rate mean	Bpm
Proportion supraventricular ectopics	Percent
Proportion ventricular ectopics	Percent
Number of pauses > 2 seconds	Integer
Longest pause	Seconds, one decimal
AV-block II or III	Yes/No
Highest AV-block grade	1. AV-block II type 1<br>2. AV-block II type 2<br>3. AV-block III
Atrial fibrillation	Yes/No
Atrial flutter	Yes/No
Atrial fibrillation/flutter subtype	1. Paroxysmal<br>2. Persistant
Atrial fibrillation/ flutter heart rate max	Bpm
Atrial fibrillation/ flutter heart rate mean	Bpm
Atrial fibrillation/ flutter burden	Percent
Atrial fibrillation/ flutter longest episode	Seconds
Number of Ventricular Tachycardia (VT)	n
Duration longest episode VT	Seconds
Number of Suptraventricular Tachycardia (SVT)	n
Duration longest episode SVT	Seconds
Proportion of noise	Percent
Action triggered by recording	1. Standard statement<br>2. Communication with local PI within 3 working days<br>3. Communication with local PI within one working day

For peer review only

	4. Further assessment following patch number 2

For peer review only

For peer review only - http://bmjopen.bmj.com/site/about/guidelines.xhtml

Sample size calculation

Biostatistics CORE FACILITY

Sample size calculation AF SPICE

Ida Hed Myrberg & Letizia Orsini

2021-10-08

Background

Sample size calculation for the AF SPICE study; a multicenter randomized register-based trail in Swedish stroke units.

Method

The function *cpower* in R package *Hmisc* is used. This is a function for calculating the power for a Cox Proportional Hazards model or log-rank test comparing two groups. The function assumes an underlying exponential distribution in both groups. To calculate the sample size needed to achieve 80% power, the function *uniroot* is used in combination with *cpower*.

How parameters are defined and calculated

In this section, it is shown how the yearly rate, and the 3-year event rate, of stroke/death/intracranial bleeding in the intervention and control group, respectively, are calculated based on the yearly rates in the three groups AF+/OAK+, AF+/OAK-, and AF-/OAK-, and on the proportions of these three groups in the intervention and control group. The proportion of AF+/OAK+, AF+/OAK-, and AF-/OAK- in the intervention and control group in Table 2 are examples, and in the next two sections they will be varied between 10-20% in the intervention group, and 3-12% in the control group. The yearly rate of stroke/death/intracranial bleeding for AF-/OAK- will be set to 5% and 8.4%, respectively, in two different scenarios.

Table 1 shows the assumed yearly rates of stroke/death/intracranial bleeding for the subgroups AF+/OAK+, AF+/OAK-, AF-/OAK-, while Table 2 shows the proportion of patients in each of the aforementioned subgroups, divided by interention and control.

Table 1: Yearly rate of stroke/death/intracranial bleeding depending on AF and treatment.

AF/treatment	Yearly rate of stroke/death/intracranial bleeding
AF+/OAK+	0.073
AF+/OAK-	0.177
AF-/OAK-	0.084

Table 2: Proportion of patients with AF and treatment in the intervention group and control group, respectively.

AF/treatment	Proportion in intervention group	Proportion in control group
AF+/OAK+	0.10	0.03
AF+/OAK-	0.00	0.07
AF-/OAK-	0.90	0.90

Biostatistics CORE FACILITY

The yearly rate of stroke/death/intracranial in the intervention group is calculated as 0.073 * 0.1 + 0.177 * 0 + 0.084 * 0.9 = 0.0829. The yearly rate of stroke/death/intracranial in the control group is calculated as 0.073 * 0.03 + 0.177 * 0.07 + 0.084 * 0.9 = 0.09018.

Table 3: Yearly rate of stroke/death/intracranial bleeding in the intervention group and control group, respectively.

Group	Yearly rate of stroke/death/intracranial bleeding
Intervention group	0.0829
Control group	0.0902

The minimum follow-up time is assumed to be 3 years, and the recruitment time is set to 2 years.

The 3-year event rate in the control group, denoted *mc*, i.e. the proportion expected to have experienced the event by three years, is needed for the power calculation. The proportion not having experienced the event, i.e. 1-*mc*, is calculated using the Kaplan-Meier method, and the 3-year event rate is then calculated as 1-(1-*mc*)=*mc*. The 3-year event rate in the intervention group, *mi*, is calculated in a similar way. Both *mc* and *mi* are presented in Table 4.

Table 4: 3-year rate of stroke/death/intracranial bleeding in the intervention group and control group, respectively.

Group	3-year rate of stroke/death/intracranial bleeding
Intervention group	0.2287
Control group	0.2469

The percent reduction in the 3-year event rate for the intervention group compared to the control group is given by 1-22.87/24.69=7.38%.

Results: 5% yearly rate of stroke/death/intracranial bleeding for AF-/OAK-

In this section, a yearly event rate of 5% is assumed for the AF-/OAK- group.

The sample size needed is calculated for all combination of AF proportions, ranging from 10-20% in the intervention group, and 3-12% in the control group. Table 5 shows the percent reduction in the 3-year event rate for the intervention group compared to the control group, for different combinations of AF proportions.

Table 5: Percent reduction in the 3-year event rate for the intervention group compared to the control group. Columns: AF proportions in the control group. Rows: AF proportions in the intervention group.

	0.03	0.04	0.05	0.06	0.07	0.08	0.09	0.1	0.11	0.12
0.1	11.57	10.09	8.56	6.97	5.33	3.62	1.85	-	-	-
0.11	12.94	11.52	10.05	8.52	6.94	5.31	3.60	1.84	-	-

Biostatistics CORE FACILITY

	0.03	0.04	0.05	0.06	0.07	0.08	0.09	0.1	0.11	0.12
0.12	14.25	12.89	11.47	10.01	8.49	6.92	5.28	3.59	1.83	-
0.13	15.52	14.20	12.83	11.42	9.96	8.45	6.89	5.26	3.57	1.82
0.14	16.73	15.46	14.14	12.78	11.38	9.92	8.42	6.86	5.24	3.56
0.15	17.89	16.66	15.40	14.09	12.73	11.33	9.88	8.38	6.83	5.21
0.16	19.01	17.82	16.60	15.34	14.03	12.68	11.29	9.84	8.35	6.80
0.17	20.09	18.94	17.76	16.54	15.28	13.98	12.63	11.24	9.80	8.31
0.18	21.12	20.01	18.87	17.69	16.48	15.22	13.92	12.58	11.20	9.76
0.19	22.12	21.05	19.94	18.80	17.63	16.41	15.16	13.87	12.53	11.15
0.2	23.08	22.04	20.97	19.87	18.73	17.56	16.35	15.10	13.82	12.48

Table 6 shows the total sample size needed to achieve 80% power to detect a statistically significant (with alpha=0.05) difference in the 3-year event rates presented in Table 5 between the intervention group and the control group, for different combinations of AF proportions.

Table 6: Total sample size needed for different combinations of AF proportions in the intervention and control group, respectively. Columns: AF proportions in the control group. Rows: AF proportions in the intervention group.

	0.03	0.04	0.05	0.06	0.07	0.08	0.09	0.1	0.11	0.12
0.1	8536	11504	16400	25370	44652	99460	393830	-	-	-
0.11	6628	8572	11552	16470	25480	44848	99900	395590	-	-
0.12	5310	6654	8608	11600	16542	25590	45044	100340	397348	-
0.13	4360	5332	6682	8644	11650	16612	25700	45240	100780	399108
0.14	3652	4378	5352	6710	8680	11698	16682	25810	45436	101220
0.15	3110	3668	4396	5374	6738	8716	11748	16752	25922	45630
0.16	2686	3124	3682	4412	5396	6764	8752	11796	16824	26032
0.17	2346	2696	3136	3696	4430	5418	6792	8788	11846	16894
0.18	2072	2356	2708	3148	3710	4448	5440	6820	8824	11894
0.19	1844	2080	2364	2718	3160	3726	4466	5462	6848	8860
0.2	1654	1850	2086	2374	2728	3172	3740	4484	5484	6876

Results: 8.4% yearly rate of stroke/death/intracranial bleeding for AF-/OAK-

In this section, a yearly event rate of 8.4% is assumed for the AF-/OAK- group.

The sample size needed is calculated for all combination of AF proportions, ranging from 10-20% in the intervention group, and 3-12% in the control group. Table 7 shows the percent reduction in the 3-year event rate for the intervention group compared to the control group, for different combinations of AF proportions.

Table 7: Percent reduction in the 3-year event rate for the intervention group compared to the control group. Columns: AF proportions in the control group. Rows: AF proportions in the intervention group.

	0.03	0.04	0.05	0.06	0.07	0.08	0.09	0.1	0.11	0.12
0.1	7.38	6.40	5.40	4.37	3.32	2.24	1.13	-	-	-
0.11	8.35	7.39	6.41	5.41	4.38	3.32	2.24	1.13	-	-

Biostatistics CORE FACILITY

	0.03	0.04	0.05	0.06	0.07	0.08	0.09	0.1	0.11	0.12
0.12	9.30	8.36	7.40	6.42	5.41	4.38	3.33	2.25	1.14	-
0.13	10.23	9.31	8.37	7.41	6.43	5.42	4.39	3.33	2.25	1.14
0.14	11.14	10.24	9.32	8.38	7.42	6.44	5.43	4.39	3.34	2.25
0.15	12.04	11.16	10.26	9.34	8.40	7.43	6.45	5.44	4.40	3.34
0.16	12.91	12.05	11.17	10.27	9.35	8.41	7.44	6.45	5.44	4.41
0.17	13.78	12.93	12.07	11.19	10.28	9.36	8.42	7.45	6.46	5.45
0.18	14.62	13.79	12.95	12.08	11.20	10.30	9.37	8.43	7.46	6.47
0.19	15.45	14.64	13.81	12.96	12.10	11.22	10.31	9.39	8.44	7.47
0.2	16.26	15.47	14.66	13.83	12.98	12.12	11.23	10.33	9.40	8.45

Table 8 shows the total sample size needed to achieve 80% power to detect a statistically significant (with alpha=0.05) difference in the 3-year event rates presented in Table 5 between the intervention group and the control group, for different combinations of AF proportions.

Table 8: Total sample size needed for different combinations of AF proportions in the intervention and control group, respectively. Columns: AF proportions in the control group. Rows: AF proportions in the intervention group.

	0.03	0.04	0.05	0.06	0.07	0.08	0.09	0.1	0.11	0.12
0.1	13380	18096	25892	40198	71006	158734	630838	-	-	-
0.11	10296	13362	18072	25858	40144	70910	158518	629976	-	-
0.12	8176	10282	13346	18048	25824	40090	70814	158302	629114	-
0.13	6656	8166	10270	13328	18024	25790	40036	70718	158088	628250
0.14	5530	6648	8156	10256	13310	18000	25754	39982	70622	157872
0.15	4670	5522	6640	8144	10242	13292	17976	25720	39928	70526
0.16	3998	4664	5514	6630	8134	10228	13274	17952	25686	39874
0.17	3466	3994	4658	5508	6622	8124	10216	13258	17928	25652
0.18	3034	3460	3988	4652	5500	6614	8112	10202	13240	17904
0.19	2680	3030	3456	3984	4646	5494	6604	8102	10188	13222
0.2	2386	2676	3026	3452	3978	4640	5486	6596	8092	10174

Selected sample size

To calculate the expected cases in the intervention group and the control group, the function *cpower* was run with the following parameters:

- accrual time 2 years;
- follow-up time 3 years;
- aplha 0.05;
- power 0.8;
- 5% yearly rate of stroke/death/intracranial bleeding for AF-/OAK-;
- 19% AF proportions in the intervention group;
- 6% AF proportions in the control group.

The result of *cpower* is:

```
Accrual duration: 2 y  Minimum follow-up: 3 y
```


For peer review only - http://bmjopen.bmj.com/site/about/guidelines.xhtml

Biostatistics
CORE FACILITY

```
Total sample size: 2718

Alpha= 0.05

3-year Mortalities
     Control Intervention
   0.1901558    0.1544025

Hazard Rates
     Control Intervention
  0.07030447   0.05590393

Probabilities of an Event During Study
     Control Intervention
   0.2445141    0.1999611

Expected Number of Events
     Control Intervention
       332.3        271.7

Hazard ratio: 0.7951689
Standard deviation of log hazard ratio: 0.08178795
    Power
0.8002227
```

In the first two years, you have to enroll uniformly over time 1359 patients in the control group and 1359 in the intervention group. After the first two years, you have to follow them up for another three years. At least five years after the start of the study, you must check the observed cases in the control group and in the intervention group. If the study did not reach 333 cases in the control group and 272 in the intervention group, the follow-up can be extended until these are reached. This minimum number of cases guarantees a power of 80%.

For peer review only

For peer review only - http://bmjopen.bmj.com/site/about/guidelines.xhtml

Patient consent form

Patientnummer:_______________

Bilaga 05

Randomiserad jämförelse mellan standardiserad och utökad EKG-undersökning efter stroke eller TIA för att detektera förmaksflimmer och minska risken för stroke - AF SPICE

Forskningspersonsformation och samtyckesformulär

För närvarande pågår i Sverige en vetenskaplig studie som går ut på att studera två olika metoder att upptäcka förmaksflimmer hos personer som drabbats av slaganfall (stroke) eller TIA (övergående stroke). Det är orsaken till att du får denna information och förfrågan. Studien drivs av Karolinska Institutet.

Varför görs detta forskningsprojekt?

Förmaksflimmer är den vanligaste rubbningen i hjärtrytmen och förekomsten ökar med stigande ålder. Tillståndet ger oregelbunden hjärtrytm och symptomen varierar kraftigt, i vissa fall kan man ha förmaksflimmer utan några symptom alls.

Vid förmaksflimmer finns en ökad risk att drabbas av stroke, detta beror på en ökad risk för att det bildas blodproppar i hjärtats förmak, dessa proppar kan sedan följa med blodet till hjärnan och därmed orsaka stroke eller TIA. Risken för stroke vid förmaksflimmer kan reduceras med 60-70% med blodförtunnande behandling. Blodförtunnande behandling innebär också en viss ökning av risken att drabbas av allvarlig blödning.

För de som drabbats av stroke eller TIA är det angeläget att ta reda på om individen har förmaksflimmer. Om så är fallet finns det starka skäl att starta en särskild sorts blodförtunnande behandling, så kallad *antikoagulantia*. Utredning för att hitta förmaksflimmer efter stroke eller TIA görs med olika typer av EKG-undersökningar, ofta över en eller flera dygn. Det finns ännu inte någon forskning som visar på vilken typ av EKG-undersökning som är den bästa efter stroke, vilket också har lett till att praxis varierar. Långa EKG-undersökningar tar också stora resurser i anspråk varför det är angeläget att ta reda på den medicinska nyttan med olika metoder.

I detta forskningsprojekt kommer du att lottas till ett av två alternativ: standardutredning eller utökad utredning. Standardutredningen består av 24-48 timmars EKG-utredning och är den utredning som rekommenderas starkast av Socialstyrelsen. Utökad utredning består av långtids-EKG med så kallat plåster-EKG under 14 dagar vid två tillfällen.

Studien är godkänd av Etikprövningsmyndigheten.

Vad innebär det för mig att delta i studien?

För dig som lottas till **standardutredning** kommer EKG-undersökning genomföras inneliggande på avdelning eller som långtids-EKG med portabel utrustning i hemmet.

För dig som lottas till **utökad utredning** kommer EKG-undersökning med plåster-EKG starta på avdelningen och oftast avslutas efter utskrivning då du sänder tillbaka EKG-plåstret med

Patientnummer:_______________

Bilaga 05

Randomiserad jämförelse mellan standardiserad och utökad EKG-undersökning efter stroke eller TIA för att detektera förmaksflimmer och minska risken för stroke - AF SPICE

ett medsänt svarskuvert. Du kommer också att i samband med ett återbesök påbörja ytterligare en 14-dagars EKG-registrering med plåster-EKG där du sänder tillbaka utrustningen på samma sätt.

Om EKG i någon av undersökningarna visar på förmaksflimmer kommer du utan fördröjning att kontaktas av läkare för att diskutera eventuell ändring av din blodförtunnande behandling.

Därefter görs en långtidsuppföljning under minst tre år där Socialstyrelsens register samt registret Riks-stroke kommer att studeras för att bland annat följa förekomsten av insjuknanden i stroke, förskrivning av proppförebyggande läkemedel och överlevnad. Denna uppföljning kräver ingen aktiv medverkan från dig. De register från Socialstyrelsen som kommer att användas är patientregistret, läkemedelsregistret och dödsorsaksregistret.

Ersättning för deltagande och resor utgår inte.

Vilka obehag/risker medför studien?

Både förmaksflimmer och blodförtunnande behandling gör att man är utsatt för vissa risker, men den riskminskning som åstadkommes med blodförtunnande behandling är i de allra flesta fall flerfalt större än den risk som behandlingen innebär. Denna behandling rekommenderas också i Socialstyrelsens senaste nationella riktlinjer för hjärtsjukvård.

Undersökning med långtids-EKG och plåster-EKG kan i vissa fall ge hudirritation där utrustningen ansluts på huden. Dessa besvär är ofta lindriga och alltid övergående.

Vilka fördelar kan studien innebära för dig?

Ditt deltagande ger en möjlighet att upptäcka och behandla ett tillstånd som ökar risken för ny stroke och TIA, dessutom kan studien ge oss värdefulla kunskaper som även andra kan ha nytta av i framtiden

Hur insamlas personuppgifter och hur hanteras sekretess?

Dina svar och dina resultat kommer att behandlas så att inte obehöriga kan ta del av dem. Ansvarig för dina personuppgifter är Region Stockholm. Enligt EU:s dataskyddsförordning (GDPR 2016/679) har du rätt att kostnadsfritt få ta del av de uppgifter om dig som hanteras i studien, och vid behov få eventuella fel rättade. Du kan också begära att uppgifter om dig raderas samt att behandlingen av dina personuppgifter begränsas. Om du vill ta del av uppgifterna ska du kontakta personuppgiftsombudet på dso.ds@sll.se [alt mailadress för aktuellt site] eller ansvarig forskare. Om du är missnöjd med hur dina personuppgifter behandlas har du rätt att ge in klagomål till Integritetsskyddsmyndigheten, som är tillsynsmyndighet.

For peer review only - http://bmjopen.bmj.com/site/about/guidelines.xhtml

Patientnummer:________________

Bilaga 05

Randomiserad jämförelse mellan standardiserad och utökad EKG-undersökning efter stroke eller TIA för att detektera förmaksflimmer och minska risken för stroke - AF SPICE

Är deltagandet i studien frivilligt?
Deltagandet i denna studie är frivilligt, kostnadsfritt och du har när som helst rätt att avbryta ditt deltagande utan att ange skäl och utan att det påverkar ditt framtida omhändertagande. Kostnad för eventuell läkemedelsbehandling efter ditt deltagande omfattas av sedvanlig läkemedelsförmån.

Kontaktpersoner

Om du vill ha ytterligare information om studien, dina rättigheter som patient eller om problem uppstår under studien ska du vända dig till:

Ansvarig prövare på stroke-avdelningen: XXXX XXXX

För studien ansvarig läkare:

Johan Engdahl, Överläkare/Docent
Karolinska Institutet, Danderyds Sjukhus
Hjärtkliniken
Danderyds Sjukhus
182 57 Danderyd
Johan.engdahl@sll.se

Patientnummer:________________

Bilaga 05

Randomiserad jämförelse mellan standardiserad och utökad EKG-undersökning efter stroke eller TIA för att detektera förmaksflimmer och minska risken för stroke - AF SPICE

Samtycke till att delta i studien

Jag har fått muntlig och skriftlig informationen om studien och har haft möjlighet att ställa frågor. Jag får behålla den skriftliga informationen.

☐ Jag samtycker till att delta i studien *Randomiserad jämförelse mellan standardiserad och utökad EKG-undersökning efter stroke eller TIA för att detektera förmaksflimmer och minska risken för stroke - AF SPICE*

☐ Jag samtycker till att uppgifter om mig behandlas på det sätt som beskrivs i forskningspersonsinformationen.

☐ Jag samtycker till att uppgifter om mig ur de olika register som beskrivs ovan utförs som långtidsuppföljning

Ort Datum ______________________

____

Forskningspersons namnteckning

____

Namnförtydligande

Jag har förklarat studiens upplägg och syfte, samt inhämtat samtycke för deltagande:

Ort Datum ______________________

____

Namnteckning prövare

____

Namnförtydligande

For peer review only - http://bmjopen.bmj.com/site/about/guidelines.xhtml

References

1. Diener HC, Connolly SJ, Ezekowitz MD, Wallentin L, Reilly PA, Yang S, Xavier D, Di Pasquale G and Yusuf S. Dabigatran compared with warfarin in patients with atrial fibrillation and previous transient ischaemic attack or stroke: a subgroup analysis of the RE-LY trial. *Lancet Neurol*. 2010;9:1157-63.

2. Diener HC, Eikelboom J, Connolly SJ, Joyner CD, Hart RG, Lip GY, O'Donnell M, Hohnloser SH, Hankey GJ, Shestakovska O, Yusuf S, Committee AS and Investigators. Apixaban versus aspirin in patients with atrial fibrillation and previous stroke or transient ischaemic attack: a predefined subgroup analysis from AVERROES, a randomised trial. *Lancet Neurol*. 2012;11:225-31.

3. Amarenco P, Lavallée PC, Labreuche J, Albers GW, Bornstein NM, Canhão P, Caplan LR, Donnan GA, Ferro JM, Hennerici MG, Molina C, Rothwell PM, Sissani L, Školoudík D, Steg PG, Touboul PJ, Uchiyama S, Vicaut É and Wong LK. One-Year Risk of Stroke after Transient Ischemic Attack or Minor Stroke. *N Engl J Med*. 2016;374:1533-42.

4. Andersen SD, Gorst-Rasmussen A, Lip GY, Bach FW and Larsen TB. Recurrent Stroke: The Value of the CHA2DS2VASc Score and the Essen Stroke Risk Score in a Nationwide Stroke Cohort. *Stroke*. 2015;46:2491-7.

5. Wachter R, Groschel K, Gelbrich G, Hamann GF, Kermer P, Liman J, Seegers J, Wasser K, Schulte A, Jurries F, Messerschmid A, Behnke N, Groschel S, Uphaus T, Grings A, Ibis T, Klimpe S, Wagner-Heck M, Arnold M, Protsenko E, Heuschmann PU, Conen D, Weber-Kruger M, Find AFI and Coordinators. Holter-electrocardiogram-monitoring in patients with acute ischaemic stroke (Find-AFRANDOMISED): an open-label randomised controlled trial. *Lancet Neurol*. 2017;16:282-290.

6. Hankey GJ, Patel MR, Stevens SR, Becker RC, Breithardt G, Carolei A, Diener HC, Donnan GA, Halperin JL, Mahaffey KW, Mas JL, Massaro A, Norrving B, Nessel CC, Paolini JF, Roine RO, Singer DE, Wong L, Califf RM, Fox KA, Hacke W and Investigators RASC. Rivaroxaban compared with warfarin in patients with atrial fibrillation and previous stroke or transient ischaemic attack: a subgroup analysis of ROCKET AF. *Lancet Neurol*. 2012;11:315-22.

For peer review only - http://bmjopen.bmj.com/site/about/guidelines.xhtml

7. Easton JD, Lopes RD, Bahit MC, Wojdyla DM, Granger CB, Wallentin L, Alings M, Goto S, Lewis BS, Rosenqvist M, Hanna M, Mohan P, Alexander JH, Diener HC, Committees A and Investigators. Apixaban compared with warfarin in patients with atrial fibrillation and previous stroke or transient ischaemic attack: a subgroup analysis of the ARISTOTLE trial. *Lancet Neurol*. 2012;11:503-11.

8. Rost NS, Giugliano RP, Ruff CT, Murphy SA, Crompton AE, Norden AD, Silverman S, Singhal AB, Nicolau JC, SomaRaju B, Mercuri MF, Antman EM, Braunwald E and Investigators EA-T. Outcomes With Edoxaban Versus Warfarin in Patients With Previous Cerebrovascular Events: Findings From ENGAGE AF-TIMI 48 (Effective Anticoagulation With Factor Xa Next Generation in Atrial Fibrillation-Thrombolysis in Myocardial Infarction 48). *Stroke*. 2016;47:2075-82.

9. Lip GY, Hunter TD, Quiroz ME, Ziegler PD and Turakhia MP. Atrial Fibrillation Diagnosis Timing, Ambulatory ECG Monitoring Utilization, and Risk of Recurrent Stroke. *Circ Cardiovasc Qual Outcomes*. 2017;10.

10. Jabaudon D, Sztajzel J, Sievert K, Landis T and Sztajzel R. Usefulness of ambulatory 7-day ECG monitoring for the detection of atrial fibrillation and flutter after acute stroke and transient ischemic attack. *Stroke*. 2004;35:1647-51.

11. Shafqat S, Kelly PJ and Furie KL. Holter monitoring in the diagnosis of stroke mechanism. *Intern Med J*. 2004;34:305-9.

12. Alhadramy O, Jeerakathil TJ, Majumdar SR, Najjar E, Choy J and Saqqur M. Prevalence and predictors of paroxysmal atrial fibrillation on Holter monitor in patients with stroke or transient ischemic attack. *Stroke*. 2010;41:2596-600.

13. Rizos T, Rasch C, Jenetzky E, Hametner C, Kathoefer S, Reinhardt R, Hepp T, Hacke W and Veltkamp R. Detection of paroxysmal atrial fibrillation in acute stroke patients. *Cerebrovasc Dis*. 2010;30:410-7.

14. Doliwa Sobocinski P, Anggardh Rooth E, Frykman Kull V, von Arbin M, Wallen H and Rosenqvist M. Improved screening for silent atrial fibrillation after ischaemic stroke. *Europace*. 2012;14:1112-6.

For peer review only - http://bmjopen.bmj.com/site/about/guidelines.xhtml

15. Lazzaro MA, Krishnan K and Prabhakaran S. Detection of atrial fibrillation with concurrent holter monitoring and continuous cardiac telemetry following ischemic stroke and transient ischemic attack. *J Stroke Cerebrovasc Dis*. 2012;21:89-93.

16. Gumbinger C, Krumsdorf U, Veltkamp R, Hacke W and Ringleb P. Continuous monitoring versus HOLTER ECG for detection of atrial fibrillation in patients with stroke. *Eur J Neurol*. 2012;19:253-7.

17. Suissa L, Lachaud S and Mahagne MH. Optimal Timing and Duration of Continuous Electrocardiographic Monitoring for Detecting Atrial Fibrillation in Stroke Patients. *J Stroke Cerebrovasc Dis*. 2012.

18. Thakkar S and Bagarhatta R. Detection of paroxysmal atrial fibrillation or flutter in patients with acute ischemic stroke or transient ischemic attack by Holter monitoring. *Indian Heart J*. 2014;66:188-92.

19. Bansil S and Karim H. Detection of atrial fibrillation in patients with acute stroke. *J Stroke Cerebrovasc Dis*. 2004;13:12-5.

20. Barthelemy JC, Feasson-Gerard S, Garnier P, Gaspoz JM, Da Costa A, Michel D and Roche F. Automatic cardiac event recorders reveal paroxysmal atrial fibrillation after unexplained strokes or transient ischemic attacks. *Ann Noninvasive Electrocardiol*. 2003;8:194-9.

21. Wallmann D, Tuller D, Wustmann K, Meier P, Isenegger J, Arnold M, Mattle HP and Delacretaz E. Frequent atrial premature beats predict paroxysmal atrial fibrillation in stroke patients: an opportunity for a new diagnostic strategy. *Stroke*. 2007;38:2292-4.

22. Tayal AH, Tian M, Kelly KM, Jones SC, Wright DG, Singh D, Jarouse J, Brillman J, Murali S and Gupta R. Atrial fibrillation detected by mobile cardiac outpatient telemetry in cryptogenic TIA or stroke. *Neurology*. 2008;71:1696-701.

23. Elijovich L, Josephson SA, Fung GL and Smith WS. Intermittent atrial fibrillation may account for a large proportion of otherwise cryptogenic stroke: a study of 30-day cardiac event monitors. *J Stroke Cerebrovasc Dis*. 2009;18:185-9.

For peer review only - http://bmjopen.bmj.com/site/about/guidelines.xhtml

24. Bhatt A, Majid A, Razak A, Kassab M, Hussain S and Safdar A. Predictors of occult paroxysmal atrial fibrillation in cryptogenic strokes detected by long-term noninvasive cardiac monitoring. *Stroke Res Treat*. 2011;2011:172074.

25. Flint AC, Banki NM, Ren X, Rao VA and Go AS. Detection of paroxysmal atrial fibrillation by 30-day event monitoring in cryptogenic ischemic stroke: the Stroke and Monitoring for PAF in Real Time (SMART) Registry. *Stroke*. 2012;43:2788-90.

26. Higgins P, MacFarlane PW, Dawson J, McInnes GT, Langhorne P and Lees KR. Noninvasive cardiac event monitoring to detect atrial fibrillation after ischemic stroke: a randomized, controlled trial. *Stroke*. 2013;44:2525-31.

27. Miller DJ, Khan MA, Schultz LR, Simpson JR, Katramados AM, Russman AN and Mitsias PD. Outpatient cardiac telemetry detects a high rate of atrial fibrillation in cryptogenic stroke. *J Neurol Sci*. 2013;324:57-61.

28. Gladstone DJ, Spring M, Dorian P, Panzov V, Thorpe KE, Hall J, Vaid H, O'Donnell M, Laupacis A, Cote R, Sharma M, Blakely JA, Shuaib A, Hachinski V, Coutts SB, Sahlas DJ, Teal P, Yip S, Spence JD, Buck B, Verreault S, Casaubon LK, Penn A, Selchen D, Jin A, Howse D, Mehdiratta M, Boyle K, Aviv R, Kapral MK and Mamdani M. Atrial fibrillation in patients with cryptogenic stroke. *N Engl J Med*. 2014;370:2467-77.

29. Sebasigari D, Merkler A, Guo Y, Gialdini G, Kummer B, Hemendinger M, Song C, Chu A, Cutting S, Silver B, Elkind MSV, Kamel H, Furie KL and Yaghi S. Biomarkers of Atrial Cardiopathy and Atrial Fibrillation Detection on Mobile Outpatient Continuous Telemetry After Embolic Stroke of Undetermined Source. *J Stroke Cerebrovasc Dis*. 2017;26:1249-1253.

30. Kaura A, Sztriha L, Chan FK, Aeron-Thomas J, Gall N, Piechowski-Jozwiak B, Teo JT. Early prolonged ambulatory cardiac monitoring in stroke (EPACS): an open-label randomised controlled trial. *Eur J Med Res.* 2019;24(1):25.

For peer review only

For peer review only - http://bmjopen.bmj.com/site/about/guidelines.xhtml

SPIRIT 2013 Checklist: Recommended items to address in a clinical trial protocol and related documents*

Section/item	ItemNo	Description	Comment/finished
**Administrative information**			
Title	1	Descriptive title identifying the study design, population, interventions, and, if applicable, trial acronym	Page 1
Trial registration	2a	Trial identifier and registry name. If not yet registered, name of intended registry	Page 4
	2b	All items from the World Health Organization Trial Registration Data Set	
Protocol version	3	Date and version identifier	Page 4
Funding	4	Sources and types of financial, material, and other support	Page 24
Roles and responsibilities	5a	Names, affiliations, and roles of protocol contributors	Page 24
	5b	Name and contact information for the trial sponsor	Page 4

For peer review only - http://bmjopen.bmj.com/site/about/guidelines.xhtml

	5c	Role of study sponsor and funders, if any, in study design; collection, management, analysis, and interpretation of data; writing of the report; and the decision to submit the report for publication, including whether they will have ultimate authority over any of these activities	
	5d	Composition, roles, and responsibilities of the coordinating centre, steering committee, endpoint adjudication committee, data management team, and other individuals or groups overseeing the trial, if applicable (see Item 21a for data monitoring committee)	
**Introduction**			
Background and rationale	6a	Description of research question and justification for undertaking the trial, including summary of relevant studies (published and unpublished) examining benefits and harms for each intervention	Page 6-8
	6b	Explanation for choice of comparators	Page 8
Objectives	7	Specific objectives or hypotheses	
Trial design	8	Description of trial design including type of trial (eg, parallel group, crossover, factorial, single group), allocation ratio, and framework (eg, superiority, equivalence, noninferiority, exploratory)	Page 9

For peer review only

Methods: Participants, interventions, and outcomes

Study setting	9	Description of study settings (eg, community clinic, academic hospital) and list of countries where data will be collected. Reference to where list of study sites can be obtained	Page 10
Eligibility criteria	10	Inclusion and exclusion criteria for participants. If applicable, eligibility criteria for study centres and individuals who will perform the interventions (eg, surgeons, psychotherapists)	Page 10-11
Interventions	11a	Interventions for each group with sufficient detail to allow replication, including how and when they will be administered	Page 12-15
	11b	Criteria for discontinuing or modifying allocated interventions for a given trial participant (eg, drug dose change in response to harms, participant request, or improving/worsening disease)	Page 11
	11c	Strategies to improve adherence to intervention protocols, and any procedures for monitoring adherence (eg, drug tablet return, laboratory tests)	
	11d	Relevant concomitant care and interventions that are permitted or prohibited during the trial	

For peer review only - http://bmjopen.bmj.com/site/about/guidelines.xhtml

Outcomes	12	Primary, secondary, and other outcomes, including the specific measurement variable (eg, systolic blood pressure), analysis metric (eg, change from baseline, final value, time to event), method of aggregation (eg, median, proportion), and time point for each outcome. Explanation of the clinical relevance of chosen efficacy and harm outcomes is strongly recommended	Page 15
Participant timeline	13	Time schedule of enrolment, interventions (including any run-ins and washouts), assessments, and visits for participants. A schematic diagram is highly recommended (see Figure)	Page 15
Sample size	14	Estimated number of participants needed to achieve study objectives and how it was determined, including clinical and statistical assumptions supporting any sample size calculations	Page 16-17
Recruitment	15	Strategies for achieving adequate participant enrolment to reach target sample size	Page 17

Methods: Assignment of interventions (for controlled trials)

Allocation:

For peer review only - http://bmjopen.bmj.com/site/about/guidelines.xhtml

Sequence generation	16a	Method of generating the allocation sequence (eg, computer-generated random numbers), and list of any factors for stratification. To reduce predictability of a random sequence, details of any planned restriction (eg, blocking) should be provided in a separate document that is unavailable to those who enrol participants or assign interventions	Page 17
Allocation concealment mechanism	16b	Mechanism of implementing the allocation sequence (eg, central telephone; sequentially numbered, opaque, sealed envelopes), describing any steps to conceal the sequence until interventions are assigned	
Implementation	16c	Who will generate the allocation sequence, who will enrol participants, and who will assign participants to interventions	
Blinding (masking)	17a	Who will be blinded after assignment to interventions (eg, trial participants, care providers, outcome assessors, data analysts), and how	Page 18
	17b	If blinded, circumstances under which unblinding is permissible, and procedure for revealing a participant's allocated intervention during the trial	

Methods: Data collection, management, and analysis

Data collection methods	18a	Plans for assessment and collection of outcome, baseline, and other trial data, including any related processes to promote data quality (eg, duplicate measurements, training of assessors) and a description of study instruments (eg, questionnaires, laboratory tests) along with their reliability and validity, if known. Reference to where data collection forms can be found, if not in the protocol	Page 18-19
	18b	Plans to promote participant retention and complete follow-up, including list of any outcome data to be collected for participants who discontinue or deviate from intervention protocols	Page 19
Data management	19	Plans for data entry, coding, security, and storage, including any related processes to promote data quality (eg, double data entry; range checks for data values). Reference to where details of data management procedures can be found, if not in the protocol	
Statistical methods	20a	Statistical methods for analysing primary and secondary outcomes. Reference to where other details of the statistical analysis plan can be found, if not in the protocol	Page 19-20
	20b	Methods for any additional analyses (eg, subgroup and adjusted analyses)	NA

	20c	Definition of analysis population relating to protocol non-adherence (eg, as randomised analysis), and any statistical methods to handle missing data (eg, multiple imputation)	
**Methods: Monitoring**			
Data monitoring	21a	Composition of data monitoring committee (DMC); summary of its role and reporting structure; statement of whether it is independent from the sponsor and competing interests; and reference to where further details about its charter can be found, if not in the protocol. Alternatively, an explanation of why a DMC is not needed	Page 20
	21b	Description of any interim analyses and stopping guidelines, including who will have access to these interim results and make the final decision to terminate the trial	N/A
Harms	22	Plans for collecting, assessing, reporting, and managing solicited and spontaneously reported adverse events and other unintended effects of trial interventions or trial conduct	Page 21
Auditing	23	Frequency and procedures for auditing trial conduct, if any, and whether the process will be independent from investigators and the sponsor	Page 21
**Ethics and dissemination**			

Research ethics approval	24	Plans for seeking research ethics committee/institutional review board (REC/IRB) approval	Page 22
Protocol amendments	25	Plans for communicating important protocol modifications (eg, changes to eligibility criteria, outcomes, analyses) to relevant parties (eg, investigators, REC/IRBs, trial participants, trial registries, journals, regulators)	Page 22
Consent or assent	26a	Who will obtain informed consent or assent from potential trial participants or authorised surrogates, and how (see Item 32)	Page 23
	26b	Additional consent provisions for collection and use of participant data and biological specimens in ancillary studies, if applicable	
Confidentiality	27	How personal information about potential and enrolled participants will be collected, shared, and maintained in order to protect confidentiality before, during, and after the trial	Page 23
Declaration of interests	28	Financial and other competing interests for principal investigators for the overall trial and each study site	Page 24
Access to data	29	Statement of who will have access to the final trial dataset, and disclosure of contractual agreements that limit such access for investigators	Page 25

For peer review only

Ancillary and post-trial care	30	Provisions, if any, for ancillary and post-trial care, and for compensation to those who suffer harm from trial participation	N/A
Dissemination policy	31a	Plans for investigators and sponsor to communicate trial results to participants, healthcare professionals, the public, and other relevant groups (eg, via publication, reporting in results databases, or other data sharing arrangements), including any publication restrictions	
	31b	Authorship eligibility guidelines and any intended use of professional writers	
	31c	Plans, if any, for granting public access to the full protocol, participant-level dataset, and statistical code	
**Appendices**			
Informed consent materials	32	Model consent form and other related documentation given to participants and authorised surrogates	Appendix
Biological specimens	33	Plans for collection, laboratory evaluation, and storage of biological specimens for genetic or molecular analysis in the current trial and for future use in ancillary studies, if applicable	

For peer review only

*It is strongly recommended that this checklist be read in conjunction with the SPIRIT 2013 Explanation & Elaboration for important clarification on the items. Amendments to the protocol should be tracked and dated. The SPIRIT checklist is copyrighted by the SPIRIT Group under the Creative Commons "Attribution-NonCommercial-NoDerivs 3.0 Unported" license.

For peer review only

BMJ Open

A multicenter, national, investigator-initiated, randomised, parallel-group, register based superiority trial to compare extended ECG monitoring versus standard ECG monitoring in elderly patients with ischemic stroke or transient ischemic attack and the effect on stroke, death and intracerebral bleeding – The AF SPICE protocol.

Journal:	*BMJ Open*
Manuscript ID	bmjopen-2023-073470.R2
Article Type:	Protocol
Date Submitted by the Author:	30-Oct-2023
Complete List of Authors:	Engdahl, Johan; Karolinska Institutet, Department of Clinical Sciences, Danderyd Hospital<br>Straat, Kajsa; Karolinska Institutet, Department of clinical sciences, Danderyd Hospital<br>Isaksson, Eva; Karolinska Institutet, Department of clinical sciences, Danderyd Hospital<br>Rooth, Elisabeth; Karolinska Institutet, Department of clinical Sciences, Danderyd Hospital<br>Svennberg, Emma; Karolinska Institutet, Department of Medicine, Huddinge, Karolinska University Hospital,<br>Norrving, Bo; Lund University, Section of Neurology, Department of Clinical Sciences<br>Euler, Mia; Orebro universitet, School of Medicine, Department of Neurology<br>Hellqvist, Kjersti; Alingsas lasarett, Department of Medicine<br>Gu, Weigang; Karolinska Institutet, Department of Clinical Sciences, South Hospital<br>Ström, Jakob; Orebro universitet, School of Medicine, Department of Neurology<br>Själander, Sara; Umeå University, Department of Public Health and Clinical Medicine<br>Eriksson, Marie; Umeå University, Department of Statistics, USBE<br>Åsberg, Signild; Uppsala University, Department of Medical Sciences<br>Wester, Per; Umeå University, Department of Public Health and Clinical Medicine; Karolinska Institutet, Dept of Clinical Sciences, Danderyd Hospital
**Primary Subject Heading**:	Cardiovascular medicine
Secondary Subject Heading:	Neurology
Keywords:	Stroke < NEUROLOGY, Cardiology < INTERNAL MEDICINE, Thromboembolism < CARDIOLOGY

A multicenter, national, investigator-initiated, randomised, parallel-group, register based superiority trial to compare extended ECG monitoring versus standard ECG monitoring in elderly patients with ischemic stroke or transient ischemic attack and the effect on stroke, death and intracerebral bleeding – The AF SPICE protocol.

Johan Engdahl[1], Kajsa Strååt[1], Eva Isaksson[1], Elisabeth Rooth[1], Emma Svennberg[2], Bo Norrving[3], Mia von Euler[4], Kjersti Hellqvist[5], Weigang Gu[6], Jakob O Ström[4], Sara Själander[7], Marie Eriksson[8], Signild Åsberg[9]*, Per Wester[1,7]*

*Shared senior authorship

1. Karolinska Institutet, Dept of Clinical Sciences, Danderyd Hospital, Stockholm, Sweden.

2. Karolinska Institutet, Dept of Medicine, Huddinge, Karolinska University Hospital, Stockholm, Sweden.

3. Section of Neurology, Department of Clinical Sciences, Lund University, Lund, Sweden.

4. School of Medicine, Department of Neurology and Rehabilitation, Örebro University, Örebro, Sweden

5. Department of Medicine, Alingsås Hospital, Alingsås, Sweden.

6. Karolinska Institutet, Dept of Clinical Sciences, South Hospital, Stockholm, Sweden.

7. Department of Public Health and Clinical Medicine, Umeå University, Umeå, Sweden.

8. Dept of Statistics, USBE, Umeå University, Umeå, Sweden

9. Dept of Medical Sciences, Uppsala University, Uppsala, Sweden

Corresponding author:

Johan Engdahl

Department of Cardiology

Danderyd University Hospital

SE-182 88 Stockholm

For peer review only - http://bmjopen.bmj.com/site/about/guidelines.xhtml

Sweden

Email: johan.engdahl@regionstockholm.se

Phone: +46 737 593374

Fax: +46 8 7550868

Word count: main 3903 (excluding CIS); abstract 293

For peer review only

ABSTRACT

Introduction

Atrial fibrillation (AF) is a major risk factor for ischemic stroke and transient ischemic attack (TIA), and AF detection can be challenged by asymptomatic and paroxysmal presentation. Long-term ECG monitoring after ischemic stroke or TIA is recommended by all major societies in cardiology and cerebrovascular medicine as a secondary prophylactic measure. However, data on stroke reduction are lacking, and the recommendations show significant diversity.

Methods and analysis

AF SPICE is a multicenter, national, investigator-initiated, randomised, parallel-group, register-based trial comparing extended ECG monitoring versus standard ECG monitoring in patients admitted with ischemic stroke or TIA, with a composite endpoint of stroke, all-cause-mortality and intracerebral bleeding. Patients aged ≥70 years, without previous AF will be randomised 1:1 to control (standard ECG monitoring) or intervention (extended ECG monitoring). In the control arm, patients will undergo 48h±24h (i.e. range 24-72 hours) of continuous ECG monitoring according to national recommendations. In the intervention arm, patients will undergo 14+14 days of continuous ECG monitoring three months apart using an ECG patch device, which will provide an easy-accessed, well-tolerated 14-days continuous ECG recording. All ECG patch recordings will be read in a core facility. In case of AF detection, oral anticoagulation will be recommended if not contraindicated. A pilot phase has been concluded in 2022 which will transcend into the main trial during 2023-2026,

For peer review only - http://bmjopen.bmj.com/site/about/guidelines.xhtml

including approximately 30 stroke units. The sample size is calculated to 3,262 patients. The primary outcome will be collected from register data during a 36-month follow-up.

Ethics and dissemination

Ethical approval has been provided by the Swedish Ethical Review Authority, reference 2021-02770. The trial will be conducted according to the ethical principles of the declaration of Helsinki and national regulatory standards. Positive results from the study have the potential of rapid dissemination in clinical practice.

Trial Registration

ClinicalTrials.gov NCT05134454

Trial protocol version 2.0

Sponsor contact information:

Karolinska Institutet

Department of clinical sciences

Danderyd Hospital

SE-18288 Danderyd

Sweden

For peer review only

For peer review only - http://bmjopen.bmj.com/site/about/guidelines.xhtml

STRENGTHS AND LIMITATIONS OF THE TRIAL

- First register-based randomised clinical trial on secondary preventive ECG screening post stroke/TIA with stroke and all-cause mortality endpoints
- Participants included from a national network of stroke units and central adjudication of ECG recordings in the intervention arm
- Robust inclusion criteria mirroring clinical real-world population, age at least 70 years for inclusion
- The Swedish stroke register (Riksstroke) used for provision of majority of baseline data
- Endpoints from health care registers will not be further adjudicated

For peer review only

INTRODUCTION

Ischemic stroke is one of the leading causes of mortality worldwide, and a major cause behind permanent disability in adults(1). Despite implementation of novel treatment strategies, morbidity and mortality remain significant.

Modifiable risk factors for stroke include hypertension, atrial fibrillation (AF), hyperlipidaemia, smoking and diabetes(2, 3). Several of these risk factors are being undetected for a long time since they remain asymptomatic.

During 2018, the stroke incidence in Sweden was 223 per 100 000 in men and 191 per 100 000 in women(4). At three-month follow up post-stroke, 26% were deceased or dependent in activities of daily living(5). (4)The risk for stroke recurrence is highest during the first 3-6 months following admission, and the one-year recurrence risk has been reported to be 3-5%(6, 7).

The societal burden of the disease is significant. Disability with need for long-term care, and in younger patients impaired work ability entails enormous societal costs(8). Strokes due to AF are more severe and have a significantly higher mortality than other stroke cases(9).

AF is the most prevalent clinically arrhythmia with a steeply increasing incidence with advancing age(10). AF is also one of the strongest risk factors for stroke(3). However, the increased stroke risk associated with AF can be markedly reduced by oral anticoagulation (OAC) treatment(11). Unfortunately, AF is paroxysmal and asymptomatic in a significant proportion of stroke patients, leading to lower detection rates, and frequently leaves stroke survivors with an undiagnosed and untreated risk factor and higher risk of stroke recurrence(12). Despite this, no study so far has reported evidence to the benefit of

For peer review only

For peer review only - http://bmjopen.bmj.com/site/about/guidelines.xhtml

electrocardiographic (ECG) AF screening in terms of reduced stroke recurrence and mortality after a stroke event.

(13)Randomised controlled trials (CRYSTAL-AF(14) and EMBRACE(12)) have shown that prolonged ECG-monitoring compared to standard of care (at the time 24 hr-ECG) increased detection of atrial fibrillation in cryptogenic stroke patients.

The FIND-AF$_{randomised}$ trial(15) also showed increased detection of atrial fibrillation and included a broader group of participants suggesting that prolonged ECG-monitoring should be used in all ischemic stroke patients.

The MonDAFIS study(16) was powered to evaluate the proportion of anticoagulation therapy 12 months after index stroke. Standard of care was compared to prolonged in hospital ECG-monitoring (maximum 7 days). Atrial fibrillation detection was increased in the intervention group, but this had no significant effect on the rate of anticoagulation therapy at 12 months.

AF screening post an ischemic stroke event is currently recommended by major international societies (Table 1)(17-23). The recommendations vary markedly with respect to method of screening and duration of screening. There are however a body of data on the proportion of AF detection using different monitoring strategies(24).

The development of ECG modalities and clinical practice for AF screening post ischemic stroke and TIA have evolved towards longer and more resource-demanding investigations as noted in table 1, despite the lack of data on clinical endpoints like stroke. Diversity within recommendations have promoted unequal care even within countries and regions. With this background, there is an urgent need for a randomised trial to investigate the efficacy of secondary prophylactic AF screening following ischemic stroke.

For peer review only

For peer review only - http://bmjopen.bmj.com/site/about/guidelines.xhtml

Choice of comparators

In some of the recommendations on AF-screening post-ischemic stroke from international societies, a basic monitoring duration of 24 hours of continuous ECG is recommended(18, 20). In the recommendations from the Swedish Board of Health and Welfare, continuous ECG monitoring of 24-48 hours is recommended as the baseline investigation for ischemic stroke patients, and a large share of stroke units in Sweden adhere to this recommendation.

METHODS AND ANALYSIS

Primary Objective

To determine if extended continuous ECG screening (14+14 days) is superior to standard ECG screening (48 ±24 hours i.e. range 24-72 hours) in reducing the risk of the combined endpoint of ischemic stroke, intracerebral bleeding and all-cause mortality in elderly patients admitted to hospital for ischemic stroke or TIA.

Key Secondary Objectives

The key secondary objectives are to estimate the treatment effect in primary outcome, and determine if there are any differences between extended and standard ECG screening for:

- Ischemic stroke
- All-cause mortality
- Intracranial bleeding
- Major bleeding

For peer review only - http://bmjopen.bmj.com/site/about/guidelines.xhtml

For peer review only

- Myocardial infarction
- Pacemaker implantation
- Cost effectiveness

Other Secondary Objectives

- To study association between age and AF detection
- To study the proportion of other relevant arrhythmias detected at ECG screening
- To study predictors for AF detection
- To study signal time and signal quality achieved at ECG screening
- To study long-term adherence to OAC treatment
- To study feasibility of patients application of the second ECG patch
- To study Patient Reported Experience (PREM), collected 3 months after inclusion in the main trial
- Per protocol analysis of the primary outcome
- Win ratio analysis

Trial design

AF SPICE is designed as a multicenter, national, investigator-initiated, randomised, parallel-group, register based superiority trial to compare extended ECG monitoring versus standard ECG Monitoring in Elderly Patients admitted with ischemic stroke or TIA with the combined endpoint of recurrent ischemic stroke, all-cause mortality and intracerebral bleeding (Figure 1).

For peer review only

For peer review only - http://bmjopen.bmj.com/site/about/guidelines.xhtml

Study setting

AF SPICE will recruit participants among admissions for ischemic stroke or TIA in stroke units in Swedish hospitals. Inclusion is also possible from non-stroke units given that the patient is admitted for ischemic stroke or TIA and identified by the site's local investigator or delegate.

A pilot trial commenced in January 2022 and is now concluded, converting into the main study in 2023. The pilot trial evaluated inclusion rates, feasibility and adherence to ECG recorders and the database with randomisation system.

Eligibility criteria

Inclusion criteria

1. Patients aged ≥ 70 years admitted to a stroke unit with a diagnosis of ischemic stroke or TIA within 14 days from inclusion. Stroke is clinically defined as an acute onset of focal neurological deficit of presumed vascular origin lasting for ≥ 24 hours or resulting in death. Stroke is further categorized as ischemic or as intracerebral or subarachnoid haemorrhage based on computed tomography (CT) and/or magnetic resonance imaging (MRI) of the brain. Transient ischemic attack (TIA) is defined as a sudden, focal neurological deficit of presumed vascular origin with transient symptoms (< 24 hours)(25). Only patients with ischemic stroke and TIA are eligible for inclusion.
2. Signed informed consent.

For peer review only - http://bmjopen.bmj.com/site/about/guidelines.xhtml

Exclusion criteria

1. Previously diagnosed AF
2. Contraindication to oral anticoagulant treatment
3. Indication for anticoagulant treatment other than AF.
4. Patients with pacemaker, implantable cardioverter defibrillator or implantable loop recorder.
5. Patients who, according to the investigator, will not be able to comply with the trial protocol.
6. Previous participation in the AF SPICE trial

The rationale for the age cut-off of 70 years in AF SPICE is partly due to the higher AF yield in elderly patients, as well as the increased benefit for oral anticoagulation treatment in elderly patient in the case of AF detection. Notably, 74% of stroke patients in Sweden are aged 70 years of above, making the study population quite representative of the future implementation. The recruitment window of 14 days is intended to give a margin for the occurrence of patient transfer between centres for thrombectomy, holidays and uncertainty about eligibility. Patients with a cardiac implantable device are excluded since their very long-lasting continuous heart rhythm monitoring will make them biased with a lower probability of AF detection on a surface ECG recording. Further, ventricular pacing will make reading of the patch ECG difficult due to the lack of pacing annotations.

Withdrawal of patients

A patient can withdraw from the trial at any time, at the discretion of the patient, or by the investigator if deemed medically indicated. If a participant refrain from completing extended

For peer review only

For peer review only - http://bmjopen.bmj.com/site/about/guidelines.xhtml

ECG recording protocol, he/she will remain included in the trial and in the intention-to-screen data analysis. A withdrawal of consent means that no further trial data will be collected, however already collected data will not be erased.

Patient and public involvement

Patient or public involvement is planned but not yet in place in this trial.

Interventions

Extended ECG Investigation

Participants randomised to extended ECG investigation will first undergo the standard 0-2 days of ECG monitoring, before receiving a continuous one-lead ECG-recording using the BioTel ePatch (https://www.biotel.se). The BioTel ePatch is a class IIa CE-marked Holter ECG recorder using a patch for skin adhesion.

The ECG patch will be applied by health care professionals at the stroke unit (visit 1) at randomisation.

After completion of the 14-day ECG recording, the device should be returned using a pre-paid envelope. Following arrival at BioTel, the ECG data will be uploaded to a web-accessed interpretation service (Cardiologs, https://cardiologs.com/) within 1 working day from arrival.

During visit 2, participants not diagnosed with AF will have the second ePatch attached by health care professionals in connection to a scheduled follow-up visit or attached by

For peer review only - http://bmjopen.bmj.com/site/about/guidelines.xhtml

the participant/next of kin. The rationale for the second ePatch recording is to increase AF yield, even though the second recording will reasonably identify fewer cases of AF.

The ECG recordings will be scrutinised by a core ECG reading team led by JE. All events in the ECG readings will be manually overread.

Procedures for handling of arrhythmias detected on extended ECG recording

Atrial fibrillation is defined according to the current definition of the ESC AF guidelines(20), i.e. at least 30 seconds with irregularly irregular R-R intervals, absence of distinct repeating p-waves and irregular atrial activations. When this is met, the local investigator will be notified within one working day by phone and email by the ECG reading team.

If a participant is diagnosed with other arrhythmias with a prognostic implication as defined in table 2, the local investigator will be notified by email within three working days. However, due to the high prevalence of ventricular arrhythmias during 14 days of continuous ECG in this population, a selection based on team discussion is made for VTs. If a more rapid action is deemed necessary by the ECG reading team, appropriate measures will be undertaken. When no significant arrhythmias are noted, the local investigator will be notified within one week from ECG upload.

Procedures for poor signal quality and short recordings in extended ECG

If the signal quality of the extended ECG recording is deemed to be insufficient, the participant should be offered another patch ECG recording. If this second recording has

For peer review only - http://bmjopen.bmj.com/site/about/guidelines.xhtml

insufficient signal quality or if it is not possible to arrange for another patch ECG recording, a traditional Holter recording, or ECG event recording could be used. However, it is of importance for the power of the trial to achieve as long ECG recordings as possible in the intervention group.

At least 7 days of ECG recording should be yielded from ECG patch in visit one. If less than 7 days are recorded, another recording should be offered. The same minimum ECG duration of 7 days applies for ECG recording at visit two.

If a participant randomised to intervention arm with a negative ECG recording (i.e., no atrial fibrillation detected) suffers a recurrent ischemic stroke within 2 months from visit one, the second ECG patch recording (visit 2) may be started earlier than 2 months to the discretion of the local investigator.

Oral anticoagulation initiation following AF detection in extended ECG recording

Patients diagnosed with AF during the long-term ECG recordings initiated in connection to randomisation should be informed by the local investigator and offered treatment with oral anticoagulation if there are no contraindications, based on the stroke risk of at least CHA_2DS_2-VASc 3 points in randomised patients.

Treatment with OAC and its long-term follow-up will be handled within standard care and according to national guidelines.

When indicated, OAC treatment should be initiated within five working days from the reception of long-term ECG results. Long-term follow-up of OAC treatment will be using the national drug prescription registry.

For peer review only - http://bmjopen.bmj.com/site/about/guidelines.xhtml

All other treatments and investigations will be at the discretion of the local investigator.

Standard care

Participants randomised to standard care should undergo 48 ±24 hours of continuous ECG monitoring, using inpatient ECG telemetry monitoring or Holter recording. The duration of the monitoring should be recorded by the site and noted in the electronic case report form (REDCap, Vanderbilt University, TN, United States).

The trial will not collect any data from the standard recordings apart from its duration, and containment of AF. ECG recordings of newly diagnosed AF in the standard care arm will be collected and stored in the trial.

Outcomes

Primary endpoint

Difference between the two arms in a combined endpoint of recurrent ischemic stroke, intracerebral bleeding, and all-cause mortality during at least 36 months of follow-up.

Secondary endpoints

- Individual components of the primary endpoint
- Major bleeding including Intracranial bleeding
- Myocardial infarction
- Pacemaker implantation
- Anticoagulation treatment

For peer review only

- Atrial fibrillation prevalence
- Adherence to long-term ECG recording
- Incidence of other prognostic arrhythmia during long-term ECG recording

Participant timeline intervention arm

Participants will be screened for consent during admission for ischemic stroke or TIA, within 14 days from admission. ECG investigation of 0-48 hours prior to randomisation is allowed. Extended ECG recording is initiated during admission for ischemic stroke/TIA and the participant will return the device via mail. The second extended ECG recording should be initiated at follow-up visit or by mailing the device to the participants for self-application. Following completion of second 14-day ECG recording, no further personal visits are made in the trial.

Participant timeline standard arm

Starting or completing the ECG recording in the standard arm following discharge is allowed. Following completion of the ECG investigation initiated in the standard arm, no further personal visits are made in the trial.

Sample size

The sample size was calculated based on the primary outcome.

There were very few studies available with outcome data matching the age and morbidity of the AF SPICE trial. Very few studies on patients with an index stroke incident were available,

For peer review only

For peer review only - http://bmjopen.bmj.com/site/about/guidelines.xhtml

in particular were data scarce regarding patients with recent stroke and AF without OAC treatment. For this, we have made estimations and extrapolations using the very few available studies on AF patients without OAC treatment. In particular, extrapolations have been made with regard to age since most available studies have included patients considerably younger than what is expected in AF SPICE. A list of studies used in sample size estimations is available in the appendix.

For the estimations of AF detection proportions, available studies on short-term (48 ±24 hours) ECG recording following stroke and corresponding studies on long-term (14 days) recordings were used. As with the outcome data, no studies with similar population and ECG recording duration were available. Further, the AF detection/incidence was estimated for the entire follow-up for both arms, reflecting the additional detection within standard care after the index event, thereby reflecting the actual risk exposure in both arms during follow-up. A list of the AF detection studies available for this estimation is reported in the appendix.

The assumptions for the sample size calculation are reported in table 3. The complete sample size calculation is provided in the appendix. For the yearly risk of the composite endpoint, we estimate 17% in patients with AF and no OAC treatment, 7,3% in patients with AF and OAC treatment and 5% risk in patients without AF. We further estimate an AF detection proportion of 6% in the control group and 19% after two ECG recordings in the intervention group, and 13% with untreated AF in the control group, resulting in a sample size of 2718 for three years of fixed follow-up time after inclusion. Adding 20% for participant dropouts and treatment discontinuation, a total sample size of 3262 is needed, corresponding to 1631 in each arm based on three years follow-up, 80% power and double-

For peer review only - http://bmjopen.bmj.com/site/about/guidelines.xhtml

sided significance level of 5%. The study is powered to detect a 9% reduction of the composite endpoint in the group with extended ECG screening.

Recruitment

Patients will be screened and included during ischemic stroke or TIA admissions in stroke units. Participating centres report at least 200 stroke/TIA admissions yearly. With an anticipated participation of 30 sites, they need to randomise 0.6 patients weekly during three years of inclusion to fulfil the recruitment target of 3262 participants.

Allocation

Participants will be randomly assigned to either control or intervention group with a 1:1 allocation as per a computer-generated randomisation schedule stratified by site in the online REDCap database. Randomisation will be performed immediately following the collection of necessary baseline data.

Blinding

Due to the nature of the intervention, neither participants nor staff will be blinded to allocation.

Data collection methods primary Outcome (Table 4a)

Primary endpoint variables are collected from Swedish health care registers as specified in Table 4a.

For peer review only

For peer review only - http://bmjopen.bmj.com/site/about/guidelines.xhtml

Data collection methods Secondary and exploratory outcomes (Table 4b)

Variables collected for secondary and exploratory outcomes are reported in table 4b.

Baseline data entered at inclusion all participants (supplementary table)

Data entered at baseline in all participants are listed in supplementary table. The remaining baseline variables will be collected from the Riksstroke register[5].

Baseline data entered in control and intervention arms (Appendix)

Data entered at baseline in standard and interventions arms are listed in the Appendix.

Baseline register data from Riksstroke register (Appendix)

Baseline data from Riksstroke are listed in the appendix.

ECG data (Appendix)

There are different ECG variables for the control and intervention arms.

1. In the control arm, the local investigator or delegate will report if ECG was recorded using ECG telemetry and/or Holter recording, the duration of each recording, and whether AF was diagnosed.

2. In the intervention arm, ECG data from patch recordings will be stored in the patch hardware and returned by the participant by mail. Data from ECG patch hardware will be uploaded by the patch provider into the Cardiologs database. ECG patch data will be scrutinized by the core reading team which also enter ECG data manually into the REDCap database.

For peer review only

For the second ECG patch recording initiated 2-4 months following randomisation, data will be collected on whether the patch is applied within health care or by the participant.

Cost Effectiveness

The cost effectiveness analysis will be based on a Markov cohort model where prevalence of AF, use of oral anticoagulants, clinical events and all-cause mortality will be collected from the AF SPICE study. The cost for clinical events, age-specific utilities and stroke death will be collected from the literature. Number of gained life-years, quality-adjusted life years and cost for the screening process will be calculated.

Retention

At the majority of including sites, the second visit for the intervention group coincides with an outpatient follow-up visit after stroke, which will give the participant motivation for attending the visit. Patients receive no reimbursement for participation.

Statistical Methods

The main analysis of outcome will be based on time to event (endpoint) and performed according to the intention to treat principle. The cumulative incidence of the combined endpoint and its individual components will be presented for each treatment group (standard and extended ECG).

For peer review only

392 For the primary analysis, of the combined endpoint, the Log-rank test will be used to test for treatment group differences. Cox proportional hazard regression will be used to estimate the hazard ratios with 95% confidence intervals, and to analyse associations with atrial fibrillation incidence and/or effect modification by age, comorbidity, biometrics, and supraventricular activity.

397

398 The secondary analysis, of each individual components of the primary endpoint, will account for competing risks. We will use cause-specific models (where the rate of each individual endpoint is based on patients who have not yet experienced any of the endpoints) and subdistributional hazard models (where the rate is based on patients who have not yet experience the endpoint under study). Other analysis, including baseline characteristics, will be presented by proportions (binary variables) and means (continuous variables) with 95% confidence intervals in each ECG group. Corresponding group comparisons will be carried out by $\chi 2$-tests and t-tests. An outcome with a p-value < 0.05 will be considered statistically significant.

407

408 Data Monitoring Committee

409 An independent Data Safety and Monitoring Board (DSMB) will be appointed at the start of the main trial, i.e., after the pilot phase. The DSMB will have a consulting role to the steering committee. The DSMB will monitor and annually provide statements on:

For peer review only - http://bmjopen.bmj.com/site/about/guidelines.xhtml

1. Cumulative inclusion rate, balance in baseline characteristics, duration and yield of ECG recordings during the inclusion phase.

2. Event rate in the standard and intervention group annually.

Harms

All diagnostic tools used in the trial are approved by regulatory bodies. Any adverse events noted in connection to investigations or treatment should be reported to authorities according to practice.

For peer review only

Auditing

A monitoring plan for the trial has been established, with the following areas:

1. Investigator qualifications, delegation log

2. Written consent forms, accuracy, and completeness.

3. Subject eligibility

4. Source data, accuracy and completeness 5. CRF entries in REDCap, unverified and incomplete entries by site

6. Independent reader will scrutinize 5-10% of ECG reports in intervention arm.

For peer review only - http://bmjopen.bmj.com/site/about/guidelines.xhtml

ETHICS AND DISSEMINATION

Independent ethics committee

Ethical approval has been provided by the Swedish Ethical Review Authority (Reference 2021-02770). Before entering the trial, verbal and written information is given to the patient and informed consent form is signed. The trial will be conducted according to the ethical principles of the declaration of Helsinki(26) and national regulatory standards.

Ethical considerations

The optimal duration of the ECG investigation following ischemic stroke/TIA is not known.(20)

The ethical issues in this trial are partly connected to the knowledge gap of ECG monitoring duration and the clinical practice that has evolved. Several stroke units are applying ECG monitoring protocols with longer duration than the recommended standard. Randomising patients to a monitoring protocol with shorter ECG screening duration be regarded as lowering investigation ambitions. Since the long-term efficacy of different ECG monitoring strategies is unknown, the randomisation to one of the arms in this trial is considered ethically balanced against the option of continuing using monitoring strategies with unknown efficacy and unknown cost effectiveness.

For peer review only

Protocol amendments

Important modifications to the protocol will be summarized consecutively in the protocol and disseminated to investigators. Major protocol changes, i.e. changes in eligibility criteria or patient information texts are subjected to ethical review.

Consent

Trained research nurses or investigators will introduce the trial. Patients will receive written information. Research nurses or investigators will discuss the trial in light of the written information given, and patients will have an opportunity to discuss the information and ask questions.

In patients with aphasia or inability to write following stroke, it seems reasonable to collect informed consent from next of kin, i.e. a spouse or other close relative if some other mode of communication is established between the patient and the next of kin.

Confidentiality

At the trial sites, all log lists containing personal identification data will be stored securely. All trial data will be stored in a REDCap database, accessible by multifactor authentication. All actions in the REDCap database are logged. Data in the ECG reading system Cardiologs are pseudonymized and accessible with password.

For peer review only

AUTHORS´ CONTRIBUTIONS

JE conceived of the trial and drafted the protocol. PW, KS, SÅ, ME and EI made major contributions to trial design. ER, ES, BN, MvE, KH, WG, JOS and SS made contributions to trial design. All authors approved of the final manuscript. AF SPICE is conducted in collaboration with the Swedish Stroke Register, Riksstroke.

FUNDING STATEMENT

AF SPICE is funded by grants from the Swedish Research Council, the Swedish Heart & Lung Foundation, the Swedish Stroke Foundation, and the Stockholm Region (ALF). The funding sources had no role in the design of this trial and will not have any role during its execution, analyses, interpretation of the data, or decision to submit results.

ACKNOWLEDGEMENT

We are grateful to Ida Hed Myrberg and Letizia Orsini at Karolinska Institutet Biostatistics Core Facility for assistance with the power calculation.

COMPETEING INTEREST STATEMENT

JE has received consultant or lecture fees from Roche Diagnostics, Pfizer, Bristol Myers Squibb, Boehringer Ingelheim, Piotrode and Philips. Research grants from the Swedish Research Council, The Swedish Heart&Lung Foundation, The Swedish Innovation Agency and

For peer review only

For peer review only - http://bmjopen.bmj.com/site/about/guidelines.xhtml

The Stockholm Region. KS, EI report no disclosures. ES reports consultant or lecture fees from Bayer, Bristol-Myers Squibb, Pfizer, Boehringer-Ingelheim, Johnson & Johnson, Merck Sharp & Dome. Research grants from the Swedish Research Council, The Swedish Heart&Lung Foundation, Åke Wibergs Foundation and CIMED. BN reports Data Safety Monitoring Board fees from Astra Zeneca. MvE is the chairman of Riksstroke, the Swedish Stroke Register. JOS reports no disclosures. SÅ reports lecture fees paid to the institution from Bristol-Myers Squibb, Pfizer, Boehringer-Ingelheim, and is a member of the steering committee of Riksstroke, the Swedish Stroke Register. PW reports consulting fees, fee as a Clinical Events Committee member and unrestricted grants from Abbott. PW is a member of the steering committee of Riksstroke, the Swedish Stroke Register.

Access to data

The investigators will have access to the final dataset without any restricions.

For peer review only

For peer review only - http://bmjopen.bmj.com/site/about/guidelines.xhtml

REFERENCES

1. Global, regional, and national burden of stroke and its risk factors, 1990-2019: a systematic analysis for the Global Burden of Disease Study 2019. Lancet Neurol. 2021;20(10):795-820.

2. Hankey GJ. Stroke. Lancet. 2017;389(10069):641-54.

3. O'Donnell MJ, Chin SL, Rangarajan S, Xavier D, Liu L, Zhang H, Rao-Melacini P, Zhang X, Pais P, Agapay S, Lopez-Jaramillo P, Damasceno A, Langhorne P, McQueen MJ, Rosengren A, Dehghan M, Hankey GJ, Dans AL, Elsayed A, Avezum A, Mondo C, Diener HC, Ryglewicz D, Czlonkowska A, Pogosova N, Weimar C, Iqbal R, Diaz R, Yusoff K, Yusufali A, Oguz A, Wang X, Penaherrera E, Lanas F, Ogah OS, Ogunniyi A, Iversen HK, Malaga G, Rumboldt Z, Oveisgharan S, Al Hussain F, Magazi D, Nilanont Y, Ferguson J, Pare G, Yusuf S. Global and regional effects of potentially modifiable risk factors associated with acute stroke in 32 countries (INTERSTROKE): a case-control study. Lancet. 2016;388(10046):761-75.

4. Eriksson M, Åsberg S, Sunnerhagen KS, von Euler M. Sex Differences in Stroke Care and Outcome 2005-2018: Observations From the Swedish Stroke Register. Stroke. 2021:Strokeaha120033893.

For peer review only

For peer review only - http://bmjopen.bmj.com/site/about/guidelines.xhtml

5. Riksstroke. Riksstroke english summary 2021 2023 [Available from: https://www.riksstroke.org/annual-reports-and-graphs/.

6. Andersen SD, Gorst-Rasmussen A, Lip GY, Bach FW, Larsen TB. Recurrent Stroke: The Value of the CHA2DS2VASc Score and the Essen Stroke Risk Score in a Nationwide Stroke Cohort. Stroke. 2015;46(9):2491-7.

7. Khanevski AN, Bjerkreim AT, Novotny V, Naess H, Thomassen L, Logallo N, Kvistad CE. Recurrent ischemic stroke: Incidence, predictors, and impact on mortality. Acta Neurol Scand. 2019;140(1):3-8.

8. Mukundan G, Seidenwurm DJ. Economic and Societal Aspects of Stroke Management. Neuroimaging Clin N Am. 2018;28(4):683-9.

9. Henninger N, Goddeau RP, Jr., Karmarkar A, Helenius J, McManus DD. Atrial Fibrillation Is Associated With a Worse 90-Day Outcome Than Other Cardioembolic Stroke Subtypes. Stroke. 2016;47(6):1486-92.

10. Bjorck S, Palaszewski B, Friberg L, Bergfeldt L. Atrial fibrillation, stroke risk, and warfarin therapy revisited: a population-based study. Stroke. 2013;44(11):3103-8.

For peer review only

For peer review only - http://bmjopen.bmj.com/site/about/guidelines.xhtml

11. Hart RG, Pearce LA, Aguilar MI. Meta-analysis: antithrombotic therapy to prevent stroke in patients who have nonvalvular atrial fibrillation. Ann Intern Med. 2007;146(12):857-67.

12. Gladstone DJ, Spring M, Dorian P, Panzov V, Thorpe KE, Hall J, Vaid H, O'Donnell M, Laupacis A, Cote R, Sharma M, Blakely JA, Shuaib A, Hachinski V, Coutts SB, Sahlas DJ, Teal P, Yip S, Spence JD, Buck B, Verreault S, Casaubon LK, Penn A, Selchen D, Jin A, Howse D, Mehdiratta M, Boyle K, Aviv R, Kapral MK, Mamdani M. Atrial fibrillation in patients with cryptogenic stroke. N Engl J Med. 2014;370(26):2467-77.

13. Grond M, Jauss M, Hamann G, Stark E, Veltkamp R, Nabavi D, Horn M, Weimar C, Kohrmann M, Wachter R, Rosin L, Kirchhof P. Improved Detection of Silent Atrial Fibrillation Using 72-Hour Holter ECG in Patients With Ischemic Stroke: A Prospective Multicenter Cohort Study. Stroke. 2013;44(12):3357-64.

14. Sanna T, Diener HC, Passman RS, Di Lazzaro V, Bernstein RA, Morillo CA, Rymer MM, Thijs V, Rogers T, Beckers F, Lindborg K, Brachmann J. Cryptogenic stroke and underlying atrial fibrillation. N Engl J Med. 2014;370(26):2478-86.

15. Wachter R, Groschel K, Gelbrich G, Hamann GF, Kermer P, Liman J, Seegers J, Wasser K, Schulte A, Jurries F, Messerschmid A, Behnke N, Groschel S, Uphaus T, Grings A, Ibis T, Klimpe S, Wagner-Heck M, Arnold M, Protsenko E, Heuschmann PU, Conen D, Weber-Kruger M, Find AFI, Coordinators. Holter-electrocardiogram-monitoring in patients with acute

For peer review only

For peer review only - http://bmjopen.bmj.com/site/about/guidelines.xhtml

ischaemic stroke (Find-AFRANDOMISED): an open-label randomised controlled trial. Lancet Neurol. 2017;16(4):282-90.

16. Haeusler KG, Kirchhof P, Kunze C, Tütüncü S, Fiessler C, Malsch C, Olma MC, Jawad-Ul-Qamar M, Krämer M, Wachter R, Michalski D, Kraft A, Rizos T, Gröschel K, Thomalla G, Nabavi DG, Röther J, Laufs U, Veltkamp R, Heuschmann PU, Endres M. Systematic monitoring for detection of atrial fibrillation in patients with acute ischaemic stroke (MonDAFIS): a randomised, open-label, multicentre study. Lancet Neurol. 2021;20(6):426-36.

17. NICE AF post stroke Guidelines 2023 [Available from: https://www.nice.org.uk/guidance/dg41/.

18. Andrade JG, Aguilar M, Atzema C, Bell A, Cairns JA, Cheung CC, Cox JL, Dorian P, Gladstone DJ, Healey JS, Khairy P, Leblanc K, McMurtry MS, Mitchell LB, Nair GM, Nattel S, Parkash R, Pilote L, Sandhu RK, Sarrazin JF, Sharma M, Skanes AC, Talajic M, Tsang TSM, Verma A, Verma S, Whitlock R, Wyse DG, Macle L. The 2020 Canadian Cardiovascular Society/Canadian Heart Rhythm Society Comprehensive Guidelines for the Management of Atrial Fibrillation. Can J Cardiol. 2020;36(12):1847-948.

19. Brieger D, Amerena J, Attia J, Bajorek B, Chan KH, Connell C, Freedman B, Ferguson C, Hall T, Haqqani H, Hendriks J, Hespe C, Hung J, Kalman JM, Sanders P, Worthington J, Yan TD, Zwar N. National Heart Foundation of Australia and the Cardiac Society of Australia and New

For peer review only

For peer review only - http://bmjopen.bmj.com/site/about/guidelines.xhtml

Zealand: Australian Clinical Guidelines for the Diagnosis and Management of Atrial Fibrillation 2018. Heart, lung & circulation. 2018;27(10):1209-66.

20. Hindricks G, Potpara T, Dagres N, Arbelo E, Bax JJ, Blomström-Lundqvist C, Boriani G, Castella M, Dan GA, Dilaveris PE, Fauchier L, Filippatos G, Kalman JM, La Meir M, Lane DA, Lebeau JP, Lettino M, Lip GYH, Pinto FJ, Thomas GN, Valgimigli M, Van Gelder IC, Van Putte BP, Watkins CL. 2020 ESC Guidelines for the diagnosis and management of atrial fibrillation developed in collaboration with the European Association for Cardio-Thoracic Surgery (EACTS): The Task Force for the diagnosis and management of atrial fibrillation of the European Society of Cardiology (ESC) Developed with the special contribution of the European Heart Rhythm Association (EHRA) of the ESC. Eur Heart J. 2021;42(5):373-498.

21. Kleindorfer DO, Towfighi A, Chaturvedi S, Cockroft KM, Gutierrez J, Lombardi-Hill D, Kamel H, Kernan WN, Kittner SJ, Leira EC, Lennon O, Meschia JF, Nguyen TN, Pollak PM, Santangeli P, Sharrief AZ, Smith SC, Jr., Turan TN, Williams LS. 2021 Guideline for the Prevention of Stroke in Patients With Stroke and Transient Ischemic Attack: A Guideline From the American Heart Association/American Stroke Association. Stroke. 2021;52(7):e364-e467.

22. Physicians Rco. National guideline for stroke London2016 [Fifth edition:[Available from: https://www.strokeaudit.org/Guideline/Full-Guideline.aspx.

For peer review only - http://bmjopen.bmj.com/site/about/guidelines.xhtml

23. Rubiera M, Aires A, Antonenko K, Lémeret S, Nolte CH, Putaala J, Schnabel RB, Tuladhar AM, Werring DJ, Zeraatkar D, Paciaroni M. European Stroke Organisation (ESO) guideline on screening for subclinical atrial fibrillation after stroke or transient ischaemic attack of undetermined origin. Eur Stroke J. 2022;7(3):Vi.

24. Schnabel RB, Haeusler KG, Healey JS, Freedman B, Boriani G, Brachmann J, Brandes A, Bustamante A, Casadei B, Crijns H, Doehner W, Engstrom G, Fauchier L, Friberg L, Gladstone DJ, Glotzer TV, Goto S, Hankey GJ, Harbison JA, Hobbs FDR, Johnson LSB, Kamel H, Kirchhof P, Korompoki E, Krieger DW, Lip GYH, Lochen ML, Mairesse GH, Montaner J, Neubeck L, Ntaios G, Piccini JP, Potpara TS, Quinn TJ, Reiffel JA, Ribeiro ALP, Rienstra M, Rosenqvist M, Sakis T, Sinner MF, Svendsen JH, Van Gelder IC, Wachter R, Wijeratne T, Yan B. Searching for Atrial Fibrillation Poststroke: A White Paper of the AF-SCREEN International Collaboration. Circulation. 2019;140(22):1834-50.

25. Aho K, Harmsen P, Hatano S, Marquardsen J, Smirnov VE, Strasser T. Cerebrovascular disease in the community: results of a WHO collaborative study. Bull World Health Organ. 1980;58(1):113-30.

26. World Medical Association Declaration of Helsinki: ethical principles for medical research involving human subjects. Jama. 2013;310(20):2191-4.

For peer review only

Table 1 ECG screening post stroke society recommendations

Society	Recommendation Summary	Year	Ref
European Stroke Organisation	In adult patients with ischaemic stroke or TIA of undetermined origin, we recommend a prolonged cardiac monitoring instead of standard 24 h monitoring to increase the detection of subclinical AF.<br><br>In adult patients with ischaemic stroke or TIA of **undetermined origin**, we recommend longer duration of cardiac rhythm monitoring of more than 48 h and if feasible with ILR to increase the detection of subclinical AF.	2022	21
American Heart Association/American Stroke Association	In patients suspected of having a stroke or TIA, an ECG is recommended to screen for atrial fibrillation (AF) and atrial flutter and to assess for other concomitant cardiac conditions.<br><br>In patients with **cryptogenic stroke** who do not have a contraindication to anticoagulation, long-term rhythm monitoring with mobile cardiac outpatient telemetry, implantable loop recorder, or other approach is reasonable to detect intermittent AF.	2021	19
European Society of Cardiology	In patients with acute ischaemic stroke or TIA and without previously known AF, monitoring for AF is recommended using a short-term ECG recording for at least the first 24 h, followed by continuous ECG monitoring for at least 72 h whenever possible<br>In selected stroke patients without previous known AF, additional monitoring using long-term non-invasive ECG monitors or insertable cardiac monitors should be considered, to detect AF.	2020	18
Swedish board of health and welfare	Health care should provide long-term ECG for 24-48 h.<br><br>long-term ECG for more than 48 h duration using Holter recording or inpatient telemetry ECG in selected cases.	2020	
NICE	Reveal LINQ is recommended as an option to help to detect atrial fibrillation after **cryptogenic stroke** only if:<br><br>non-invasive electrocardiogram (ECG) monitoring has been done **and** a cardiac arrhythmic cause of stroke is still suspected.	2020	15
Canadian Cardiovascular Society/Canadian Heart Rhythm Society	We recommend at least 24 hours of ambulatory ECG monitoring to identify AF in patients with **nonlacunar ESUS**We suggest additional monitoring for AF detection (eg, prolonged external loop recorder or implantable cardiac monitoring, where available) be performed for selected older patients with **nonlacunar ESUS** in whom AF is suspected but unproven	2020	16
National Heart Foundation of Australia and the Cardiac Society of Australia and New Zealand	Screening for Atrial Fibrillation in Patients with **Embolic Stroke of Uncertain Source** Recommendation: For patients with **ESUS**, longer term ECG monitoring (external or implantable) should be used.	2018	17
Royal College of Physicians	People with ischaemic stroke or TIA who would be eligible for secondary prevention treatment for atrial fibrillation (anticoagulation or left atrial appendage device closure) should undergo a period of prolonged (at least 12 hours) cardiac monitoring.People with ischaemic stroke or TIA who would be eligible for secondary prevention treatment for atrial fibrillation and in whom no other cause of stroke has been found should be considered for more prolonged ECG monitoring (24 hours or longer), particularly if they have a pattern of cerebral ischaemia on brain imaging suggestive of cardioembolism.	2016	20

For peer review only

Table 2. Prognostic ECG findings on extended ECG recording which should be communicated to the local investigator.

Ventricular tachycardia
Excessive ventricular extrasystoles (> 5%)
Pause > 3 seconds
AV-block type II and III
Bradycardia < 40/minute

For peer review only

For peer review only - http://bmjopen.bmj.com/site/about/guidelines.xhtml

**Endpoint* risk in patients with AF and OAC treatment**	**7.3%**
**Endpoint risk in patients with AF and no OAC treatment**	**17%**
**Endpoint risk in patients without AF**	**5%**
**AF detection proportion in standard arm****	**6%**
**AF detection proportion in intervention arm****	**19%**

*Endpoint including recurrent stroke, all-cause mortality, and intracerebral bleeding.**Including AF detection during follow-up.

Table 3. Event rates and atrial fibrillation detection rates used in sample size calculation.

For peer review only

For peer review only - http://bmjopen.bmj.com/site/about/guidelines.xhtml

Table 4a Primary outcome

Variable	Coding ICD-10	Source	Validity
Ischemic stroke	I63	Riksstroke	High
Stroke not specified	I64	Riksstroke	High
All-cause mortality	N/A	National register of death	Very high
Intracerebral bleeding (spontaneous)	I61	Riksstroke	High

Table 4b Secondary outcome

<table>
<tr><th>Variable</th><th colspan="2">Coding ICD-10</th><th>Source</th></tr>
<tr><td>Myocardial infarction</td><td colspan="2">I21, I22, I25.2</td><td rowspan="9">National inpatient register, Riksstroke</td></tr>
<tr><td rowspan="5">Major bleeding</td><td>Gastrointestinal bleeding</td><td>K226, K250, K252, K254, K256, K260, K262, K264, K266, K270, K272, K274, K276, K280, K282, K284, K286, K290, K625, K661, K920, K921, K922, I850, I983</td></tr>
<tr><td>Urogenital bleeding</td><td>N02, R319, N939, N950, N501A</td></tr>
<tr><td>Other bleeding</td><td>H113, H313, H356, H431, H450, H922, I312, J942, M250, R04, R58, T810, D500, D629, T792</td></tr>
<tr><td>Sub-, epidural and subarachnoid haemorrhages (incl traumatic)</td><td>I60, I61, I62, S064, S065, S066</td></tr>
<tr><td>Procedure codes for transfusion</td><td>DR029, DR033, Z513</td></tr>
<tr><td>Atrial fibrillation/flutter</td><td colspan="2">I48</td></tr>
<tr><td>Pacemaker implantation</td><td colspan="2">FPE00, FPE10, FPE20, FPE26</td></tr>
<tr><td>OAC and antiplatelet treatment, NSAID</td><td colspan="2">B01A, N02BA, M01A</td><td>National drug dispense register</td></tr>
</table>

For peer review only

For peer review only - http://bmjopen.bmj.com/site/about/guidelines.xhtml

For peer review only

For peer review only - http://bmjopen.bmj.com/site/about/guidelines.xhtml

650 ## Figure legend

651 Figure 1. Overview of study design AF SPICE.

652

653

For peer review only

For peer review only - http://bmjopen.bmj.com/site/about/guidelines.xhtml

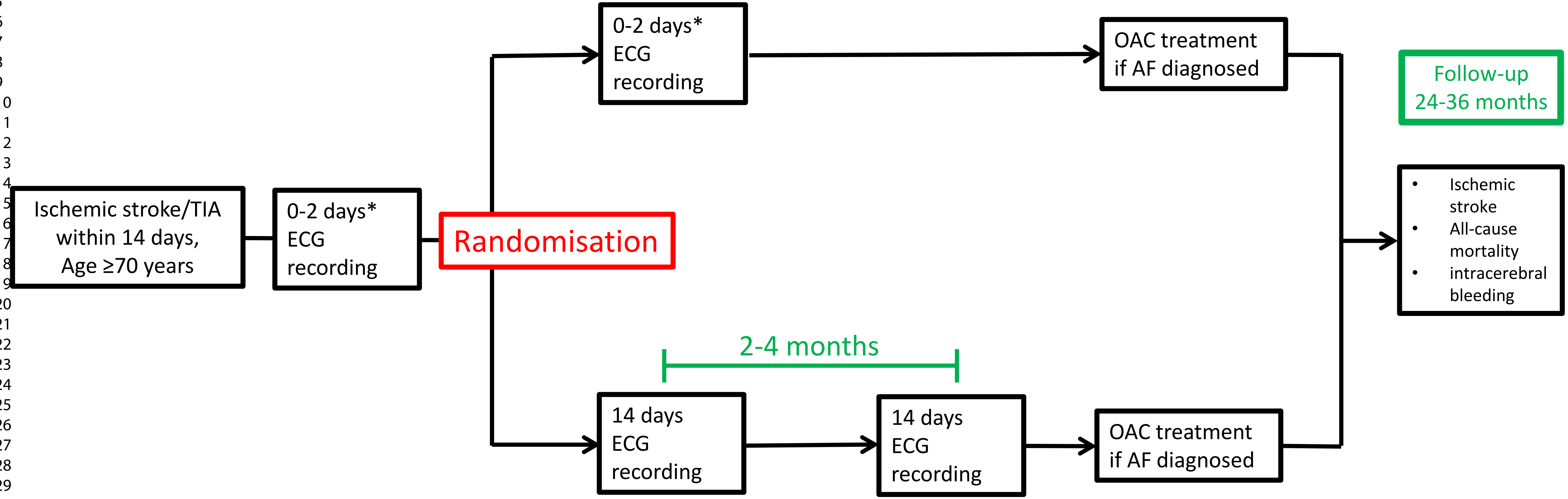

*In total 48 ± 24h ECG recording allowed in control arm.
AF = Atrial fibrillation
OAC = Oral Anticoagulation

Supplementary Appendix

Table of contents

Trials in event rates power calculation 2
Appendix table Trials in ECG detection rate power calculation 3
Baseline data entered at inclusion 4
Baseline data collected from Riksstroke register 5
ECG Data collected by local investigator in standard arm 6
Data collected by local investigator in intervention arm 6
Sample size calculation 13
Patient consent form 16

For peer review only

For peer review only - http://bmjopen.bmj.com/site/about/guidelines.xhtml

Trials in event rates power calculation

	AF+/OAC+			AF+/OAC-			AF-/OAC-		
	Rate	Mean Age	Reference	Rate	Mean Age	Reference	Rate	Mean Age	Reference
**Stroke**	2,23%	70,2	Diener[1] 2010 D110	8,3%	71,7	Diener[2] 2012	5,40%	73	Netland el al 2019
	1,91%	70,8	Diener[1] 2010 D150				5,10%	66	Amarenco[3] 2016
	2,53%	70,4	Diener[1] 2010 W				3,60%	70,1	Andersen[4] 2015
	2,39%	71,7	Diener[2] 2012 A				5,40%		Wachter[5] 2017
	2,79%	71	Hankey[6] 2012 R						
	2,96%	71	Hankey[6] 2012 W						
	2,26%	70,1	Easton[7] 2012 A						
	3,17%	70,1	Easton[7] 2012 W						
	2,44%		Rost[8] 2016 E						
**All-cause mortality**	3,24%	70,2	Diener[1] 2010 D110	7,9%	71,7	Diener[2] 2012	10,70%	73	Netland el al 2019
	4,39%	70,8	Diener[1] 2010 D150				1,80%	66	Amarenco[3] 2016
	4,58%	70,4	Diener[1] 2010 W				10,50%	70,1	Andersen[4] 2015
	5,78%	71,7	Diener[2] 2012 A				4,80%		Wachter[5] 2017
	4,40%	71	Hankey[6] 2012 R				10,60%		Lip[9] 2017
	4,54%	71	Hankey[6] 2012 W						
	4,22%	70,1	Easton[7] 2012 A						
	4,77%	70,1	Easton[7] 2012 W						
	4,35%		Rost[8] 2016 E						
**Intracerebral bleeding**	0,25%	70,2	Diener[1] 2010 D110	1,6%	71,7	Diener[2] 2012	0,40%	66	Amarenco[3] 2016
	0,53%	70,8	Diener[1] 2010 D150						
	1,28%	70,4	Diener[1] 2010 W						
	1,17%	71,7	Diener[2] 2012 A						
	0,59%	71	Hankey[6] 2012 R						
	0,80%	71	Hankey[6] 2012 W						
	0,55%	70,1	Easton[7] 2012 A						
	1,49%	70,1	Easton[7] 2012 W						
	0,62%		Rost[8] 2016 E						
**Mortal bleeding**	0,26%	71	Hankey[6] 2012 R						
	0,49%	71	Hankey[6] 2012 W						

AF = Atrial Fibrillation OAC = Oral Anticoagulant

For peer review only - http://bmjopen.bmj.com/site/about/guidelines.xhtml

Appendix table Trials in ECG detection rate power calculation

Publication Short-term external ECG after stroke/TIA	Detection rate (%)
Jabaudon[10] 2004	5.0
Shafqat[11] 2004	2.4
Alhadramy[12] 2010	2.5
Rizos[13] 2010	2.5
Doliwa-Sobocinski[14] 2012	2.0
Lazarro[15] 2012	6.0
Gumbinger[16] 2012	1.0
Suissa[17] 2012	2.3
Thakkar[18] 2014	5.8
Bansil[19] 2004	4.7

Publication Long-term external ECG after stroke/TIA	Detection rate (%)
Barthelemy[20] 2003	14.3
Jabadoun[10] 2004	5.7
Wallman[21] 2007	14.2
Tayal[22] 2008	5.0
Elijiovich[23] 2009	20.0
Bhatt[24] 2011	24.2
Flint[25] 2012	11.0
Higgins[26] 2013	8.0
Miller[27] 2013	17.3
Gladstone[28] 2014	16.1
Sebasigari[29] 2017	11.7
Kaura[30] 2019	16.3

For peer review only - http://bmjopen.bmj.com/site/about/guidelines.xhtml

For peer review only

Baseline data entered at inclusion

Variable	Unit
Record ID (automatically generated)	
Sex	Male/Female
Date of birth	Date
Social Security Number	Special format
Date of Stroke/TIA	Date
Age	Years
Height	cm
Weight	kg
Hemoglobin	g/l
Estimated GFR	ml/min
Site identification	Choice from list
Consent Date	Date
Randomization date	Date

For peer review only

For peer review only - http://bmjopen.bmj.com/site/about/guidelines.xhtml

Baseline data collected from Riksstroke register

Reporting Hospital	Statin treatment on admission
Date of symptom onset	Aspirin treatment on admission
Time of symptom onset	Clopidogrel treatment on admission
Level of consciousness at admission	
NIHSS at admission	Dipyridamole treatment on admission
Number of days admitted	Other antiplatelet treatment on admission
Number of days in stroke unit	Hypertension treatment on admission
	Dipyridamole treatment on admission
Diagnosis	Warfarin treatment on admission
Age at admission	Apixaban treatment on admission
Sex	Dabigatran treatment on admission
Level of assistance	Rivaroxaban treatment on admission
Living alone	Edoxaban treatment on admission
Level of dependence	
Assisted dressing	Statin treatment at discharge
Assisted personal hygiene	Aspirin treatment at discharge
Independence in locomotion	Clopidogrel treatment at discharge
	Dipyridamole treatment at discharge
Previously diagnosed AF	Warfarin treatment at discharge
AF diagnosed on arrival	Hypertension treatment at discharge
Previously or newly diagnosed diabetes	Apixaban treatment at discharge
Hypertension (treated) on admission	Dabigatran treatment at discharge
Smoking status	Rivaroxaban treatment at discharge
CT brain scan performed during admission	Reason for withholding OAC treatment
MRI brain scan performed during admission	
Carotide artery ultrasound brain scan performed during admission	Edoxaban treatment at discharge
CT angiography performed during admission	
Longterm ECG (at least 24 h) performed during admission	
Thrombolytic treatment administered	
Type of ward at admission	

For peer review only - http://bmjopen.bmj.com/site/about/guidelines.xhtml

ECG Data collected by local investigator in standard arm

Variable	Unit
Inpatient ECG Telemetry used	Yes/No
Duration of ECG Telemetry	Hours
AF diagnosed during ECG Telemetry	Yes/No
Holter Recording used	Yes/No
Duration of Holter Recording	Hours
AF diagnosed during Holter	Yes/No

Data collected by local investigator in intervention arm

Variable	Unit
ePatch number 1 serial number	Integer
Date epatch number 1 recording start	Date
Additional epatch number 1 serial number	Integer
Site of application additional epatch number 1	Health care/self-application
Date for mailing out additional epatch number 1	Date
Date additional epatch number 1 recording start	Date
Reason for additional epatch	1. epatch detachment<br>2. Red light indicated<br>3. Poor signal quality<br>4. MRI examination

For peer review only - http://bmjopen.bmj.com/site/about/guidelines.xhtml

	5. Other radiology<br>6. Thoracic/neck surgery<br>7. Other reason
Additional epatch number 1 serial number	Integer
Site of application additional epatch number 1	Health care/self-application
Date for mailing out additional epatch number 1	Date
Date additional epatch number 1 recording start	Date
Reason for additional epatch number 1	1. epatch detachment<br>2. Red light indicated<br>3. Poor signal quality<br>4. MRI examination<br>5. Other radiology<br>6. Thoracic/neck surgery<br>7. Other reason
Mode of application epatch number 2	Health care/self-application
Date for mailing of epatch number 2 to participant	Date
Date of application epatch number 2	Date
Epatch number 2 serial number	Integer
First additional epatch number 2 serial number	Integer
Mode of application first additional epatch number 2	Health care/self-application
Date for mailing of first additional epatch number 2 to participant	Date
Date of application of first additional epatch number 2	
Reason for first additional epatch number 2	1. epatch detachment<br>2. Red light indicated<br>3. Poor signal quality<br>4. MRI examination<br>5. Other radiology<br>6. Thoracic/neck surgery<br>7. Other reason

For peer review only - http://bmjopen.bmj.com/site/about/guidelines.xhtml

Second additional epatch number 2 serial number	Integer
Mode of application second additional epatch number 2	Health care/self-application
Date for mailing of second additional epatch number 2 to participant	Date
Date of application of second additional epatch number 2	
Reason for second additional epatch number 2	1. epatch detachment<br>2. Red light indicated<br>3. Poor signal quality<br>4. MRI examination<br>5. Other radiology<br>6. Thoracic/neck surgery<br>7. Other reason
Reason for not recording epatch number 2	1. Diagnosed with AF on epatch number 1<br>2. Participant declines recording<br>3. Participant decline further participation<br>4. Other reason

For peer review only

For peer review only - http://bmjopen.bmj.com/site/about/guidelines.xhtml

ECG Data collected in intervention group

Patch number 1	
**Variable**	**Unit**
Date for ECG data upload in Cardiologs	Date
Date for ECG reading	Date
Date for first additional ECG data upload in Cardiologs	Date
Date for first additional ECG reading	Date
Date for second additional ECG data upload in Cardiologs	Date
Date for second additional ECG reading	Date
Monitoring time	Hours
Analyzable time	Hours
Heart rate max	Bpm
Heart rate min	Bpm
Heart rate mean	Bpm
Proportion supraventricular ectopics	Percent
Proportion ventricular ectopics	Percent
Number of pauses > 2 seconds	Integer
Longest pause	Seconds, one decimal
AV-block II or III	Yes/No
Highest AV-block grade	1. AV-block II type 1<br>2. AV-block II type 2<br>3. AV-block III
Atrial fibrillation	Yes/No
Atrial flutter	Yes/No
Duration to first Atrial fibrillation/flutter	Hours
Atrial fibrillation/flutter subtype	1. Paroxysmal<br>2. Persistant
Atrial fibrillation/ flutter heart rate max	Bpm

For peer review only - http://bmjopen.bmj.com/site/about/guidelines.xhtml

Atrial fibrillation/ flutter heart rate mean	Bpm
Atrial fibrillation/ flutter burden	Percent
Atrial fibrillation/ flutter longest episode	Seconds
Number of Ventricular Tachycardia (VT)	n
Duration longest episode VT	Seconds
Number of Suptraventricular Tachycardia (SVT)	n
Duration longest episode SVT	Seconds
Proportion of noise	Percent
Action triggered by recording	1. Standard statement<br>2. Communication with local PI within 3 working days<br>3. Communication with local PI within one working day<br>4. Further assessment following patch number 2
**Patch number 2**	
**Variable**	**Unit**
Date for ECG data upload in Cardiologs	Date
Date for ECG reading	Date
Date for first additional ECG data upload in Cardiologs	Date
Date for first additional ECG reading	Date
Date for second additional ECG data upload in Cardiologs	Date
Date for second additional ECG reading	Date
Monitoring time	Hours
Analyzable time	Hours
Heart rate max	Bpm
Heart rate min	Bpm

For peer review only - http://bmjopen.bmj.com/site/about/guidelines.xhtml

For peer review only

Heart rate mean	Bpm
Proportion supraventricular ectopics	Percent
Proportion ventricular ectopics	Percent
Number of pauses > 2 seconds	Integer
Longest pause	Seconds, one decimal
AV-block II or III	Yes/No
Highest AV-block grade	1. AV-block II type 1<br>2. AV-block II type 2<br>3. AV-block III
Atrial fibrillation	Yes/No
Atrial flutter	Yes/No
Atrial fibrillation/flutter subtype	1. Paroxysmal<br>2. Persistant
Atrial fibrillation/ flutter heart rate max	Bpm
Atrial fibrillation/ flutter heart rate mean	Bpm
Atrial fibrillation/ flutter burden	Percent
Atrial fibrillation/ flutter longest episode	Seconds
Number of Ventricular Tachycardia (VT)	n
Duration longest episode VT	Seconds
Number of Suptraventricular Tachycardia (SVT)	n
Duration longest episode SVT	Seconds
Proportion of noise	Percent
Action triggered by recording	1. Standard statement<br>2. Communication with local PI within 3 working days<br>3. Communication with local PI within one working day

For peer review only

	4. Further assessment following patch number 2

For peer review only

For peer review only - http://bmjopen.bmj.com/site/about/guidelines.xhtml

Sample size calculation

Sample size calculation AF SPICE

Ida Hed Myrberg & Letizia Orsini

2021-10-08

Background

Sample size calculation for the AF SPICE study; a multicenter randomized register-based trail in Swedish stroke units.

Method

The function *cpower* in R package *Hmisc* is used. This is a function for calculating the power for a Cox Proportional Hazards model or log-rank test comparing two groups. The function assumes an underlying exponential distribution in both groups. To calculate the sample size needed to achieve 80% power, the function *uniroot* is used in combination with *cpower*.

How parameters are defined and calculated

In this section, it is shown how the yearly rate, and the 3-year event rate, of stroke/death/intracranial bleeding in the intervention and control group, respectively, are calculated based on the yearly rates in the three groups AF+/OAK+, AF+/OAK-, and AF-/OAK-, and on the proportions of these three groups in the intervention and control group. The proportion of AF+/OAK+, AF+/OAK-, and AF-/OAK- in the intervention and control group in Table 2 are examples, and in the next two sections they will be varied between 10-20% in the intervention group, and 3-12% in the control group. The yearly rate of stroke/death/intracranial bleeding for AF-/OAK- will be set to 5% and 8.4%, respectively, in two different scenarios.

Table 1 shows the assumed yearly rates of stroke/death/intracranial bleeding for the subgroups AF+/OAK+, AF+/OAK-, AF-/OAK-, while Table 2 shows the proportion of patients in each of the aforementioned subgroups, divided by interention and control.

Table 1: Yearly rate of stroke/death/intracranial bleeding depending on AF and treatment.

AF/treatment	Yearly rate of stroke/death/intracranial bleeding
AF+/OAK+	0.073
AF+/OAK-	0.177
AF-/OAK-	0.084

Table 2: Proportion of patients with AF and treatment in the intervention group and control group, respectively.

AF/treatment	Proportion in intervention group	Proportion in control group
AF+/OAK+	0.10	0.03
AF+/OAK-	0.00	0.07
AF-/OAK-	0.90	0.90

The yearly rate of stroke/death/intracranial in the intervention group is calculated as 0.073 * 0.1 + 0.177 * 0 + 0.084 * 0.9 = 0.0829. The yearly rate of stroke/death/intracranial in the control group is calculated as 0.073 * 0.03 + 0.177 * 0.07 + 0.084 * 0.9 = 0.09018.

Table 3: Yearly rate of stroke/death/intracranial bleeding in the intervention group and control group, respectively.

Group	Yearly rate of stroke/death/intracranial bleeding
Intervention group	0.0829
Control group	0.0902

The minimum follow-up time is assumed to be 3 years, and the recruitment time is set to 2 years.

The 3-year event rate in the control group, denoted *mc*, i.e. the proportion expected to have experienced the event by three years, is needed for the power calculation. The proportion not having experienced the event, i.e. 1-*mc*, is calculated using the Kaplan-Meier method, and the 3-year event rate is then calculated as 1-(1-*mc*)=*mc*. The 3-year event rate in the intervention group, *mi*, is calculated in a similar way. Both *mc* and *mi* are presented in Table 4.

Table 4: 3-year rate of stroke/death/intracranial bleeding in the intervention group and control group, respectively.

Group	3-year rate of stroke/death/intracranial bleeding
Intervention group	0.2287
Control group	0.2469

The percent reduction in the 3-year event rate for the intervention group compared to the control group is given by 1-22.87/24.69=7.38%.

Results: 5% yearly rate of stroke/death/intracranial bleeding for AF-/OAK-

In this section, a yearly event rate of 5% is assumed for the AF-/OAK- group.

The sample size needed is calculated for all combination of AF proportions, ranging from 10-20% in the intervention group, and 3-12% in the control group. Table 5 shows the percent reduction in the 3-year event rate for the intervention group compared to the control group, for different combinations of AF proportions.

Table 5: Percent reduction in the 3-year event rate for the intervention group compared to the control group. Columns: AF proportions in the control group. Rows: AF proportions in the intervention group.

	0.03	0.04	0.05	0.06	0.07	0.08	0.09	0.1	0.11	0.12
0.1	11.57	10.09	8.56	6.97	5.33	3.62	1.85	-	-	-
0.11	12.94	11.52	10.05	8.52	6.94	5.31	3.60	1.84	-	-

For peer review only - http://bmjopen.bmj.com/site/about/guidelines.xhtml

	0.03	0.04	0.05	0.06	0.07	0.08	0.09	0.1	0.11	0.12
0.12	14.25	12.89	11.47	10.01	8.49	6.92	5.28	3.59	1.83	-
0.13	15.52	14.20	12.83	11.42	9.96	8.45	6.89	5.26	3.57	1.82
0.14	16.73	15.46	14.14	12.78	11.38	9.92	8.42	6.86	5.24	3.56
0.15	17.89	16.66	15.40	14.09	12.73	11.33	9.88	8.38	6.83	5.21
0.16	19.01	17.82	16.60	15.34	14.03	12.68	11.29	9.84	8.35	6.80
0.17	20.09	18.94	17.76	16.54	15.28	13.98	12.63	11.24	9.80	8.31
0.18	21.12	20.01	18.87	17.69	16.48	15.22	13.92	12.58	11.20	9.76
0.19	22.12	21.05	19.94	18.80	17.63	16.41	15.16	13.87	12.53	11.15
0.2	23.08	22.04	20.97	19.87	18.73	17.56	16.35	15.10	13.82	12.48

Table 6 shows the total sample size needed to achieve 80% power to detect a statistically significant (with alpha=0.05) difference in the 3-year event rates presented in Table 5 between the intervention group and the control group, for different combinations of AF proportions.

Table 6: Total sample size needed for different combinations of AF proportions in the intervention and control group, respectively. Columns: AF proportions in the control group. Rows: AF proportions in the intervention group.

	0.03	0.04	0.05	0.06	0.07	0.08	0.09	0.1	0.11	0.12
0.1	8536	11504	16400	25370	44652	99460	393830	-	-	-
0.11	6628	8572	11552	16470	25480	44848	99900	395590	-	-
0.12	5310	6654	8608	11600	16542	25590	45044	100340	397348	-
0.13	4360	5332	6682	8644	11650	16612	25700	45240	100780	399108
0.14	3652	4378	5352	6710	8680	11698	16682	25810	45436	101220
0.15	3110	3668	4396	5374	6738	8716	11748	16752	25922	45630
0.16	2686	3124	3682	4412	5396	6764	8752	11796	16824	26032
0.17	2346	2696	3136	3696	4430	5418	6792	8788	11846	16894
0.18	2072	2356	2708	3148	3710	4448	5440	6820	8824	11894
0.19	1844	2080	2364	2718	3160	3726	4466	5462	6848	8860
0.2	1654	1850	2086	2374	2728	3172	3740	4484	5484	6876

Results: 8.4% yearly rate of stroke/death/intracranial bleeding for AF-/OAK-

In this section, a yearly event rate of 8.4% is assumed for the AF-/OAK- group.

The sample size needed is calculated for all combination of AF proportions, ranging from 10-20% in the intervention group, and 3-12% in the control group. Table 7 shows the percent reduction in the 3-year event rate for the intervention group compared to the control group, for different combinations of AF proportions.

Table 7: Percent reduction in the 3-year event rate for the intervention group compared to the control group. Columns: AF proportions in the control group. Rows: AF proportions in the intervention group.

	0.03	0.04	0.05	0.06	0.07	0.08	0.09	0.1	0.11	0.12
0.1	7.38	6.40	5.40	4.37	3.32	2.24	1.13	-	-	-
0.11	8.35	7.39	6.41	5.41	4.38	3.32	2.24	1.13	-	-

	0.03	0.04	0.05	0.06	0.07	0.08	0.09	0.1	0.11	0.12
0.12	9.30	8.36	7.40	6.42	5.41	4.38	3.33	2.25	1.14	-
0.13	10.23	9.31	8.37	7.41	6.43	5.42	4.39	3.33	2.25	1.14
0.14	11.14	10.24	9.32	8.38	7.42	6.44	5.43	4.39	3.34	2.25
0.15	12.04	11.16	10.26	9.34	8.40	7.43	6.45	5.44	4.40	3.34
0.16	12.91	12.05	11.17	10.27	9.35	8.41	7.44	6.45	5.44	4.41
0.17	13.78	12.93	12.07	11.19	10.28	9.36	8.42	7.45	6.46	5.45
0.18	14.62	13.79	12.95	12.08	11.20	10.30	9.37	8.43	7.46	6.47
0.19	15.45	14.64	13.81	12.96	12.10	11.22	10.31	9.39	8.44	7.47
0.2	16.26	15.47	14.66	13.83	12.98	12.12	11.23	10.33	9.40	8.45

Table 8 shows the total sample size needed to achieve 80% power to detect a statistically significant (with alpha=0.05) difference in the 3-year event rates presented in Table 5 between the intervention group and the control group, for different combinations of AF proportions.

Table 8: Total sample size needed for different combinations of AF proportions in the intervention and control group, respectively. Columns: AF proportions in the control group. Rows: AF proportions in the intervention group.

	0.03	0.04	0.05	0.06	0.07	0.08	0.09	0.1	0.11	0.12
0.1	13380	18096	25892	40198	71006	158734	630838	-	-	-
0.11	10296	13362	18072	25858	40144	70910	158518	629976	-	-
0.12	8176	10282	13346	18048	25824	40090	70814	158302	629114	-
0.13	6656	8166	10270	13328	18024	25790	40036	70718	158088	628250
0.14	5530	6648	8156	10256	13310	18000	25754	39982	70622	157872
0.15	4670	5522	6640	8144	10242	13292	17976	25720	39928	70526
0.16	3998	4664	5514	6630	8134	10228	13274	17952	25686	39874
0.17	3466	3994	4658	5508	6622	8124	10216	13258	17928	25652
0.18	3034	3460	3988	4652	5500	6614	8112	10202	13240	17904
0.19	2680	3030	3456	3984	4646	5494	6604	8102	10188	13222
0.2	2386	2676	3026	3452	3978	4640	5486	6596	8092	10174

Selected sample size

To calculate the expected cases in the intervention group and the control group, the function *cpower* was run with the following parameters:

- accrual time 2 years;
- follow-up time 3 years;
- aplha 0.05;
- power 0.8;
- 5% yearly rate of stroke/death/intracranial bleeding for AF-/OAK-;
- 19% AF proportions in the intervention group;
- 6% AF proportions in the control group.

The result of *cpower* is:

```
Accrual duration: 2 y  Minimum follow-up: 3 y
```

Biostatistics
CORE FACILITY

```
Total sample size: 2718

Alpha= 0.05

3-year Mortalities
     Control Intervention
   0.1901558    0.1544025

Hazard Rates
     Control Intervention
  0.07030447   0.05590393

Probabilities of an Event During Study
     Control Intervention
   0.2445141    0.1999611

Expected Number of Events
     Control Intervention
       332.3        271.7

Hazard ratio: 0.7951689
Standard deviation of log hazard ratio: 0.08178795
    Power
0.8002227
```

In the first two years, you have to enroll uniformly over time 1359 patients in the control group and 1359 in the intervention group. After the first two years, you have to follow them up for another three years. At least five years after the start of the study, you must check the observed cases in the control group and in the intervention group. If the study did not reach 333 cases in the control group and 272 in the intervention group, the follow-up can be extended until these are reached. This minimum number of cases guarantees a power of 80%.

For peer review only

Patient consent form

Patientnummer:________________

Bilaga 05

Randomiserad jämförelse mellan standardiserad och utökad EKG-undersökning efter stroke eller TIA för att detektera förmaksflimmer och minska risken för stroke - AF SPICE

Forskningspersonsformation och samtyckesformulär

För närvarande pågår i Sverige en vetenskaplig studie som går ut på att studera två olika metoder att upptäcka förmaksflimmer hos personer som drabbats av slaganfall (stroke) eller TIA (övergående stroke). Det är orsaken till att du får denna information och förfrågan. Studien drivs av Karolinska Institutet.

Varför görs detta forskningsprojekt?

Förmaksflimmer är den vanligaste rubbningen i hjärtrytmen och förekomsten ökar med stigande ålder. Tillståndet ger oregelbunden hjärtrytm och symptomen varierar kraftigt, i vissa fall kan man ha förmaksflimmer utan några symptom alls.

Vid förmaksflimmer finns en ökad risk att drabbas av stroke, detta beror på en ökad risk för att det bildas blodproppar i hjärtats förmak, dessa proppar kan sedan följa med blodet till hjärnan och därmed orsaka stroke eller TIA. Risken för stroke vid förmaksflimmer kan reduceras med 60-70% med blodförtunnande behandling. Blodförtunnande behandling innebär också en viss ökning av risken att drabbas av allvarlig blödning.

För de som drabbats av stroke eller TIA är det angeläget att ta reda på om individen har förmaksflimmer. Om så är fallet finns det starka skäl att starta en särskild sorts blodförtunnande behandling, så kallad *antikoagulantia*. Utredning för att hitta förmaksflimmer efter stroke eller TIA görs med olika typer av EKG-undersökningar, ofta över en eller flera dygn. Det finns ännu inte någon forskning som visar på vilken typ av EKG-undersökning som är den bästa efter stroke, vilket också har lett till att praxis varierar. Långa EKG-undersökningar tar också stora resurser i anspråk varför det är angeläget att ta reda på den medicinska nyttan med olika metoder.

I detta forskningsprojekt kommer du att lottas till ett av två alternativ: standardutredning eller utökad utredning. Standardutredningen består av 24-48 timmars EKG-utredning och är den utredning som rekommenderas starkast av Socialstyrelsen. Utökad utredning består av långtids-EKG med så kallat plåster-EKG under 14 dagar vid två tillfällen.

Studien är godkänd av Etikprövningsmyndigheten.

Vad innebär det för mig att delta i studien?

För dig som lottas till **standardutredning** kommer EKG-undersökning genomföras inneliggande på avdelning eller som långtids-EKG med portabel utrustning i hemmet.

För dig som lottas till **utökad utredning** kommer EKG-undersökning med plåster-EKG starta på avdelningen och oftast avslutas efter utskrivning då du sänder tillbaka EKG-plåstret med

Patientnummer:________________

Bilaga 05

Randomiserad jämförelse mellan standardiserad och utökad EKG-undersökning efter stroke eller TIA för att detektera förmaksflimmer och minska risken för stroke - AF SPICE

ett medsänt svarskuvert. Du kommer också att i samband med ett återbesök påbörja ytterligare en 14-dagars EKG-registrering med plåster-EKG där du sänder tillbaka utrustningen på samma sätt.

Om EKG i någon av undersökningarna visar på förmaksflimmer kommer du utan fördröjning att kontaktas av läkare för att diskutera eventuell ändring av din blodförtunnande behandling.

Därefter görs en långtidsuppföljning under minst tre år där Socialstyrelsens register samt registret Riks-stroke kommer att studeras för att bland annat följa förekomsten av insjuknanden i stroke, förskrivning av proppförebyggande läkemedel och överlevnad. Denna uppföljning kräver ingen aktiv medverkan från dig. De register från Socialstyrelsen som kommer att användas är patientregistret, läkemedelsregistret och dödsorsaksregistret.

Ersättning för deltagande och resor utgår inte.

Vilka obehag/risker medför studien?

Både förmaksflimmer och blodförtunnande behandling gör att man är utsatt för vissa risker, men den riskminskning som åstadkommes med blodförtunnande behandling är i de allra flesta fall flerfalt större än den risk som behandlingen innebär. Denna behandling rekommenderas också i Socialstyrelsens senaste nationella riktlinjer för hjärtsjukvård.

Undersökning med långtids-EKG och plåster-EKG kan i vissa fall ge hudirritation där utrustningen ansluts på huden. Dessa besvär är ofta lindriga och alltid övergående.

Vilka fördelar kan studien innebära för dig?

Ditt deltagande ger en möjlighet att upptäcka och behandla ett tillstånd som ökar risken för ny stroke och TIA, dessutom kan studien ge oss värdefulla kunskaper som även andra kan ha nytta av i framtiden

Hur insamlas personuppgifter och hur hanteras sekretess?

Dina svar och dina resultat kommer att behandlas så att inte obehöriga kan ta del av dem. Ansvarig för dina personuppgifter är Region Stockholm. Enligt EU:s dataskyddsförordning (GDPR 2016/679) har du rätt att kostnadsfritt få ta del av de uppgifter om dig som hanteras i studien, och vid behov få eventuella fel rättade. Du kan också begära att uppgifter om dig raderas samt att behandlingen av dina personuppgifter begränsas. Om du vill ta del av uppgifterna ska du kontakta personuppgiftsombudet på dso.ds@sll.se [alt mailadress för aktuellt site] eller ansvarig forskare. Om du är missnöjd med hur dina personuppgifter behandlas har du rätt att ge in klagomål till Integritetsskyddsmyndigheten, som är tillsynsmyndighet.

Patientnummer:________________

Bilaga 05

Randomiserad jämförelse mellan standardiserad och utökad EKG-undersökning efter stroke eller TIA för att detektera förmaksflimmer och minska risken för stroke - AF SPICE

Är deltagandet i studien frivilligt?
Deltagandet i denna studie är frivilligt, kostnadsfritt och du har när som helst rätt att avbryta ditt deltagande utan att ange skäl och utan att det påverkar ditt framtida omhändertagande. Kostnad för eventuell läkemedelsbehandling efter ditt deltagande omfattas av sedvanlig läkemedelsförmån.

Kontaktpersoner

Om du vill ha ytterligare information om studien, dina rättigheter som patient eller om problem uppstår under studien ska du vända dig till:

Ansvarig prövare på stroke-avdelningen: XXXX XXXX

För studien ansvarig läkare:

Johan Engdahl, Överläkare/Docent
Karolinska Institutet, Danderyds Sjukhus
Hjärtkliniken
Danderyds Sjukhus
182 57 Danderyd
Johan.engdahl@sll.se

Patientnummer:________________

Bilaga 05

Randomiserad jämförelse mellan standardiserad och utökad EKG-undersökning efter stroke eller TIA för att detektera förmaksflimmer och minska risken för stroke - AF SPICE

Samtycke till att delta i studien

Jag har fått muntlig och skriftlig informationen om studien och har haft möjlighet att ställa frågor. Jag får behålla den skriftliga informationen.

☐ Jag samtycker till att delta i studien *Randomiserad jämförelse mellan standardiserad och utökad EKG-undersökning efter stroke eller TIA för att detektera förmaksflimmer och minska risken för stroke - AF SPICE*

☐ Jag samtycker till att uppgifter om mig behandlas på det sätt som beskrivs i forskningspersonsinformationen.

☐ Jag samtycker till att uppgifter om mig ur de olika register som beskrivs ovan utförs som långtidsuppföljning

Ort Datum ____________________

____

Forskningspersons namnteckning

____

Namnförtydligande

Jag har förklarat studiens upplägg och syfte, samt inhämtat samtycke för deltagande:

Ort Datum ____________________

____

Namnteckning prövare

____

Namnförtydligande

References

1. Diener HC, Connolly SJ, Ezekowitz MD, Wallentin L, Reilly PA, Yang S, Xavier D, Di Pasquale G and Yusuf S. Dabigatran compared with warfarin in patients with atrial fibrillation and previous transient ischaemic attack or stroke: a subgroup analysis of the RE-LY trial. *Lancet Neurol*. 2010;9:1157-63.

2. Diener HC, Eikelboom J, Connolly SJ, Joyner CD, Hart RG, Lip GY, O'Donnell M, Hohnloser SH, Hankey GJ, Shestakovska O, Yusuf S, Committee AS and Investigators. Apixaban versus aspirin in patients with atrial fibrillation and previous stroke or transient ischaemic attack: a predefined subgroup analysis from AVERROES, a randomised trial. *Lancet Neurol*. 2012;11:225-31.

3. Amarenco P, Lavallée PC, Labreuche J, Albers GW, Bornstein NM, Canhão P, Caplan LR, Donnan GA, Ferro JM, Hennerici MG, Molina C, Rothwell PM, Sissani L, Školoudík D, Steg PG, Touboul PJ, Uchiyama S, Vicaut É and Wong LK. One-Year Risk of Stroke after Transient Ischemic Attack or Minor Stroke. *N Engl J Med*. 2016;374:1533-42.

4. Andersen SD, Gorst-Rasmussen A, Lip GY, Bach FW and Larsen TB. Recurrent Stroke: The Value of the CHA2DS2VASc Score and the Essen Stroke Risk Score in a Nationwide Stroke Cohort. *Stroke*. 2015;46:2491-7.

5. Wachter R, Groschel K, Gelbrich G, Hamann GF, Kermer P, Liman J, Seegers J, Wasser K, Schulte A, Jurries F, Messerschmid A, Behnke N, Groschel S, Uphaus T, Grings A, Ibis T, Klimpe S, Wagner-Heck M, Arnold M, Protsenko E, Heuschmann PU, Conen D, Weber-Kruger M, Find AFI and Coordinators. Holter-electrocardiogram-monitoring in patients with acute ischaemic stroke (Find-AFRANDOMISED): an open-label randomised controlled trial. *Lancet Neurol*. 2017;16:282-290.

6. Hankey GJ, Patel MR, Stevens SR, Becker RC, Breithardt G, Carolei A, Diener HC, Donnan GA, Halperin JL, Mahaffey KW, Mas JL, Massaro A, Norrving B, Nessel CC, Paolini JF, Roine RO, Singer DE, Wong L, Califf RM, Fox KA, Hacke W and Investigators RASC. Rivaroxaban compared with warfarin in patients with atrial fibrillation and previous stroke or transient ischaemic attack: a subgroup analysis of ROCKET AF. *Lancet Neurol*. 2012;11:315-22.

For peer review only - http://bmjopen.bmj.com/site/about/guidelines.xhtml

7. Easton JD, Lopes RD, Bahit MC, Wojdyla DM, Granger CB, Wallentin L, Alings M, Goto S, Lewis BS, Rosenqvist M, Hanna M, Mohan P, Alexander JH, Diener HC, Committees A and Investigators. Apixaban compared with warfarin in patients with atrial fibrillation and previous stroke or transient ischaemic attack: a subgroup analysis of the ARISTOTLE trial. *Lancet Neurol*. 2012;11:503-11.

8. Rost NS, Giugliano RP, Ruff CT, Murphy SA, Crompton AE, Norden AD, Silverman S, Singhal AB, Nicolau JC, SomaRaju B, Mercuri MF, Antman EM, Braunwald E and Investigators EA-T. Outcomes With Edoxaban Versus Warfarin in Patients With Previous Cerebrovascular Events: Findings From ENGAGE AF-TIMI 48 (Effective Anticoagulation With Factor Xa Next Generation in Atrial Fibrillation-Thrombolysis in Myocardial Infarction 48). *Stroke*. 2016;47:2075-82.

9. Lip GY, Hunter TD, Quiroz ME, Ziegler PD and Turakhia MP. Atrial Fibrillation Diagnosis Timing, Ambulatory ECG Monitoring Utilization, and Risk of Recurrent Stroke. *Circ Cardiovasc Qual Outcomes*. 2017;10.

10. Jabaudon D, Sztajzel J, Sievert K, Landis T and Sztajzel R. Usefulness of ambulatory 7-day ECG monitoring for the detection of atrial fibrillation and flutter after acute stroke and transient ischemic attack. *Stroke*. 2004;35:1647-51.

11. Shafqat S, Kelly PJ and Furie KL. Holter monitoring in the diagnosis of stroke mechanism. *Intern Med J*. 2004;34:305-9.

12. Alhadramy O, Jeerakathil TJ, Majumdar SR, Najjar E, Choy J and Saqqur M. Prevalence and predictors of paroxysmal atrial fibrillation on Holter monitor in patients with stroke or transient ischemic attack. *Stroke*. 2010;41:2596-600.

13. Rizos T, Rasch C, Jenetzky E, Hametner C, Kathoefer S, Reinhardt R, Hepp T, Hacke W and Veltkamp R. Detection of paroxysmal atrial fibrillation in acute stroke patients. *Cerebrovasc Dis*. 2010;30:410-7.

14. Doliwa Sobocinski P, Anggardh Rooth E, Frykman Kull V, von Arbin M, Wallen H and Rosenqvist M. Improved screening for silent atrial fibrillation after ischaemic stroke. *Europace*. 2012;14:1112-6.

For peer review only - http://bmjopen.bmj.com/site/about/guidelines.xhtml

15. Lazzaro MA, Krishnan K and Prabhakaran S. Detection of atrial fibrillation with concurrent holter monitoring and continuous cardiac telemetry following ischemic stroke and transient ischemic attack. *J Stroke Cerebrovasc Dis*. 2012;21:89-93.

16. Gumbinger C, Krumsdorf U, Veltkamp R, Hacke W and Ringleb P. Continuous monitoring versus HOLTER ECG for detection of atrial fibrillation in patients with stroke. *Eur J Neurol*. 2012;19:253-7.

17. Suissa L, Lachaud S and Mahagne MH. Optimal Timing and Duration of Continuous Electrocardiographic Monitoring for Detecting Atrial Fibrillation in Stroke Patients. *J Stroke Cerebrovasc Dis*. 2012.

18. Thakkar S and Bagarhatta R. Detection of paroxysmal atrial fibrillation or flutter in patients with acute ischemic stroke or transient ischemic attack by Holter monitoring. *Indian Heart J*. 2014;66:188-92.

19. Bansil S and Karim H. Detection of atrial fibrillation in patients with acute stroke. *J Stroke Cerebrovasc Dis*. 2004;13:12-5.

20. Barthelemy JC, Feasson-Gerard S, Garnier P, Gaspoz JM, Da Costa A, Michel D and Roche F. Automatic cardiac event recorders reveal paroxysmal atrial fibrillation after unexplained strokes or transient ischemic attacks. *Ann Noninvasive Electrocardiol*. 2003;8:194-9.

21. Wallmann D, Tuller D, Wustmann K, Meier P, Isenegger J, Arnold M, Mattle HP and Delacretaz E. Frequent atrial premature beats predict paroxysmal atrial fibrillation in stroke patients: an opportunity for a new diagnostic strategy. *Stroke*. 2007;38:2292-4.

22. Tayal AH, Tian M, Kelly KM, Jones SC, Wright DG, Singh D, Jarouse J, Brillman J, Murali S and Gupta R. Atrial fibrillation detected by mobile cardiac outpatient telemetry in cryptogenic TIA or stroke. *Neurology*. 2008;71:1696-701.

23. Elijovich L, Josephson SA, Fung GL and Smith WS. Intermittent atrial fibrillation may account for a large proportion of otherwise cryptogenic stroke: a study of 30-day cardiac event monitors. *J Stroke Cerebrovasc Dis*. 2009;18:185-9.

For peer review only - http://bmjopen.bmj.com/site/about/guidelines.xhtml

For peer review only

24. Bhatt A, Majid A, Razak A, Kassab M, Hussain S and Safdar A. Predictors of occult paroxysmal atrial fibrillation in cryptogenic strokes detected by long-term noninvasive cardiac monitoring. *Stroke Res Treat*. 2011;2011:172074.

25. Flint AC, Banki NM, Ren X, Rao VA and Go AS. Detection of paroxysmal atrial fibrillation by 30-day event monitoring in cryptogenic ischemic stroke: the Stroke and Monitoring for PAF in Real Time (SMART) Registry. *Stroke*. 2012;43:2788-90.

26. Higgins P, MacFarlane PW, Dawson J, McInnes GT, Langhorne P and Lees KR. Noninvasive cardiac event monitoring to detect atrial fibrillation after ischemic stroke: a randomized, controlled trial. *Stroke*. 2013;44:2525-31.

27. Miller DJ, Khan MA, Schultz LR, Simpson JR, Katramados AM, Russman AN and Mitsias PD. Outpatient cardiac telemetry detects a high rate of atrial fibrillation in cryptogenic stroke. *J Neurol Sci*. 2013;324:57-61.

28. Gladstone DJ, Spring M, Dorian P, Panzov V, Thorpe KE, Hall J, Vaid H, O'Donnell M, Laupacis A, Cote R, Sharma M, Blakely JA, Shuaib A, Hachinski V, Coutts SB, Sahlas DJ, Teal P, Yip S, Spence JD, Buck B, Verreault S, Casaubon LK, Penn A, Selchen D, Jin A, Howse D, Mehdiratta M, Boyle K, Aviv R, Kapral MK and Mamdani M. Atrial fibrillation in patients with cryptogenic stroke. *N Engl J Med*. 2014;370:2467-77.

29. Sebasigari D, Merkler A, Guo Y, Gialdini G, Kummer B, Hemendinger M, Song C, Chu A, Cutting S, Silver B, Elkind MSV, Kamel H, Furie KL and Yaghi S. Biomarkers of Atrial Cardiopathy and Atrial Fibrillation Detection on Mobile Outpatient Continuous Telemetry After Embolic Stroke of Undetermined Source. *J Stroke Cerebrovasc Dis*. 2017;26:1249-1253.

30. Kaura A, Sztriha L, Chan FK, Aeron-Thomas J, Gall N, Piechowski-Jozwiak B, Teo JT. Early prolonged ambulatory cardiac monitoring in stroke (EPACS): an open-label randomised controlled trial. *Eur J Med Res.* 2019;24(1):25.

For peer review only

SPIRIT 2013 Checklist: Recommended items to address in a clinical trial protocol and related documents*

Section/item	ItemNo	Description	Comment/finished
**Administrative information**			
Title	1	Descriptive title identifying the study design, population, interventions, and, if applicable, trial acronym	Page 1
Trial registration	2a	Trial identifier and registry name. If not yet registered, name of intended registry	Page 4
	2b	All items from the World Health Organization Trial Registration Data Set	
Protocol version	3	Date and version identifier	Page 4
Funding	4	Sources and types of financial, material, and other support	Page 24
Roles and responsibilities	5a	Names, affiliations, and roles of protocol contributors	Page 24
	5b	Name and contact information for the trial sponsor	Page 4

For peer review only - http://bmjopen.bmj.com/site/about/guidelines.xhtml

	5c	Role of study sponsor and funders, if any, in study design; collection, management, analysis, and interpretation of data; writing of the report; and the decision to submit the report for publication, including whether they will have ultimate authority over any of these activities	
	5d	Composition, roles, and responsibilities of the coordinating centre, steering committee, endpoint adjudication committee, data management team, and other individuals or groups overseeing the trial, if applicable (see Item 21a for data monitoring committee)	
**Introduction**			
Background and rationale	6a	Description of research question and justification for undertaking the trial, including summary of relevant studies (published and unpublished) examining benefits and harms for each intervention	Page 6-8
	6b	Explanation for choice of comparators	Page 8
Objectives	7	Specific objectives or hypotheses	
Trial design	8	Description of trial design including type of trial (eg, parallel group, crossover, factorial, single group), allocation ratio, and framework (eg, superiority, equivalence, noninferiority, exploratory)	Page 9

For peer review only - http://bmjopen.bmj.com/site/about/guidelines.xhtml

Methods: Participants, interventions, and outcomes

Study setting	9	Description of study settings (eg, community clinic, academic hospital) and list of countries where data will be collected. Reference to where list of study sites can be obtained	Page 10
Eligibility criteria	10	Inclusion and exclusion criteria for participants. If applicable, eligibility criteria for study centres and individuals who will perform the interventions (eg, surgeons, psychotherapists)	Page 10-11
Interventions	11a	Interventions for each group with sufficient detail to allow replication, including how and when they will be administered	Page 12-15
	11b	Criteria for discontinuing or modifying allocated interventions for a given trial participant (eg, drug dose change in response to harms, participant request, or improving/worsening disease)	Page 11
	11c	Strategies to improve adherence to intervention protocols, and any procedures for monitoring adherence (eg, drug tablet return, laboratory tests)	
	11d	Relevant concomitant care and interventions that are permitted or prohibited during the trial	

For peer review only - http://bmjopen.bmj.com/site/about/guidelines.xhtml

Outcomes	12	Primary, secondary, and other outcomes, including the specific measurement variable (eg, systolic blood pressure), analysis metric (eg, change from baseline, final value, time to event), method of aggregation (eg, median, proportion), and time point for each outcome. Explanation of the clinical relevance of chosen efficacy and harm outcomes is strongly recommended	Page 15
Participant timeline	13	Time schedule of enrolment, interventions (including any run-ins and washouts), assessments, and visits for participants. A schematic diagram is highly recommended (see Figure)	Page 15
Sample size	14	Estimated number of participants needed to achieve study objectives and how it was determined, including clinical and statistical assumptions supporting any sample size calculations	Page 16-17
Recruitment	15	Strategies for achieving adequate participant enrolment to reach target sample size	Page 17

Methods: Assignment of interventions (for controlled trials)

Allocation:

For peer review only - http://bmjopen.bmj.com/site/about/guidelines.xhtml

Sequence generation	16a	Method of generating the allocation sequence (eg, computer-generated random numbers), and list of any factors for stratification. To reduce predictability of a random sequence, details of any planned restriction (eg, blocking) should be provided in a separate document that is unavailable to those who enrol participants or assign interventions	Page 17
Allocation concealment mechanism	16b	Mechanism of implementing the allocation sequence (eg, central telephone; sequentially numbered, opaque, sealed envelopes), describing any steps to conceal the sequence until interventions are assigned	
Implementation	16c	Who will generate the allocation sequence, who will enrol participants, and who will assign participants to interventions	
Blinding (masking)	17a	Who will be blinded after assignment to interventions (eg, trial participants, care providers, outcome assessors, data analysts), and how	Page 18
	17b	If blinded, circumstances under which unblinding is permissible, and procedure for revealing a participant's allocated intervention during the trial	

Methods: Data collection, management, and analysis

For peer review only - http://bmjopen.bmj.com/site/about/guidelines.xhtml

Data collection methods	18a	Plans for assessment and collection of outcome, baseline, and other trial data, including any related processes to promote data quality (eg, duplicate measurements, training of assessors) and a description of study instruments (eg, questionnaires, laboratory tests) along with their reliability and validity, if known. Reference to where data collection forms can be found, if not in the protocol	Page 18-19
	18b	Plans to promote participant retention and complete follow-up, including list of any outcome data to be collected for participants who discontinue or deviate from intervention protocols	Page 19
Data management	19	Plans for data entry, coding, security, and storage, including any related processes to promote data quality (eg, double data entry; range checks for data values). Reference to where details of data management procedures can be found, if not in the protocol	
Statistical methods	20a	Statistical methods for analysing primary and secondary outcomes. Reference to where other details of the statistical analysis plan can be found, if not in the protocol	Page 19-20
	20b	Methods for any additional analyses (eg, subgroup and adjusted analyses)	NA

	20c	Definition of analysis population relating to protocol non-adherence (eg, as randomised analysis), and any statistical methods to handle missing data (eg, multiple imputation)	
**Methods: Monitoring**			
Data monitoring	21a	Composition of data monitoring committee (DMC); summary of its role and reporting structure; statement of whether it is independent from the sponsor and competing interests; and reference to where further details about its charter can be found, if not in the protocol. Alternatively, an explanation of why a DMC is not needed	Page 20
	21b	Description of any interim analyses and stopping guidelines, including who will have access to these interim results and make the final decision to terminate the trial	N/A
Harms	22	Plans for collecting, assessing, reporting, and managing solicited and spontaneously reported adverse events and other unintended effects of trial interventions or trial conduct	Page 21
Auditing	23	Frequency and procedures for auditing trial conduct, if any, and whether the process will be independent from investigators and the sponsor	Page 21
**Ethics and dissemination**			

Research ethics approval	24	Plans for seeking research ethics committee/institutional review board (REC/IRB) approval	Page 22
Protocol amendments	25	Plans for communicating important protocol modifications (eg, changes to eligibility criteria, outcomes, analyses) to relevant parties (eg, investigators, REC/IRBs, trial participants, trial registries, journals, regulators)	Page 22
Consent or assent	26a	Who will obtain informed consent or assent from potential trial participants or authorised surrogates, and how (see Item 32)	Page 23
	26b	Additional consent provisions for collection and use of participant data and biological specimens in ancillary studies, if applicable	
Confidentiality	27	How personal information about potential and enrolled participants will be collected, shared, and maintained in order to protect confidentiality before, during, and after the trial	Page 23
Declaration of interests	28	Financial and other competing interests for principal investigators for the overall trial and each study site	Page 24
Access to data	29	Statement of who will have access to the final trial dataset, and disclosure of contractual agreements that limit such access for investigators	Page 25

For peer review only - http://bmjopen.bmj.com/site/about/guidelines.xhtml

Ancillary and post-trial care	30	Provisions, if any, for ancillary and post-trial care, and for compensation to those who suffer harm from trial participation	N/A
Dissemination policy	31a	Plans for investigators and sponsor to communicate trial results to participants, healthcare professionals, the public, and other relevant groups (eg, via publication, reporting in results databases, or other data sharing arrangements), including any publication restrictions	
	31b	Authorship eligibility guidelines and any intended use of professional writers	
	31c	Plans, if any, for granting public access to the full protocol, participant-level dataset, and statistical code	
**Appendices**			
Informed consent materials	32	Model consent form and other related documentation given to participants and authorised surrogates	Appendix
Biological specimens	33	Plans for collection, laboratory evaluation, and storage of biological specimens for genetic or molecular analysis in the current trial and for future use in ancillary studies, if applicable	

For peer review only - http://bmjopen.bmj.com/site/about/guidelines.xhtml

*It is strongly recommended that this checklist be read in conjunction with the SPIRIT 2013 Explanation & Elaboration for important clarification on the items. Amendments to the protocol should be tracked and dated. The SPIRIT checklist is copyrighted by the SPIRIT Group under the Creative Commons "Attribution-NonCommercial-NoDerivs 3.0 Unported" license.

For peer review only

For peer review only - http://bmjopen.bmj.com/site/about/guidelines.xhtml
